# Evolutionary Dynamics of the Repeatome Explains Contrasting Differences in Genome Sizes and Hybrid and Polyploid Origins of Grass Loliinae Lineages

**DOI:** 10.3389/fpls.2022.901733

**Published:** 2022-07-01

**Authors:** María Fernanda Moreno-Aguilar, Luis A. Inda, Aminael Sánchez-Rodríguez, Itziar Arnelas, Pilar Catalán

**Affiliations:** ^1^Escuela Politécnica Superior de Huesca, Universidad de Zaragoza, Huesca, Spain; ^2^Instituto Agroalimentario de Aragón, Universidad de Zaragoza, Centro de Investigación y Tecnología Agroalimentaria, Zaragoza, Spain; ^3^Departamento de Ciencias Biológicas y Agropecuarias, Universidad Técnica Particular de Loja, Loja, Ecuador; ^4^Grupo de Bioquímica, Biofísica y Biología Computacional, Instituto de Biocomputación y Física de Sistemas Complejos, Universidad de Zaragoza, Unidad Asociada al CSIC, Zaragoza, Spain

**Keywords:** diploidized paleo-allopolyploids, genome size diversification, Festuca, Lolium, phylogenetic signal, repeatome, transposable elements, 5S loci

## Abstract

The repeatome is composed of diverse families of repetitive DNA that keep signatures on the historical events that shaped the evolution of their hosting species. The cold seasonal Loliinae subtribe includes worldwide distributed taxa, some of which are the most important forage and lawn species (fescues and ray-grasses). The Loliinae are prone to hybridization and polyploidization. It has been observed a striking two-fold difference in genome size between the broad-leaved (BL) and fine-leaved (FL) Loliinae diploids and a general trend of genome reduction of some high polyploids. We have used genome skimming data to uncover the composition, abundance, and potential phylogenetic signal of repetitive elements across 47 representatives of the main Loliinae lineages. Independent and comparative analyses of repetitive sequences and of 5S rDNA loci were performed for all taxa under study and for four evolutionary Loliinae groups [Loliinae, Broad-leaved (BL), Fine-leaved (FL), and Schedonorus lineages]. Our data showed that the proportion of the genome covered by the repeatome in the Loliinae species was relatively high (average ∼ 51.8%), ranging from high percentages in some diploids (68.7%) to low percentages in some high-polyploids (30.7%), and that changes in their genome sizes were likely caused by gains or losses in their repeat elements. Ty3-gypsy Retand and Ty1-copia Angela retrotransposons were the most frequent repeat families in the Loliinae although the relatively more conservative Angela repeats presented the highest correlation of repeat content with genome size variation and the highest phylogenetic signal of the whole repeatome. By contrast, Athila retrotransposons presented evidence of recent proliferations almost exclusively in the *Lolium* clade. The repeatome evolutionary networks showed an overall topological congruence with the nuclear 35S rDNA phylogeny and a geographic-based structure for some lineages. The evolution of the Loliinae repeatome suggests a plausible scenario of recurrent allopolyploidizations followed by diploidizations that generated the large genome sizes of BL diploids as well as large genomic rearrangements in highly hybridogenous lineages that caused massive repeatome and genome contractions in the Schedonorus and Aulaxyper polyploids. Our study has contributed to disentangling the impact of the repeatome dynamics on the genome diversification and evolution of the Loliinae grasses.

## Introduction

Comparative genomic studies have demonstrated that the repetitive DNA fraction is largely present in the nuclear genome of most plants (Pellicer et al., 2018). It is composed of diverse families of mobile elements (retrotransposons and transposons), which constitute the bulk of the predominant repeats, and of tandem satellite repeats, which can make up 10–20% of the genome (Macas et al., 2015). Although the constitution of the repetitive elements is complex and differs, sometimes by some orders of magnitude, among taxa (Hidalgo et al., 2017), there is an overall agreement on the impact that the dynamics of the repetitive elements have had in the variation of the genome size and its evolution across the angiosperms (Dodsworth et al., 2015; Pellicer et al., 2018). Alternative hypotheses have been launched to explain both the causes and the mechanisms of the plant repeatome turnovers. The “polyploid genome shock” hypothesis that postulates genomic reshuffling and mobility of the repetitive elements in hybrid and polyploid plants as a response to the sudden combination of distinct genomes and multiple copies of them (McClintock, 1984) has resulted, in some cases, in a rapid increase of repeats in the genomes after rounds of polyploidizations. The resulting polyploid genomes show additive patterns and equivalent genome size expansions (McCann et al., 2018). However, other plants do not show a proliferation of the repetitive elements in the allopolyploids, or only a gradual and low increase or decrease in their derived subgenomes (Chen et al., 2020). In contrast, other plant groups have experienced the opposite trend, with high-level polyploids exhibiting a drastic reduction in genome size and a considerable shrinkage of their repeatome relative to that of their diploid and low-level polyploid relatives (Chen, 2007; Parisod et al., 2010). The removal of the repetitive elements from the genome, attributed to several recombination mechanisms, and the driven forces that balance the expansions and contractions of the repeatome are still poorly known (Fedoroff, 2012; Drouin et al., 2021). In some exhaustively studied plants (*Gossypium*, *Brachypodium*) the abundance of some retrotransposon families and their apparent facility to proliferate (e.g., centromeric transposons) are interpreted as causing increased genome size, while the ability of other families to recombine and lose repeats are considered potential mechanisms for maintaining reduced genome size (Chen et al., 2020; Stritt et al., 2020). The dynamics of some repetitive elements, especially transposable elements (TEs) insertions, has been also related to the expression of some core or dispensable genes, although their mobility does not seem to substantially affect their regulation (Gordon et al., 2017) but can be affected by epigenetic effects (Chen, 2007; Fedoroff, 2012; Negi et al., 2016).

A comprehensive repetitive DNA analysis of plant genomes is still hampered by the unavailability of assembled and annotated genomes for many groups with complex and large genomes (Michael, 2014). In most cases it has been circumvented by using genome skim approaches and repeatome graph-topology analysis (Weiss-Schneeweiss et al., 2015; Garcia et al., 2020). Several studies have demonstrated that similarity-based clustering of low coverage genome sequencing reads, which confidentially represent 0.50–0.01× of the total haploid genome coverage, is proportional to the genomic abundance and longitude of the corresponding repeat-types (Macas et al., 2015; Pellicer et al., 2018) and could therefore be used to quantify them. The utility of the Repeat Explorer 2 bioinformatics tools for the quantification and annotation of repeats in plants (Novák et al., 2020) has been implemented by phylogenetic and distance-based network methods and by multivariate statistical methods that have corroborated the phylogenetic signal of the repeatome in various groups of angiosperm (Vitales et al., 2020a,b; Herklotz et al., 2021). It has also been supplemented by 5S rDNA graph-based clustering methods which have successfully corroborated the identity of the ancestral progenitor genomes of several polyploid plants (Garcia et al., 2020; Vozárová et al., 2021).

The grass subtribe Loliinae (*Festuca* and other close genera, like *Lolium*) constitutes one of the main lineages of the temperate pooids, both in number of species and in ecological and economic importance (Catalán, 2006; Kopecký and Studer, 2014). The Loliinae include more than 600 accepted species, Catalán (2006; Plants of the World On-line^1^, accessed 3rd May 2022) which are distributed in cool seasonal and tropical mountainous regions of the five continents (Minaya et al., 2017; Moreno-Aguilar et al., 2020). The Loliinae species have large genomes ranging from 4.1 Gbp/2C to 23.6 Gbp/2C (Loureiro et al., 2007; Šmarda et al., 2008). Although these taxa show a uniform chromosome base number of *x* = 7 and ploidy levels ranging from diploids to dodecaplois, they exhibit striking differences in monoploid genome sizes, showing a 2.5-fold range decrease in chromosome size and C-values from more ancestral BL lineages (Drymanthele, Scariosae, Subbulbosae) to more recently evolved FL lineages (Festuca, Aulaxyper) (Catalán, 2006; Šmarda et al., 2008). In contrast, the heterochromatin pattern is inversely correlated with the genome size pattern, showing a rank increase of 7.5 between the same groups. However, this pattern is not homogeneous, as the early diverging fine-leaved Eskia lineage and the recently evolved broad-leaved Schedonorus-Lolium lineage revealed independent intermediate karyotype patterns between the BL and FL groups (Catalán, 2006). Genome size analyses of Loliinae and other close Poeae suggested that the ancestor of Loliinae probably underwent a two-fold genome size enlargement (and parallel GC enrichment) relative to its close relatives, which was later followed by dramatic reductions, especially in the rapidly evolving FL Loliinae group (Šmarda et al., 2008). Nonetheless, alternative scenarios could involve large genome size increase only in the BL lineage or parallelisms in the most ancestral BL and FL lineages (Catalán, 2006). A genome downsizing trend has been detected in the fine-leaved Loliinae and in the polyploids, for which more pronounced genome losses have been hypothesized to have occurred in allopolyploids with large progenitor genomes than in autopolyploids with small progenitor genomes (Loureiro et al., 2007; Šmarda et al., 2008). However, none of these hypotheses have been tested yet through genomic analyses. There is a general lack of knowledge on the repetitive elements of the Loliinae genomes except for some chromosome barcoding markers in meadow fescue (Křivánková et al., 2017; Ebrahimzadegan et al., 2019) and the characterization of repeats and centromeric elements in eight species of tall fescues and relatives (Zwyrtková et al., 2020). Apart from these works, no other study has exhaustively explored the composition and dynamics of repetitive elements through a complete representation of the Loliinae.

Here, we have investigated the repeatome of 47 representatives of all the phylogenetic lineages recognized so far within the Loliinae (Inda et al., 2008; Minaya et al., 2017; Moreno-Aguilar et al., 2020) aiming to elucidate the potential role of repeats in the striking differences in genome size and in the evolution of both genomes and species. The objectives of our study are: (i) to characterize and quantify the repetitive elements of representatives of the BL and FL Loliinae and identify single or preponderant repeats in some groups; (ii) to test the plausible correlation between genome size and abundance of the repeats; (iii) to identify repeat types that could have contributed to the expansions or contractions of genomes and their relationships with the ploidy levels, the nature of the polyploidy and the phylogenetic positions of the groups; (iv) to assess the phylogenetic value of repeats using phylogenetic reconstructions and phylogenetic signal approaches; and (v) to test alternative hypotheses about which lineages were affected by repeat proliferation or contraction and the putative paleo-hybrid origin of BL diploids with large genome sizes using mobile and satellite repeat data analysis.

## Materials and Methods

### Sampling, Cytogenetic Data and Genome Skim Sequencing

Forty-seven samples of diploid and polyploid taxa of Loliinae, representing its main broad-leaved (BL, 13 samples), fine-leaved (FL, 17) and Schedonorus (17) groups, were used in the study [Table 1 and Supplementary Table 1 (taxonomic ranks and authorships)]. Classification of samples into groups was based on previous phylogenetic frameworks (Minaya et al., 2017; Moreno-Aguilar et al., 2020). The sampling included taxa analyzed genomically for the first time within the BL (*Festuca scabra*, South African lineage; *F. mekiste*, Tropical Africa lineage) and FL (*F. rubra*, Aulaxyper lineage) groups plus the genome skim data generated in a previous study for representatives of other BL and FL lineages (Moreno-Aguilar et al., 2020). We obtained a large taxonomic representation of the Schedonorus group through the additional sequencing of species not studied molecularly (*F. dracomontana*, *F. gudoschnikovii*, *Lolium saxatile*) or genomically (*F. gigantea*, *F. simensis*, *Micropyropsis tuberosa*) before, and from a wide coverage of other tall fescues (*F. arundinacea*, *F. atlantigena*) and raygrasses (*L. canariense, L. perenne, L. persicum, L. rigidum*) (Table 1 and Supplementary Table 1). The 47 selected taxa represent the 20 evolutionary lineages currently recognized within the Loliinae (Minaya et al., 2017; Moreno-Aguilar et al., 2020). They constitute a suitable test-bed case for investigating the putative role of repeat type dynamism in the genomic evolution of the major Loliinae lineages and their contrasting changes in genome size (Catalán, 2006; Šmarda et al., 2008). They could be also used to assess the potential phylogenetic value of the repeat elements at the subtribal level.

**TABLE 1:** Taxa included in the repeatome analysis of Loliinae.

Taxon	Group	Locality	2*n*	Ploidy	2C(pg)	1Cx (pg)	1Cx (Mbp)	GenBank accession no.		
								Plastome	35S rDNA	5S rDNA
*Festuca africana*	BL	Uganda: Gahinga	70	10x	–	–	–	SAMN14647044	MT145277	**ON248974**
*Festuca amplissima*	BL	Mexico: Chihuahua	42	6x	–	–	–	SAMN14647045	MT145278	**ON248975**
*Festuca caldasii*	BL	Ecuador: Catamayo	**28**	4x	**20.36**	5.09	4978.02	SAMN14647047	MT145280	**ON248977**
*Festuca durandoi*	BL	Portugal: Serra Arga	14	2x	14.66 (4x)	3.66	3584.86	SAMN14647050	MT145283	**ON248980**
*Festuca lasto*	BL	Cádiz: Jerez	14	2x	–	–	–	SAMN14647058	MT145291	**ON248989**
*Festuca mekiste*	BL	Kenya: Mt. Elgon	–	–	–	–	–	**SAMN27777779**	**ON243855**	**ON248992**
*Festuca molokaiensis*	BL	United States: Hawai: Molokai	–	–	–	–	–	SAMN14647061	MT145294	**ON248993**
*Festuca paniculata*	BL	Spain: Caceres	14	2x	7.65	3.83	3740.85	SAMN14647064	MT145297	**ON248996**
*Festuca parvigluma*	BL	China: Baotianman	28	4x	–	–	–	SAMN14647065	MT145298	**ON248997**
*Festuca scabra*	BL	S Africa: Cathedral P.	28	4x	–	–	–	**SAMN27777781**	**ON243857**	**ON249003**
*Festuca spectabilis*	BL	Bosnia-H: Troglav	42	6x	–	–	–	SAMN14647071	MT145304	**ON249004**
*Festuca superba*	BL	Argentina: Jujuy	56	8x	–	–	–	SAMN14647072	MT145305	**ON249005**
*Festuca triflora*	BL	Morocco: Rif Mnts.	**14**	2x	**7.84**	3.92	3833.76	SAMN14647073	MT145306	**ON249006**
*Festuca abyssinica*	FL	Tanzania: Kilimanjaro	28	4x	–	–	–	SAMN14647043	MT145276	**ON248973**
*Festuca asplundii*	FL	Ecuador: Saraguro	42	6x	**21.23**	3.54	3460.49	SAMN14647046	MT145279	**ON248976**
*Festuca capillifolia*	FL	Morocco: Ifrane	14	2x	–	–	–	SAMN14647048	MT145281	**ON248978**
*Festuca chimborazensis*	FL	Ecuador: Chimborazo	**42**	6x	**13.48**	2.25	2197.24	SAMN14647049	MT145282	**ON248979**
*Festuca eskia*	FL	Spain: Picos de Europa	14	2x	5.7	2.85	2787.3	SAMN14647051	MT145284	**ON248981**
*Festuca fimbriata*	FL	Argentina: Apóstoles	42	6x	–	–	–	SAMN14647053	MT145286	**ON248983**
*Festuca francoi*	FL	Portugal: Terceira	12	2x	–	–	–	SAMN14647057	MT145290	**ON248984**
*Festuca gracillima*	FL	Argentina: Trra.Fuego	42	6x	–	–	–	SAMN14647055	MT145288	**ON248986**
*Festuca holubii*	FL	Ecuador: Saraguro	–	–	–	–	–	SAMN14647056	MT145289	**ON248988**
*Festuca ovina*	FL	Rusia: Gatchinskii Ra.	14	2x	4.82	2.41	2356.98	SAMN14647062	MT145295	**ON248994**
*Festuca pampeana*	FL	Argentina: Ventana	56	8x	–	–	–	SAMN14647063	MT145296	**ON248995**
*Festuca procera*	FL	Ecuador: Chimborazo	28	4x	**14.88**	3.72	3638.16	SAMN14647067	MT145299	**ON248999**
*Festuca pyrenaica*	FL	Spain: Tobacor	28	4x	–	–	–	SAMN14647068	MT145300	**ON249000**
*Festuca pyrogea*	FL	Argentina: Trra.Fuego	–	–	–	–	–	SAMN14647069	MT145302	**ON249001**
*Festuca rubra*	FL	Argentina: Trra.Fuego	42	**6x**	13.68	2.28	2229.84	**SAMN27777780**	**ON243856**	**ON249002**
*Megalachne masafuerana*	FL	Chile: Masafuera	–	–	–	–	–	SAMN14647075	MT145308	**ON249018**
*Vulpia ciliata*	FL	Spain: Ontígola	28	4x	8.28	2.07	2024.46	SAMN14647076	MT145309	**ON249009**
*Festuca a. arundinacea*	Sch	Spain: Ferrol	42	6x	17.46	2.91	2845.98	**SAMN27777774**	**ON243850**	**ON249007**
*Festuca a. atlantigena*	Sch	Morocco: Atlas Mnts	56	8x	16.22	2.03	1982.895	**SAMN27777775**	**ON243851**	**ON248990**
*Festuca a. letourneuxiana*	Sch	Morocco: Atlas Mnts	70	10x	19.7	1.97	1926.66	SAMN14647059	MT145292	**ON249010**
*Festuca dracomontana*	Sch	SAfrica:Haernertsburg	–	–	–	–	–	**SAMN27777776**	**ON243852**	**ON249011**
*Festuca fenas*	Sch	Spain	28	4x	10.48	2.62	2562.36	SAMN14647052	MT145285	**ON248982**
*Festuca fontqueri*	Sch	Morocco: Rif Mnts	14	2x	**5.54**	2.77	2709.06	SAMN14647054	MT145287	**ON249008**
*Festuca gigantea*	Sch	Norway	42	6x	20.75	3.46	3382.25	**SAMN27777777**	**ON243853**	**ON248985**
*Festuca gudoschnikovii*	Sch	Russia: Yermakovskii	28	4x	–	–	–	**SAMN27777778**	**ON243854**	**ON248987**
*Festuca mairei*	Sch	Morocco: Atlas Mnts	28	4x	10.04	2.51	2454.78	SAMN14647060	MT145293	**ON248991**
*Festuca pratensis*	Sch	United Kingdom: England	14	2x	6.5	3.25	3178.5	SAMN14647066	MT145301	**ON248998**
*Festuca simensis*	Sch	Kenya: Mt. Kenya	28	4x	–	–	–	**SAMN27777782**	**ON243858**	**ON249012**
*Lolium canariense*	Sch	Spain: Canary Islands	14	2x	4.3	2.15	2102.7	**SAMN27777783**	**ON243859**	**ON249013**
*Lolium perenne*	Sch	United Kingdom: Wales	14	2x	5.51	2.76	2694.39	**SAMN27777784**	**ON243860**	**ON249014**
*Lolium persicum*	Sch	Georgia	14	2x	6.4	3.2	3129.6	**SAMN27777785**	**ON243861**	**ON249015**
*Lolium rigidum*	Sch	Turkey	14	2x	5.49	2.75	2684.61	**SAMN27777786**	**ON243862**	**ON249017**
*Lolium saxatile*	Sch	Spain: Fuerteventura	14	2x	–	–	–	**SAMN27777787**	**ON243863**	**ON249016**
*Micropyropsis tuberosa*	Sch	Spain: Almonte	14	2x	–	–	–	**SAMN27777788**	**ON243864**	**ON249019**

*Loliinae group (BL, broad-leaved Loliinae; FL, fine-leaved Loliinae; Sch, Schedonorus), chromosome number (2n), ploidy level, genome size (2C, pg), monoploid genome size (1Cx, pg; 1Cx, Mbp) and GenBank accession codes for plastome and nuclear ribosomal 35S and 5S genes are given for each sample. Values in bold correspond to new data generated in this study. Hyphens indicate lack of 2n and/or 2C/1Cx data for some taxa. See Supplementary Table 1 for additional information on taxonomic ranks and taxon authorship, detailed localities and vouchers, and sources of cytogenetic and genomic data.*

Cytogenetic knowledge of Loliinae taxa varies enormously. Besides relatively well scrutinized groups of economic importance, like some members of the Schedonorus, Aulaxyper, and Festuca lineages (Catalán et al., 2004; Šmarda et al., 2008; Minaya et al., 2017), cytogenetic data are missing for other species, especially for taxa from poorly studied taxonomic groups or less explored areas (Catalán, 2006). Chromosome number (2*n*) and genome size (2C/pg) data were estimated for some of the studied samples using DAPI-stained meristematic root cells and flow cytometry analysis following the protocols of Jenkins and Hasterok (2007) and Doležel et al. (2007), respectively. Chromosome staining was performed with the DAPI fluorescent marker (4′,6-diamino-2 phenylindole) and counts were done using a Motic BA410 fluorescence microscope. The nuclear DNA content of *F. asplundii, F. caldasii, F. chimborazensis, F. fontqueri* and *F. procera* were calculated from silica gel dried leaves using nuclei isolated from similarly processed leaves of *Pisum sativum* L. “Ctirad” (9,09 pg/2C) as standard. Nuclei were stained with propidium iodide and samples were analyzed using a CyFlow Ploidy Analyser SYSMEX. At least 5,000 nuclei were analyzed per sample and each sample (two replicates) was analyzed three times. Only measurements with coefficient of variation < 3.5% were recorded. Ploidy levels were inferred from chromosome counts (2*n*) and GS estimations performed in the same accessions used in our genomic study and through contrasted GS and 2*n* values obtained in conspecific accessions that showed similar values. However, cytogenetic data is still lacking for some unstudied species that could only be analyzed genomically using museomic approaches (Moreno-Aguilar et al., 2020; Table 1 and Supplementary Table 1).

Total DNA for the 15 newly sampled Loliinae taxa was extracted from herbarium specimens (MHU, PRE, UZ, VLA) and silica gel dried leaf tissues from plants growing in the University of Zaragoza – High Polytechnic School of Huesca common garden (Supplementary Table 1). Isolation of DNA and its concentration quantification and quality evaluation for genome skimming sequencing was performed following the procedures indicated in Moreno-Aguilar et al. (2020). PCR free libraries were quantified by Library Quantification Kit for Illumina Platforms (Roche Kapa Biosystems). Genomic sequencing of a multiplexed pool of KAPA libraries was performed on a HiSeq4000 or HiSeq 2500 (TruSeq SBS Kit v4, Illumina, Inc.) in paired-end mode (2 × 100 bp) in the Centro Nacional de Análisis Genómicos (CNAG, Barcelona) as described in Moreno-Aguilar et al. (2020). Illumina paired-end (PE) reads were checked using FASTQC and the adapters and low quality sequences were trimmed and removed using TRIMMOMATIC (Bolger et al., 2014). The Loliinae genomic samples used in downstream analysis contained between 6.1 and 40.6 million reads (average 18.0 million reads) with insert sizes ranging between 190 and 300 bp (Supplementary Table 2).

### Repeat Clustering and Annotation, and 5S rDNA Graph-Clustering Analysis

Identification of the composition and proportion of repetitive elements in the 47 Loliinae species studied was performed from similarity graph-based clustering analysis of filtered PE reads using the Repeat Explorer pipeline of RepeatExplorer2 (RE2)^2^. It was performed through the Galaxy platform as described by Novák et al. (2020). The clustering analysis of individual samples was fed with 500000 PE reads per sample in order to attain the recommended genome coverage (0.1–0.5×) of each taxon (Supplementary Table 2). The clustering was conducted employing default RE2 settings (90% similarity, minimum overlap = 55; cluster size threshold = 0.01%) and long queue (max runtime). Automated RE2 annotation of clusters was used to quantify the clusters and to calculate the proportions of repetitive elements in each sample. Plastid and mitochondrial DNA clusters were removed prior to downstream analyses. Comparative clustering analysis was performed for four evolutionary groups (Loliinae, BL, FL, Schedonorus) due to the impossibility of computing it for all the studied samples (47) in a single run of Galaxy employing the same RE2 configuration used for the individual analyses. The Loliinae group was reduced to 38 samples, representing all its main lineages, while the BL, FL and Schedonorus groups contained the same samples used in the individual analysis except the BL group which had two additional Schedonorus samples (Table 1 and Supplementary Tables 1, 2). The comparative clustering analyses were conducted using the maximum number of randomly sampled PE reads that could be processed, representing ∼0.08–0.2× of genome coverage for each species (Supplementary Table 2). Automated RE2 repeat annotation was used to quantify the clusters and to estimate the proportions of repeats among the compared samples within each group. Plastid and mitochondrial DNA clusters were also removed from each group prior to downstream analyses.

Sequences of 5S ribosomal DNA genes from 43 out of the 47 studied Loliinae samples were searched using the TAREAN pipeline of RE2 (Garcia et al., 2020; Novák et al., 2020). The input for the 5S rDNA clustering analysis consisted of 500000 PE reads per sample, covering the expected lengths of the 5S rDNA for most of the Loliinae genomes ranging 4.2–20.7 Gbp (Supplementary Table 1). The clustering was performed using default TAREAN tool settings (BLAST threshold of 90%, similarity across 55% of the read to identify reads to each cluster, minimum overlap = 55, cluster threshold = 0.01%, minimum overlap for assembly = 40). The 5S rDNA clusters were found in the TAREAN tandem reports. Their shapes were characterized by a connected component index parameter (C) and their k-mer score was calculated as the sum of frequencies of all k-mers used for consensus sequence reconstruction (Garcia et al., 2020). The 5S rDNA cluster graph topologies were visually inspected and classified into graph groups (type 1, simple circular-shaped graph; type 2, complex graph with two or more loops where the interconnected loops represent IGS spacers) (Garcia et al., 2020). We examined the 5S graphs to detect potential variation of 5S rDNA loci and to identify presumable hybrids and allopolyploids. A RE2 5S rDNA sequence of *Festuca pratensis* (360 bp) was used as reference for a Geneious Prime read-mapping assembly of the 5S rDNA of the four Loliinae species (*F. caldasii, F. gigantea, F. gracillima, F. gudoschnikovii*) that could not be retrieved directly from TAREAN due to insufficient number of reads in the cluster for graphical analysis (see Table 4). Newly generated 5S rDNA sequences of Loliinae were deposited in GenBank under accessions codes ON248974–ON249019.

### Plastome and Nuclear rDNA Phylogenies of Loliinae

Genome skimming PE reads were used to assemble and annotate the plastomes and the nuclear 35S rDNA of the newly sequenced Loliinae samples (Table 1). Plastome assembly was performed with Novoplasty v.2.7.1 (Dierckxsens et al., 2017) following the procedures indicated in Moreno-Aguilar et al. (2020) and using as reference the *Festuca pratensis* plastome sequence (JX871941). The 35S rDNA cistron (transcribed region ETS-18S-ITS1-5.8S-ITS2-25S) was assembled using the read-mapping and merging strategy of Moreno-Aguilar et al. (2020) using Geneious Prime and the *F. ovina* 35S rDNA sequence (MT145295) as reference. Newly generated plastome and 35S rDNA sequences of Loliinae were deposited in Genbank under accessions codes SAMN27777779–SAMN27777788 and ON243855–ON243864 (Table 1). Multiple sequence alignments (MSAs) of these sequences, together with those of the previously studied Loliinae samples and the *Oryza sativa* and *Brachypodium distachyon* outgroups (Supplementary Table 1), were performed with MAFFT v.7.031b (Katoh et al., 2002), visually inspected with Geneious Prime and debugged with trimAl v.1.2rev59 (imposing parameter-*automated*1) (Capella-Gutiérrez et al., 2009). The filtered plastome (133552 bp) and 35S rDNA cistron (6431 bp) MSA data sets were used to compute Maximum likelihood (ML) phylogenetic trees with IQTREE (Nguyen et al., 2015). Independent ML searches were performed imposing the best-fit nucleotide substitution model selected by ModelFinder for each partition, according to the Bayesian Information Criterion (BIC), and branch support for the best tree was estimated from 1,000 ultrafast bootstrap replicates (BS) (Chernomor et al., 2016; Kalyaanamoorthy et al., 2017).

The well resolved plastome and 35S ML trees were topologically contrasted to each other using the Kishino-Hasegawa (KH), Shimodaira-Hasegawa (SH), and Shimodaira Approximately Unbiased (AU) tests with resampling estimated log-likelihood (RELL) optimization and one million bootstrap replicates in PAUP* (Swofford, 2003). As all the pairwise tests showed that each topology did not significantly differ (*p* < 0.001) from the other topology, we constructed a combined ML plastome + 35S tree with IQTREE imposing the respective nucleotide substitution model to each partition and the procedures indicated above. To account for potential incomplete lineage sorting (Kubatko and Degnan, 2007) and to investigate the possibility that a single concatenated plastome + 35S data set could generate topological errors in the phylogeny, we run a parallel phylogenetic analysis with the same data set but modeling the coalescence process using the Singular Value Decomposition quartets (SVDq) approach implemented in Paup*, which uses a variant of Quartet FM (Reaz et al., 2014) to combine quartet trees into a species tree. We imposed the SVDQuartets nquartets = all seed = 2 nthreads = 4 bootstrap = 1000 options with a multispecies coalescent tree model and the quartet assembly algorithm QFM. Bootstrap support of branches was shown on the tree obtained from SVDquartests + Paup* analysis. Since the topology of the SVDq tree (Supplementary Figure 1A) was equal to that of the ML tree (Supplementary Figure 1B), we selected the strong to relatively well supported ML tree for downstream analysis. Different ML subtrees were computed from the whole combined plastome + 35S data matrix using the respective subsets of taxa of each of the four Loliinae evolutionary groups employed in the repeatome analyses (Loliinae, BL, FL, Schedonorus). These ML tree cladograms were used to estimate the phylogenetic signal of the repeats of each partition (see below). A MSA was also generated for the 5S rDNA sequences of Loliinae and close outgroups (Supplementary Table 1) and a ML phylogenetic tree was computed with this data set following the procedures indicated above.

### Repeatome Trees and Evolutionary Networks of Loliinae, Phylogenetic Signal of Repeats

Evolutionary analyses were performed with the repeat data obtained from the comparative clustering of repeats for the Loliinae, BL, FL and Schedonorus groups. Distance-based phylogenetic trees and networks were computed from pairwise genetic distances between the repeat contents of the species included in the datasets. First, calculated repeat sequence similarity matrices for the observed/expected number of interspecies edges for each of the most abundant repeat clusters selected by RE2 were converted to Euclidean distances via the *dist* option of the *proxy* package in R (Euclidean matrices). Second, the same repeat sequence similarity matrices were transformed into distance matrices by calculating the inverse of their values as described by Vitales et al. (2020b) (inverse matrices). In both cases, the clusters with incomplete information (NA or zero values) for the similarity comparisons between species pairs were discarded from the analysis. Next, Neighbor-Joining phylogenetic trees were constructed for each repetitive element using either the Euclidean or the inverse distance matrices and the *NJ* function of *ape* package (Paradis et al., 2004) in R. Finally, consensus networks were built from all the repeat NJ trees with SplitsTree4 (Huson and Bryant, 2006) for each group.

The combined plastome + 35S ML subtrees were used to test the potential phylogenetic signal of different types of repeats of each group using Blomberg’s *K* (Blomberg et al., 2003) with the *phylosig* function of the package *phytools* (Revell, 2012) in R. For these tests, *K* values > 1 indicate that the repeatome traits have more phylogenetic signal than expected, values ∼1 that traits are consistent with the tree topology (phylogenetic signal), and values ∼0 that there is no influence of shared ancestry on trait values (phylogenetic independence).

### Correlations of Repeat Amounts and Genome Size Variation and Global Diversity Analysis of Repeat Types in Loliinae

The potential contribution of the various groups of repeat types and the repeatome to the variation in genome size (1Cx) observed between and within Loliinae lineages was tested using the data from the comparative analysis and by linear regression model analyses (Pearson correlation coefficient) with the *ggscatter* function from the *ggpubr* package in R. The respective contributions of repeats to pairwise differences in genome sizes were estimated following Macas et al. (2015). To correct for potential phylogeny-based bias, phylogenetically independent contrasts (PIC) methods were previously applied to the data using the *pic* option of the *ape* package in R. Correlations could be only performed for the 23 Loliinae species with known genome size (Table 1), representing all the main subtribal groups, and using absolute amounts (Mbp) of repeats calculated for individual species (Supplementary Table 1). In addition, we also tested whether there were significant differences in repeat amount for different repeat families obtained from the individual analysis through Kruskal–Wallis rank tests using the *multcompView* and *ggpubrr* packages in R. Furthermore, to investigate the levels of conservatism or diversity of the repeat types that most contributed to genome size variation in Loliinae (23 species with known genome sizes) we performed a genome landscape search for the global variability of these individual repeat types across the Loliinae genomes. We pooled the pairwise similarity values of reads, retrieved from the RE2 outputs (hitsort files), for each species and repeat type in a separate dataset and evaluated their similarities with respect to similarities of reads from the same repeat in all other species following Macas et al. (2015). We calculated intraspecific versus interspecific similarity hit ratios (Hs/Ho ratios) considering that conservative sequence repeats will produce similarity hits with about the same frequency for Hs and Ho, while diversified sequence repeats will generate similarity hits with different frequencies. We also calculated similarity hit ratios for the 5S tandem-repeat rDNA to compare its gene-conserved vs. IGS-variable Hs/Ho ratios with those obtained from the other repeat elements analyzed.

## Results

### Multiple Polyploidizations and Genome Size Diversification Across the Phylogeny of Loliinae

**FIGURE 1:**
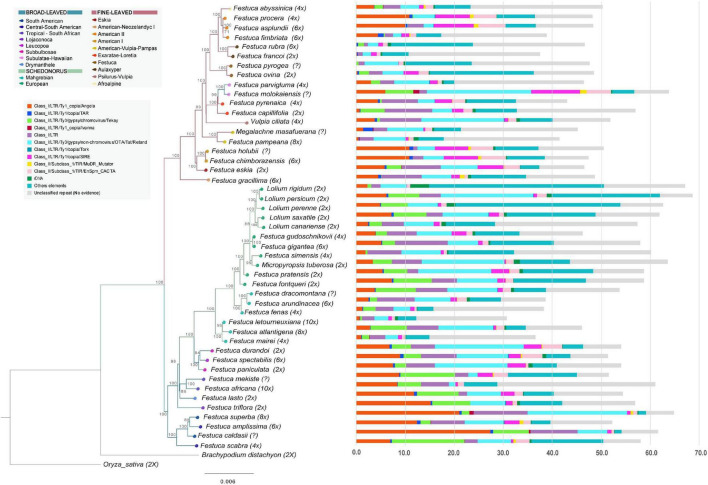
Histograms of repeat contents per holoploid genome (1C) retrieved from the individual Repeat Explorer 2 analyses of the studied Loliinae samples mapped onto the Maximum Likelihood combined phylogenomic tree (plastome + nuclear 35S rDNA cistron) of Loliinae (color codes of Loliinae lineages are indicated in the chart). Color codes for repeat families are indicated in the corresponding inset charts. Scale bar: number of mutations per site.

Chromosome counts and genome size data obtained for, respectively, 41 and 23 out of the 47 Loliinae taxa studied (Table 1) corroborated previous records but also revealed new findings about contrasting genome sizes between and within the BL and FL Loliinae lineages when mapped to the combined Loliinae tree (Figure 1 and Supplementary Figure 1B). The inferred ploidy levels for the newly analyzed South American *F. asplundii* (6x), *F. caldasii* (4x), *F. chimborazensis* (subsp. *micacochensis*, 6x) and *F. procera* (4x) species (Table 1) confirmed the lack of Loliinae diploids in the southern hemisphere (Dubcovsky and Martínez, 1992; Catalán, 2006). Genome sizes ranged from 4.3 Gbp (*L. canariense*-2x; Schedonorus) and 4.82 Gbp (*F. ovina*-2x; FL) to 21.23 Gbp (*F. asplundii*-6x; FL), representing a near 5-fold (x4.9) increase within the Loliinae and the FL group. Monoploid genome sizes ranged from 2.02 Gbp (*V. ciliata*-4x; FL) to 4.98 Gbp (*F. caldasii*-4x; BL), representing a ×3.7 increase within the Loliinae (Table 1 and Supplementary Table 1). Within the diploids, the broad-leaved species showed 2C genome sizes (*F. triflora*, 7.67 Gbp; *F. paniculata*, 7.48) 1.5x larger than those of the fine-leaved *Festuca* (*F. ovina*, 4.71) and some *Lolium* (*L. perenne*, 4.2) species, while the early diverging fine-leaved *F. eskia* (5.57) and other Schedonorus species (*F. fontqueri*, 5.52; *F. pratensis*, 6.36; *L. perenne*, 5.39; *L. rigidum*, 5.4; *L. persicum*, 6.26) displayed intermediate GS values between them (Table 1 and Supplementary Table 1). A general trend of reduction in monoploid genome size was observed in some polyploid FL and Schedonorus taxa, showing lower values as ploidy level increased (FL: Aulaxyper: *F. rubra*-6x, 2.23 Gbp; American I: *F. chimborazensis*-6x, 2.2; Schedonorus: *F. arundinacea*-6x, 2.84; *F. atlantigena*-8x, 2.0; *F. letourneuxiana*-10x, 1.93). However, large 1Cx sizes were also detected among polyploid South-American Loliinae species nested either within the BL (Central and South American: *F. caldasii*-4x, 4.98) or the FL (American II: *F. procera*-4x, 3.64; *F. asplundii*-6x, 3.46) clades (Table 1, Supplementary Table 1, and Figure 1).

The combined plastome + 35S rDNA ML tree (Figure 1 and Supplementary Figure 1B) was overall congruent with the phylogenies of Minaya et al. (2017) and Moreno-Aguilar et al. (2020) for the divergences of the main Loliinae lineages. The combined tree retrieved a robust topology which was also congruent with those of the well supported plastome and less supported 35S rDNA trees (Supplementary Figures 1B–D). The Loliinae phylogeny showed the split of the sister BL and FL clades (Figure 1) and divergences within the clades similar to those indicated in Moreno-Aguilar et al. (2020) except for the position of the BL Subulatae-Hawaiian lineage which was nested within the FL clade in the current tree (Figure 1). The largely sampled Schedonorus clade showed the branching-off of the ‘Mahgrebian’ and ‘European’ sister clades; the latter included the split of the *Festuca* gr. *arundinacea* allopolyploids from the rest, although their respective nesting positions swapped between their ‘European’ plastome and ‘Mahgrebian’ 35S rDNA trees (Figure 1 and Supplementary Figures 1B–D). The remaining Schedonorus lineages of the ‘European’ clade showed the early divergences of diploids followed by those of polyploids and a reversal trend to diploidization in the recently split *Lolium* clade (Figure 1). Diploid and polyploid lineages were spread across the BL and FL clades of the Loliinae tree (Figure 1). Although several of the early diverging BL lineages are predominantly or uniquely made up of diploids (Drymanthele, Lojaconoa, Subbulbosae), other early splits contain exclusively low-to-high polyploids (South African, Central-South American). A similar trend of more ancient to more recent origins of polyploids could be observed within the Schedonorus and FL clades. Low-to-high polyploids have evolved in all FL lineages and several of them are formed exclusively by polyploids (American-Neozeylandic, American I, American-Pampas, Psilurus-Vulpia, Subulatae-Hawaiian, American II, Afroalpine) (Figure 1).

### The Loliinae Repeatome

The annotated repeats found by RE2 in the individual analyses showed large differences in repeat types and amounts among the 47 Loliinae samples and lineages (Table 2, Supplementary Table 2, Figure 1, and Supplementary Figure 1E). The proportion of the holoploid genome occupied with repeats ranged from 30.69% (*F. letourneuxiana*-10x) to 68.8% (*L. persicum*-2x), with a mean across Loliinae of 51.8% (Table 2, Figure 1, and Supplementary Figure 1E). The highest percentages corresponded to diploid taxa of the Schedonorus group (e.g., *Lolium* spp., *M. tuberosa*, *F. simensis*; >60%) and diploid or polyploid taxa of the BL group (e.g., *F. lasto*-2x; *F. triflora*-2x, *F. scabra*-4x, Central-South American spp.-4x-6x, *F. africana*-10x, plus FL *F. molokaiensis*; >57%) and the lowest to high-polyploid taxa of the Schedonorus group (Mahgrebian-4x-10x, *F. arundinacea*-6x; <40%) and to diploid and high-polyploid species of the FL Aulaxyper group (*F. francoi*-2x, *F. rubra*-6x; <46%) (Table 2; Figure 1, and Supplementary Figure 1E). LTR-Gypsy and LTR-Copia retrotransposons represented the major fractions of repeatome in the studied genomes followed by Class II TIR-transposons and Satellite repeats (Table 2 and Supplementary Figure 1E).

**TABLE 2:** Genome proportion of repeats estimated by Repeat Explorer 2 for individual Loliinae samples (estimations per holoploid genome, 1C).

Loliinae taxon and phylogenetic group	Class I/LTR/Ty1_copia								Class I/LTR/Ty3_gypsy									Class II/Subclass_1/TIR											
	Ale	Angela	Ikeros	Ivanna	SIRE	TAR	Tork	Ty1_Copia	OTA	Athila	Tat	Ogre	Retand	CRM	Tekay	Reina	Ty3_Gypsy	EnSpm_CACTA	Hat	MuDR-Mutator	PIF – Harbinger	rDNA (5S-45S)	Satellite	Mobile element	Class I/LTR (conflict evidence)	Class I/LINE	Repeat (conflicting evidences)	Unclassified (No evidence)	Total (%)
**Broad-Leaved**																													
*Festuca africana* Tropical-South African	0	8.42	0	0	0.46	0.06	0	0	0	0.3	0	4.63	1.32	0.14	4.75	0	0	1.14	0	0.13	0	0.04	1.7	0	5.74	0	0	32.26	61.1
*Festuca amplissima* Central-South American	0	12.43	0.05	0.11	3.04	0.71	0.23	0	0	0.26	0	0.02	7.87	0.67	2.05	0	0	1.54	0	0.79	0	0.12	2.72	0	6.89	0.16	0	12.58	52.25
*Festuca caldasii* Central-South American	0.03	27.45	0	0.33	0.82	0.48	0.02	0	0	0.26	0	0.13	6.03	0.17	7.32	0	0	0.49	0	0.11	0	0.06	0.88	0	9.64	0	0	7.4	61.63
*Festuca durandoi* Subbulbosae	0	6.81	0	0	3.6	0.47	0	0	0	0.17	0	0.16	18.2	1.84	3.87	0	0	4.04	0	0.19	0.09	0.1	0.96	0	4.9	0.02	0.87	7.81	54.12
*Festuca lasto* Drymanthele	0	11.83	0.09	0	2.73	0.59	0.02	0	0	3.42	0	0	6.85	1.76	8.51	0	0	1.72	0	0.03	0	0.17	0.84	0	1.76	0.01	0	14.11	54.46
*Festuca mekiste* Tropical-South African	0	8.79	0	0.01	3.08	0.24	0	0	0	2.77	0	7.92	1.91	0.35	11.14	0	0	2.86	0	0.27	0	0.03	3.01	0	2.68	0	0	6.5	51.57
*Festuca molokaiensis* Subulatae-Hawaiian	0	5.94	0.03	1.26	9.96	0.03	0	0	0	0.01	0	1.55	21.35	0.26	5.71	0	0	4.95	0	1.37	0	0.25	1.12	0	1.49	0.02	1.49	7.09	63.85
*Festuca paniculata* Subbulbosae	0	7.51	0	0	3.75	0.43	0.02	0	0	0.45	0	0	14.83	0.81	2.3	0	0	0.51	0	0.02	0.03	0.89	0.82	2.77	5.82	0.03	0	13.16	54.14
*Festuca parvigluma* Subulatae-Hawaiian	0	2.99	0.12	0	1.57	0.04	0.15	0	0	0.03	0	0.01	7.16	0.65	1.93	0	0	0.82	0	0.07	0	0.38	1.34	0	2.79	0	0	26.43	46.47
*Festuca scabra* South African	0	6.95	0.09	0.11	0.47	0.36	0	0	0	5.69	0	4.6	6.86	1.28	14.78	0	0	2.81	0	0.54	0	0.4	2.96	0	2.57	0	0	7.61	58.08
*Festuca spectabilis* Leucopoa	0	8.94	0	0	2.47	0.73	0.1	0	0	0.07	0	0.12	10.48	2.38	3.54	0	0	4.13	0	0.35	0.05	0.77	2.04	0	7.32	0.2	0	7.71	51.41
*Festuca superba* Central-South American	0.03	21.07	0	0.9	0.68	0.49	0.01	0	0	0	0	0.01	20.31	0.05	1.54	0	0	0.92	0	0.51	0	0.26	1.4	0	11.01	0	0	5.71	64.9
*Festuca triflora* Lojaconoa	0	15.24	0	0	1.34	0.33	0.14	0	0	5.98	0	0.11	7.15	0.78	7.71	0	0	0.85	0	0.13	0	0.51	1.78	0	0	0	0	14.93	56.98
**Schedonorus**																													
*Festuca a. arundinacea* F.gr.arundinacea	0	2.52	0.06	0	1.36	0.29	0.01	0	0	3.07	0	0.08	7.31	1.27	1.47	0	0	1.92	0	0.03	0.07	0.63	1.53	0	7.66	0.09	0.24	9.09	38.67
*Festuca a. Atlantigena* F.gr.arundinacea	0	2.84	0.02	0	0.6	0.08	0.01	0	0	0.14	0	0	11.16	1.49	7.5	0	0	1.62	0	0.06	0.03	0.37	2.13	0	6.42	0.08	0.09	11.43	46.09
*Festuca dracomontana* F.gr.arundinacea	0	3.79	0.03	0	1.05	0.18	0.01	0	0	1.58	0	0.13	10.24	2.45	8.23	0	0	1.68	0	0	0	0.59	1.49	0	6.44	0.04	0.79	15.09	53.82
*Festuca fenas* Mahgrebian	0	1.29	0.02	0	0.83	0.16	0	0	0	1.15	0	0	3.4	0.8	2.5	0	0	0.45	0	0	0	0.21	1.21	0	3.69	0.02	0	22.64	38.38
*Festuca fontqueri* European	0	7.31	0.09	0	1.65	0.28	0.01	0	0	7.55	0	0.63	8.21	2	7.9	0	0	1.54	0	0.08	0.01	0.09	3.25	0	5.12	0.03	1.11	11.96	58.82
*Festuca gigantea* European	0	5.16	0	0	0.98	0.13	0.01	0	0	0.98	0	0	6.19	3.73	2.62	0	0	1.19	0	0	0.03	0.4	8.06	0	10.76	0.1	0	17.62	57.96
*Festuca gudoschnikovii* European	0	3.96	0	0	3.02	0.12	0	0	0	0.19	0	0	7.17	3.37	2.22	0	0	1.24	0	0	0.02	0.59	5.32	0	6.04	0.07	0	12.93	46.25
*Festuca a. letourneuxiana* Mahgrebian	0	0.73	0.01	0	0.71	0.08	0	0.12	0.01	1.13	0	0	2.85	0.8	0.43	0	0.01	0.62	0	0	0.01	0.63	1.61	0	2.53	0.02	0	18.39	30.7
*Festuca mairei* Mahgrebian	0	1.02	0.03	0.02	0.82	0.18	0	0.1	0	1.32	0	0	2.59	0.99	1.51	0	0	0.62	0	0	0	0.3	2.28	0	3.19	0	0	21.58	36.57
*Festuca pratensis* European	0.04	5.41	0.01	0	3.77	0.19	0	0	0	7.18	0	0.66	14.88	4.26	4.89	0	0	2.02	0	0.01	0	0.69	1.81	0	2.17	0.01	0.4	10.29	58.72
*Festuca simensis* European	0	1.9	0	0	0.62	0.01	0	0.02	0.02	0.37	0.02	0	8.04	0.91	0.4	0	0	0.66	0	0	0	0.3	12.01	0	6.95	0.01	0	28.01	60.23
*Lolium canariense* Lolium	0.11	2.49	0	0	1.22	0.23	0	0	0	0.05	0	0.39	6.04	2.6	2.53	0	0	0.41	0	0.02	0	0.81	6.93	0	4.53	0	0	29.09	57.46
*Lolium perenne* Lolium	0.07	4.9	0	0	0.64	0.17	0.01	0	0	25.26	0	2.79	6.12	1.75	5.47	0	0	1.09	0	0.04	0.09	1.83	2.03	0	0	0.04	1.64	8.68	62.63
*Lolium persicum* Lolium	0.11	6.2	0	0	0.73	0.41	0.04	0	0	9.18	0	1.15	18.86	5.15	6.34	0	0	1.97	0	0	0.19	1.02	4.87	0	4.33	0	1.52	6.65	68.71
*Lolium rigidum* Lolium	0.1	2.29	0	0	0.14	0.04	0	0	0	23.1	0	4.86	5.3	2.68	0.79	0	0	0.63	0	0	0.06	3.83	2.53	0	1.85	0	2.42	16.53	67.15
*Lolium saxatile* Lolium	0.18	7.25	0.04	0	2.13	0.44	0.02	0.01	0	7.23	0	0	9.39	1.57	6.67	0.01	0	1.03	0	0	0.65	0.56	1.76	0	3.29	0.02	6.64	13.03	61.91
*Micropyropsis tuberosa* European	0.05	3.38	0	0	0.33	0.01	0	0	0	0.02	0	0.05	16.89	3.58	3.93	0	0	1.5	0	0.02	1.02	1.3	3.99	0	6.07	0	1.47	20.01	63.64
**Fine-Leaved**																													
*Festuca abyssinica* Afroalpine	0	3.65	0	0.07	0.96	0.08	0	0	0	0.15	0	1.85	3.83	0.34	1.78	0	0	1.56	0	0.41	0	0.31	4.93	0	3.45	0	0	26.92	50.27
*Festuca asplundii* American II	0	9.97	0.52	0	8.17	0.53	0.13	0	0	1.31	0	0.82	1.96	0.67	0.02	0	0	5.52	0	0.93	0.01	0.04	1.83	0	3.27	0	0.97	11.75	48.41
*Festuca capillifolia* Exaratae	0	0.75	0	0	2.32	0.14	0.01	0	0	1.58	0	0.22	4.03	1.41	4.87	0	0	1.86	0	0	0	0.65	5.16	0	9.77	0	0	24.23	57.02
*Festuca chimborazensis* American I	0	10.89	0.06	0.02	6.98	0.67	0	0	0.01	1.06	0	0.06	4.17	1.16	0	0	0	5.04	0	0.65	0.09	0.3	4.09	0	2.15	0	1.07	8.99	47.45
*Festuca eskia* Eskia	0	5.77	0.02	0.06	5.36	0.4	0	0	0	0.73	0	0.18	7.51	0.72	3.13	0	0	3.18	0	0.09	0.03	0.11	1.11	0	7.92	0.06	1.04	9.18	46.59
*Festuca fimbriata* American II	0.01	4.47	0.15	0.04	1.6	1.18	0.12	0.43	0	0.04	0	0.34	1.66	0.09	0.21	0	0	1.18	0	0.2	0	0.05	3.91	0	1.62	0.02	0	21.54	38.87
*Festuca francoi* Aulaxyper	0	0.13	0.02	0	1.17	0.16	0.01	0.02	0.09	0.85	0	0.07	1.62	0.23	0.03	0	0	0.81	0	0.02	0	0.27	4.16	0	1.07	0	0	26.83	37.56
*Festuca gracillima* American-Neozeylandic	0	4.5	0.15	0.02	1.74	0.61	0	0	0	0.87	0	1.22	6.05	1.54	0.01	0	0	0.76	0	0.92	0	0.61	1.45	0	8.23	0	6.04	13.95	48.68
*Festuca holubii* American I	0	10.87	0.07	0	6.86	0.76	0	0	0	0.61	0	0.02	3.52	1.1	0.01	0	0	4.54	0	0.54	0.01	0.42	5.96	0	0.9	0.01	0.99	13.32	50.52
*Festuca ovina* Festuca	0.03	0.26	0.02	0	3.16	0.33	0	0	0	7.18	0	0.59	4.26	1.04	2.04	0	0	2.01	0	0	0.01	0.28	5.22	0	2.75	0.03	7.1	12.28	48.58
*Festuca pampeana* American Pampas	0	0.52	0	0.06	0.63	0.1	0.06	0	0	0	0	0.33	6.47	0.03	0	0	0	0.85	0	0.19	0	1.02	4.77	0	2.85	0	0	23.59	41.48
*Festuca pirenaica* Exaratae	0	10.88	0.36	0	7.1	0.6	0.12	0	0.01	0.87	0	0.34	4.32	1.48	0.21	0	0	4.67	0	0.8	0	0.05	2.66	0	0	0	2.09	11.66	48.21
*Festuca procera* American II	0.01	4.47	0.11	0	3.53	0.36	0.03	0	0	1.31	0	0.26	3.46	1.41	0.42	0	0	2.62	0	0.01	0	0.37	5.94	0	7.32	0.04	0.96	10.43	43.08
*Festuca pyrogea* Festuca	0.04	0.02	0	0	0.08	0	0	0.03	0	0	0	0	6.91	0.02	0.49	0	0	0.95	0	0	0	0.42	13.4	0	1.24	0	0	24.13	47.73
*Festuca rubra* Aulaxyper	0	0.08	0	0	0.96	0.3	0.01	0	0	8.55	0	0.61	2.11	0.69	1.24	0	0	0.95	0	0.01	0	0.7	6.56	0	0.76	0	0.32	22.79	46.65
*Megalachne masafuerana* American Pampas	0	1.44	0	0.18	0.38	1.98	0	0.02	0	0.1	0	0	7.4	3.31	0	0	0	1.4	0.03	0	0	0.4	1.88	0	2.74	0.04	0.14	23.85	45.28
*Vulpia ciliata* Psilurus-Vulpia	0.14	3.81	0	0	0.78	0.49	0.16	0	0.01	0.22	0	0.08	16.79	0.86	0.56	0	0	1.6	0	0.11	0	0.7	2.76	0	8.45	0.33	2.33	11.74	51.91
**Mean±SD**	**0.02**	**5.94**	**0.05**	**0.07**	**2.26**	**0.35**	**0.03**	**0.02**	**0.00**	**2.86**	**0.00**	**0.79**	**7.68**	**1.42**	**3.31**	**0.00**	**0.00**	**1.84**	**0.00**	**0.21**	**0.05**	**0.53**	**3.41**	**0.06**	**4.43**	**0.03**	**0.89**	**15.61**	**51.85**
**Kruskal Wallis test**	**30.99**	**37.17**	19.01	**35.24**	21.49	21.30	19.04	20.78	9.81	19.63	12.89	20.52	**31.43**	**31.25**	**30.49**	8.40	14.67	**24.84**	22.50	**32.30**	**23.49**	**24.54**	**23.56**	14.67	**28.22**	14.33	22.30	21.92	
**Kruskal Wallis test *p.value***	**0.01**	**0.00**	0.16	**0.00**	0.09	0.09	0.16	0.11	0.78	0.14	0.53	0.11	**0.00**	**0.01**	**0.01**	0.87	0.40	**0.04**	0.07	**0.00**	**0.05**	**0.04**	**0.05**	0.40	**0.01**	0.43	0.07	0.08	

*Kruskal–Wallis tests for significant differences in repeat proportions for each repetitive element across the studied samples. Significant values are highlighted in bold.*

LTR-Gypsy Retand elements were the most represented repeats in almost all genomes, especially within the BL and Schedonorus groups, where they covered >10% and up to 20% of several Subbulbosae, Leucopoa, Central-South American, ‘European,’ *F.* gr. *arundinacea* and *Lolium* genomes, as well as two genomes of the BL and FL groups (*F. molokaiensis*, *V. ciliata*). Only the BL Tropical-South African and the FL American II and Aulaxyper genomes showed low coverages (<2%) of Retand repeats (Table 2 and Figure 1). The more heterogeneous LTR-Gypsy Tekay and Athila elements were also well represented in some genomes, the former in the BL genomes (*F. scabra* 14%, *F. mekiste* 11%) and the latter in the *Lolium* genomes (*L. perenne*, 25%; *L. rigidum* 23%). In contrast, those elements generally had low coverages (<2%) in FL genomes (Table 2 and Figure 1). Other LTR-Gypsy families were only moderately represented in some groups, such as Ogre in the Tropical and South African genomes (e.g., *F. mekiste*, 7.9%; *F. africana*, *F. scabra*, 4.6%) and *L. rigidum* (4.8%), and CRM in several Schedonorus genomes (e.g., *L. persicum* 5.1%, *F. pratensis* 4.3%) although they showed low coverages (<2%) in most of the remaining genomes. The LTR-Gypsy OTA, Reina and Tat families were only residually present in a few genomes (Table 2).

LTR-Copia Angela elements were the second most frequent repeat family in all Loliinae genomes. They were highly represented in the genomes of Central-South American taxa in both the BL (12–27%) and FL (9.8–10.8%) groups, relatively abundant in all remaining BL genomes (6.6–8.8%), moderately abundant in Schedonorus genomes (except the ‘Mahgrebian’ taxa, <2%) and in FL *F. eskia* and BL *F. molokaiensis* (5.7–7.2%), and poorly represented in the remaining FL genomes (<2%) (Table 2 and Figure 1). LTR-Copia SIRE elements showed moderate to low frequency in all genomes except in *F. molokaiensis* (10%) and FL Eskia, American I and American II genomes (5.4–7.5%). Other LTR-Copia families (Ale, Ikeros, Ivanna, TAR, Tork) were only residually represented in a few Loliinae genomes (Table 2 and Figure 1). TIR Class II transposons were found less frequently in Loliinae genomes; only CACTA elements were present in all taxa although they were only moderately represented in some FL American I, American II and Hawaiian genomes and in BL Subbulbosae and Leucopoa genomes (4–5.5%). Representation of other transposon elements (Mutator, Harbinger, hAT) in Loliinae genomes was only residual (Table 2 and Figure 1). Some of the less frequent Class I and Class II repetitive elements were only represented in a very small fraction of some particular genomes (e.g., Reina in *L. saxatile*; hAT in *M. masafuerana*; Tat in *F. simensis*; Table 2). Tandem satellite repeats were generally moderately to poorly represented in most Loliinae genomes, except for their relatively high representation in FL *F. procera* and *F. pyrogea* (13.3%) and Schedonorus *F. simensis* (12%) and its moderate representation in FL Exaratae, Festuca and Aulaxyper genomes (4.2–5.9%). Kruskal–Wallis rank tests performed for each of the Loliinae repeat elements found significant differences for Retand, CRM, Tekay, Angela, Ivanna, Ale, LTR, CACTA, Mutator, Harbinger, rDNA and satellite repeats when examined in the entire group of samples (Table 2).

**FIGURE 2:**
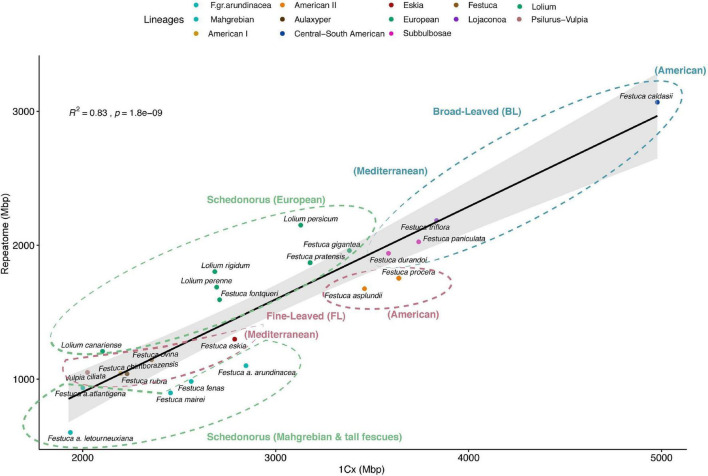
Correlation plot of repeat content and genome size variation (1Cx) for the 23 Loliinae taxa with known genome sizes. Summed abundance values of the most represented repeat types obtained from the individual RE2 analysis. Pearson correlation analysis (*R*^2^ = 0.83, *p* = 1.8e–09). Ellipses with dashed lines encircle the main Loliinae groups and subgroups [broad-leaved Loliinae (BL), blue; Schedonorus, green; fine-leaved Loliinae (FL), magenta]. Color codes of Loliinae lineages correspond to those indicated in Figure 1.

**FIGURE 3:**
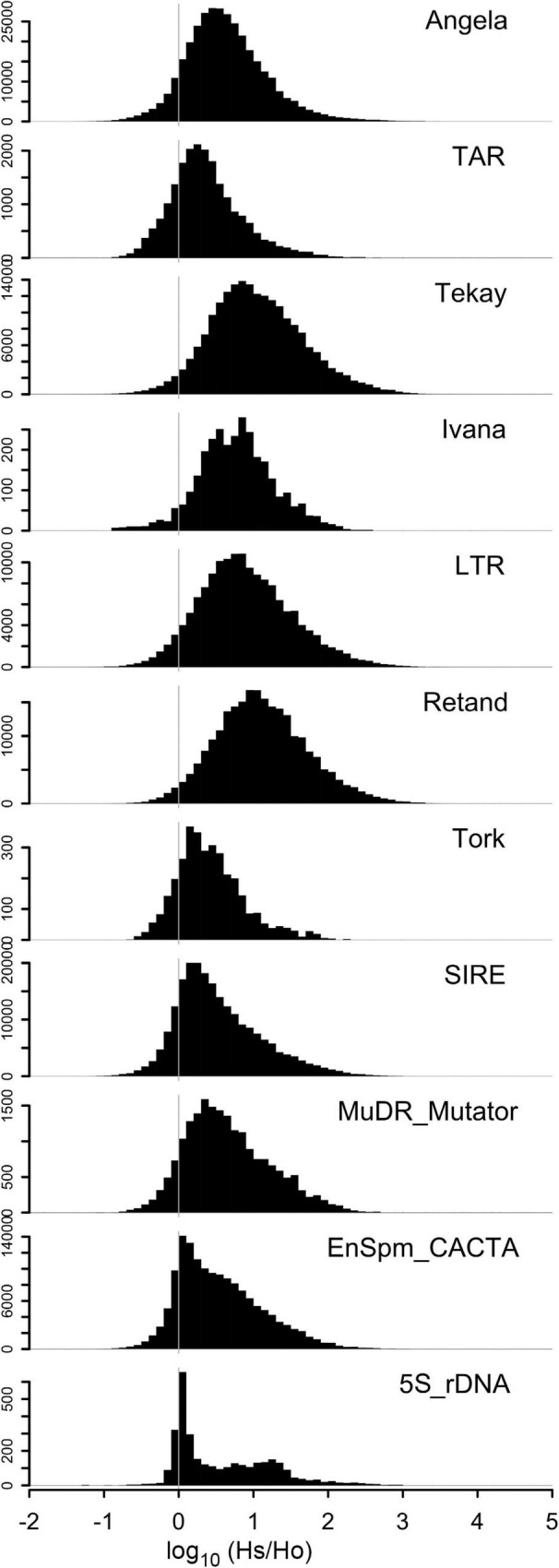
Global variability of main repeat types and their sequence conservation across the Loliinae genome landscapes. Histograms show distributions of read similarity Hs/Ho hit ratios [frequencies of read similarity hits to reads from the same species (Hs) or to reads from all other species (Ho) (log scale, *x*-axis) and number of reads (*y*-axis)]. Hs/Ho ratios close to one (0 on the logarithmic scale) indicate sequence conservatism while larger values indicate sequence diversification.

Regression model analysis of repeat content and monoploid genome sizes differences among the 23 Loliinae species with known 2C data, after PIC correction, showed a strong correlation when data from all main repeats were combined (*R*^2^ = 0.83, *p* = 1.8E-09), accounting for 65.2% differences in genome size between species (Table 3 and Figure 2). Angela repeats presented the highest correlation (*R*^2^ = 0.71, *p* = 5.44E-07), followed by TAR (*R*^2^ = 0.54, *p* = 5.85E-05), Tekay (*R*^2^ = 0.38, *p* = 0.0018), Ivanna (*R*^2^ = 0.35, *p* = 0.002), LTR (*R*^2^ = 0.27, *p* = 0.011) and Retand (*R*^2^ = 0.21, *p* = 0.02) repeats, while the other repetitive elements did not show significant correlations. The Angela family also showed the highest contribution to pairwise differences in genome sizes (19.6%), followed by Retand (10.7%), Tekay (6.47%) and LTR (5.49%), while the contributions of the other families were <5% (Table 3 and Supplementary Figure 2). Our genome landscape analysis of global variability of these individual repeat types across the Loliinae genomes showed different histogram profiles of Hs/Ho hit ratios (Figure 3). The histogram of control 5S rDNA sequences comprised a narrow major peak near zero on the log(Hs/Ho) *x*-axis, indicating that the ratios of intraspecific Hs to interspecific Ho hit frequencies were close to one, and thus reflected the high sequence conservation of the 5S genes. In contrast, this 5S rDNA histogram also included a wide right-hand tail of log(Hs/Ho) hit values ranging from 0.1 to 3, accounting for the high divergence of intergenic spacer sequences (IGS) of 5S rDNA. However, the histogram patterns of the ten repeats analyzed showed general Gaussian distributions for log(Hs/Ho) hit values (Figure 3). Among the repeats that contributed the most to genome size variation (Table 3 and Supplementary Figure 2), Angela elements generated main peaks of log(Hs/Ho) values closer to zero in the histogram than those of Retand, LTR and Tekay elements (Figure 3), suggesting a slightly higher conservatism of the Angela sequences and a higher diversification of the Retand, LTR and Tekay sequences in the Loliinae genome landscape.

**TABLE 3:** Pearson linear correlation of repeat abundance with genome size variation (1Cx) in Loliinae, after PIC correction, and contribution of individual repeats to the genome size differences between species.

Repeat type	Correlation to genome size		Abundance in the analyzed genomes [Mbp/1Cx]		Average contribution to pairwise differences in genome sizes [%]
	*R* ^2^	*p*-Value	Min	Max	
Angela	**0.71**	**5.44E-07**	1.775	1366.503	**19.6**
TAR	**0.54**	**5.85E-05**	1.172	24.058	**0.642**
Tekay	**0.38**	**0.00187**	0	364.516	**6.47**
Ivanna	**0.35**	**0.00281**	0	16.597	**0**
LTR	**0.27**	**0.0111**	0	480.094	**5.49**
Retand	**0.21**	**0.0265**	46.947	652.52	**10.7**
Tork	0.16	0.0566	0	5.454	0.0376
SIRE	0.14	0.0784	3.791	282.611	2.8
MuDR_Mutator	0.11	0.131	0	32.148	0.0986
EnSpm_CACTA	0.09	0.165	8.715	190.978	2.27
Ty1_Copia	0.08	0.18	0	2.514	0
Ty3_Gypsy	0.08	0.197	0	0.208	0
Mobile_element	0.06	0.257	0	103.646	0
Ikeros	0.05	0.285	0	17.96	0
LINE	0.05	0.314	0	6.74	0
OTA	0.03	0.397	0	0.379	0
Unclassified	0.03	0.438	197.426	611.73	4.08
CRM	0.03	0.443	8.348	161.049	0.751
Repeat	0.01	0.61	0	167.43	0
Ale	0.01	0.716	0	3.465	0
PIF_Harbinger.	0.01	0.737	0	5.893	0
rDNA_5S-45S	0.00	0.789	1.446	102.852	−0.152
Athila	0.00	0.852	1.146	680.565	0.778
Satellite	0.00	0.863	30.69	272.468	−0.0164
Ogre	0.00	0.93	0	130.467	0.183
**All repeats**	**0.83**	**1.8E-09**	591.539	3067.826	**65.2**

*Only the most represented repeat types of Loliinae are shown. Significant values are highlighted in bold.*

### Repeatome Phylogenies of Loliinae and Phylogenetic Signal of Repeats

**FIGURE 4:**
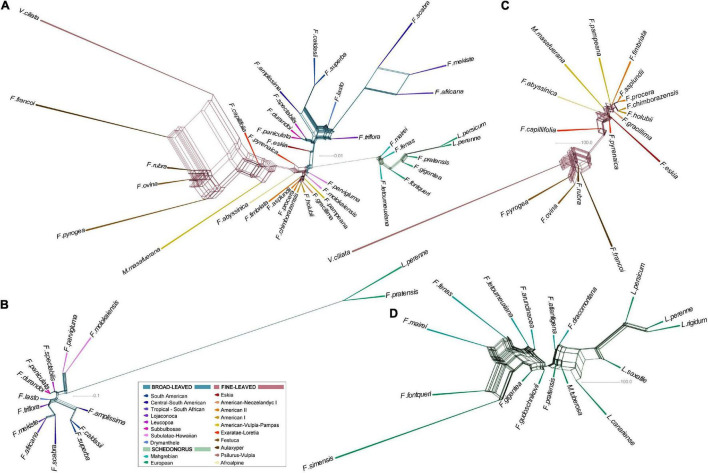
Evolutionary networks based on standardized repeat data sets obtained from the comparative RE2 analysis of the four Loliinae evolutionary groups: **(A)** Loliinae, **(B)** broad-leaved (BL) Loliinae, **(C)** fine-leaved (FL) Loliinae, and **(D)** Schedonorus. The networks were constructed from distance-based NJ trees computed with pairwise Euclidean distances between samples (see text). Color codes of Loliinae lineages are indicated in the respective charts. Scale bar: number of mutations per site.

The results of the RE2 comparative analysis of Loliinae repeats recovered different types and numbers of shared or sample specific repetitive elements in each of the four Loliinae evolutionary groups studied (Supplementary Table 3). RE2 annotated different numbers of tops clusters in each group [Loliinae: 337 clusters (total number of reads 2,659,145 (57%); minimum number of reads 468); FL: 308 (2,245,911 (57%); 395); BL: 336 (2,841,940 (64%); 443); Schedonorus: 270 (1,771,749 (65%); 274)] (Supplementary Tables 3A–D) representing presumably orthologous repeat families from different samples that were grouped together due to their high repeat sequence similarity (Macas et al., 2015). The number of top clusters used to build the NJ trees and networks was reduced in all groups after discarding clusters with NA or zero read values for some samples (Loliinae: 38 clusters; BL: 96; FL: 122; Schedonorus: 167) (Supplementary Tables 4A–D). Networks constructed from distance-based NJ trees computed with the Euclidean distances (Figures 4A–D) showed better resolutions than those obtained from NJ trees computed with the inverse distances (Supplementary Figures 3A–D); therefore, descriptions of repeatome phylogenies were based on the Euclidean networks. The unrooted Loliinae network showed three divergent groups corresponding to each of the main BL, FL and Schedonorus lineages (Figure 4A). In this network, the Schedonorus group was highly isolated from the others and, in contrast to its position in the Loliinae tree (Figure 1), it was closer to the FL group than to the BL group. Similarly, the fine-leaved *F. eskia* was closer to the BL group than to its own FL group. The unrooted BL network (Figure 4B) inferred a topology congruent with that of the BL lineage in the Loliinae tree except for the sister relationship of South African *F. scabra* with the other Tropical and South African taxa and the sister relationship of the two Subbulbosae species (*F. paniculata*/*F. durandoi*), resolutions that, however, matched those recovered from the 35S Loliinae tree (Supplementary Figure 1C). The unrooted FL network (Figure 4C) was generally consistent with the combined Loliinae tree except for the positions of the American I and American-Pampas taxa, which were closely related to the American II taxa; Afroalpine *F. abyssinica* was also close to them (Figure 4C). These phylogenetic topologies were also congruent with those retrieved in the 35S Loliinae tree (Supplementary Figure 1C).

The potential phylogenetic signal of the abundance of the repeat clusters (Supplementary Tables 4A–D) evaluated in different Loliinae subtrees, rendered significant *K* values for distinct clusters in each group (Supplementary Table 5 and Supplementary Figure 4). Within the Loliinae group, nine clusters (1 LTR, 4 Angela, 1 SIRE, 3 CACTA) had significant *K* values on the Loliinae tree cladogram, although only the *K* values of the four Angela clusters were >0.5. In contrast, within the FL group only four clusters (1 Angela, 2 Tekay, 1 repeat) had significant *K* values on the FL tree cladogram but all of them were ∼1. The BL and Schedonorus groups had 17 clusters that carried phylogenetic signal on their respective tree cladograms; however, whereas all the BL clusters (1 LTR, 3 Angela, 8 Tekay, 4 Athila, 1 Mutator) had *K* values close to 1, only nine out of the 17 Schedonorus clusters had *K* values ∼1 (3 LTR, 3 Repeat, 1 CRM, 1 Mutator, 1 Tekay) while the remaining eight cluster (6 LTR, 1 Athila, 1 Mutator) carried more phylogenetic signal than expected (*K* values > 1) (Supplementary Table 5 and Supplementary Figure 4).

### 5S rDNA Graph-Clusters of Loliinae

The Loliinae 5S rDNA region ranged from 245 to 316 bp in the Loliinae [a 120 bp 5S gene conserved in all taxa plus a variable IGS for specific taxa (range 125–196 bp); Supplementary Table 1]; the 5S MSA consisted of 316 bp (120 bp 5S gene; 196 bp IGS). The Loliinae 5S ML tree (Supplementary Figure 5) had poor support for most of its branches and was topologically incongruent with both the combined Loliinae tree (Figure 1 and Supplementary Figure 1B) and the separate plastome and nuclear 35S rDNA trees (Supplementary Figures 1C,D). The only supported lineage was the Schedonorus clade (Supplementary Figure 5) although its internal resolution also departed from those of the other trees and was not considered further.

**FIGURE 5:**
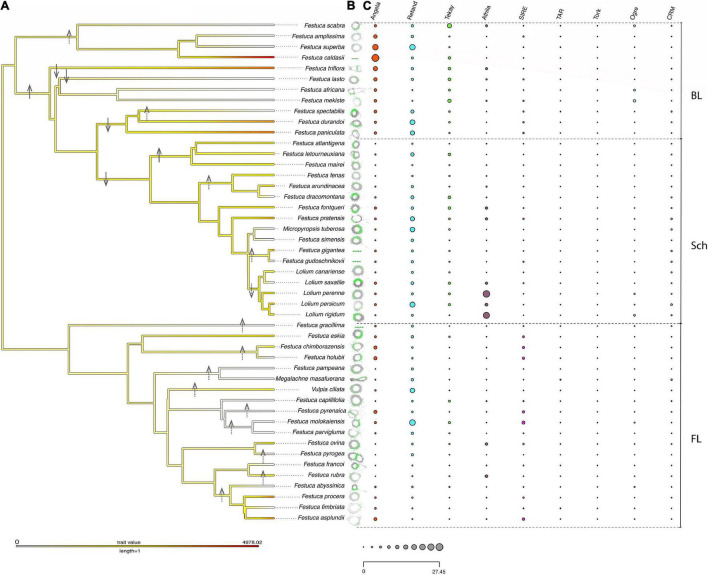
**(A)** Maximum Likelihood phylogenetic tree (combined plastome + nuclear 35S rDNA data) of the 47 studied Loliinae samples showing their genome sizes by the colors of the terminal branches (color gradients indicate inferred genome size changes); white, missing data. **(B)** 5S clustering graph plots generated by RE2. **(C)** Proportions of the most abundant repeat elements (standardized values) obtained from the individual RE2 analysis of repeats are shown for each taxon. Hypothesized scenarios of allopolyploidization and diploidization events mapped onto the tree branches (ancestral allopolyploidization: solid arrow up; ancestral diploidization: solid arrow down; recent allopolyploidization: dashed arrow up; recent diploidization: dashed arrow down). BL, broad-leaved Loliinae; Sch, Schedonorus; FL, fine-leaved Loliinae.

Analysis of the 5S rDNA clusters of 47 Loliinae species studied produced different types of simple and complex graphs that did not always match the expected shapes for their respective ploidy levels (Table 4 and Figure 5). As expected, most graph topologies of diploid taxa corresponded to a simple circular graph that likely represents a single 5S gene family and locus. This was observed for most FL (*F. eskia, F. capillifolia, F. ovina*) and Schedonorus (*F. pratensis, F. fontqueri*, *M. tuberosa*, all five *Lolium* species) diploids. However, within the BL diploids one species showed a simple graph (*F. lasto*) but two species (*F. triflora, F. paniculata*) had complex graphs with two IGS loops interconnected by a junction section (coding region of the 5S gene), suggesting that the latter species could have two 5S ribotypes (Figure 5). Within Loliinae polyploids, 5S graph topologies ranged from those taxa showing complex graphs with a number of loops corresponding to their assumed number of 5S loci (tetraploid *F. pyrenaica*, two loops), to high polyploids with lower number of loops than expected based on their ploidy levels (decaploids *F. africana* and *F. letourneuxiana*, two loops), and low-to-high polyploids showing a simple graph (tetraploids *V. ciliata*, *F. parvigluma, F. procera, F. abyssinica, F. simensis, F. fenax, F. mairei, F. mekiste*; hexaploids *F. rubra, F. chimborazensis, F. asplundii, F. fimbriata, F. amplissima*; octoploids *F. pampeana, F. spectabilis, F. atlantigena, F. superba*). Loliinae species from the southern hemisphere with unknown ploidy level displaying complex 5S graphs (e.g., *F. pyrogea*, *M. masafuerana*; two loops) were identified as polyploids, while those displaying a single graph (e.g., *F. dracomontana, F. holubii, F. molokaiensis*) could not be classified as such (Figure 5).

**TABLE 4:** Ploidy levels and genomic pair-end read features of 5S rDNA loci and cluster graph parameters of the studied Loliinae taxa.

Taxon	Ploidy level	N. reads in cluster	Genome proportion (%)	Repeat size (bp)	k-mer coverage	Connected component index	Graph shape (type)
*Festuca abyssinica*	4x	180	0.036	316	0.885	0.967	1
*Festuca africana*	10x	214	0.043	317	0.488	0.879	2
*Festuca amplissima*	6x	158	0.032	318	0.744	0.994	1
*Festuca a. arundinacea*	6x	369	0.074	315	0.78	0.987	1
*Festuca a*. *letourneuxiana*	10x	532	0.11	307	0.721	0.974	2
*Festuca a. atlantigena*	8x	428	0.086	307	0.791	0.981	2
*Festuca asplundii*	6x	110	0.022	318	0.894	0.982	1
*Festuca caldasii*	4x	–	–	–	–	–	–
*Festuca capillifolia*	2x	340	0.068	318	0.9	0.976	1
*Festuca chimborazensis*	6x	179	0.036	319	0.845	0.899	2
*Festuca dracomontana*	–	629	0.13	307	0.75	0.936	1
*Festuca durandoi*	2x	520	0.1	318	0.812	0.994	2
*Festuca eskia*	2x	525	0.1	319	0.873	0.989	2
*Festuca fenas*	4x	222	0.044	307	0.781	0.973	1
*Festuca fimbriata*	6x	104	0.021	317	0.8	0.923	1
*Festuca fontqueri*	2x	470	0.094	296	0.824	0.977	2
*Festuca francoi*	2x	632	0.13	317	0.748	0.981	2
*Festuca gigantea*	6x	–	–	–	–	–	–
*Festuca gracillima*	6x	–	–	–	–	–	–
*Festuca gudoschnikovii*	4x	–	–	–	–	–	–
*Festuca holubii*	–	179	0.036	318	0.863	0.944	2
*Festuca lasto*	2x	470	0.094	296	0.824	0.977	1
*Festuca mairei*	4x	330	0.066	315	0.791	0.921	1
*Festuca mekiste*	–	109	0.022	317	0.619	0.917	1
*Festuca molokaiensis*	–	208	0.042	316	0.666	0.861	2
*Festuca ovina*	2x	331	0.066	316	0.952	0.985	1
*Festuca pampeana*	8x	402	0.08	317	0.812	0.98	1
*Festuca paniculata*	2x	269	0.054	318	0.781	0.978	2
*Festuca parvigluma*	4x	190	0.038	316	0.711	0.884	1
*Festuca pratensis*	2x	447	0.089	545	0.832	0.911	2
*Festuca procera*	4x	165	0.033	317	0.863	0.976	2
*Festuca pyrenaica*	4x	204	0.041	316	0.62	0.941	2
*Festuca pyrogea*	–	850	0.17	326	0.602	0.955	2
*Festuca rubra*	6x	338	0.068	316	0.737	0.87	2
*Festuca scabra*	4x	232	0.046	301	0.782	0.978	2
*Festuca simensis*	4x	412	0.082	296	0.675	0.951	2
*Festuca spectabilis*	6x	1128	0.23	316	0.791	0.99	2
*Festuca superba*	8x	184	0.037	316	0.772	0.995	1
*Festuca triflora*	2x	217	0.043	262	0.498	0.982	2
*Lolium canariense*	2x	306	0.061	294	0.842	0.974	1
*Lolium perenne*	2x	447	0.089	307	0.868	0.982	1
*Lolium persicum*	2x	1154	0.23	307	0.832	0.976	1
*Lolium rigidum*	2x	892	0.18	307	0.809	0.983	1
*Lolium saxatile*	2x	157	0.031	308	0.914	0.975	2
*Megalachne masafuerana*	–	690	0.14	224	0.438	0.997	2
*Micropyropsis tuberosa*	2x	911	0.18	307	0.865	0.98	1
*Vulpia ciliata*	4x	414	0.083	315	0.916	0.993	2

*Graph shape types (type 1, simple circular-shaped graph with one loop; type 2, complex graph with two loops where the interconnected loops represent IGS spacers). 5S clustering analysis of F. caldasii, F. gigantea, F. gracillima and F. gudoschnikovii could not be performed due to insufficient number of 5S reads in the clusters. Hyphens, missing data.*

## Discussion

### Characterization of the Loliinae Repeatome and Its Impact on the Diversification of the Genome Size of Its Lineages

Our large-scale exploratory analysis of the Loliinae repeatome has uncovered the abundance and composition of the repetitive DNA across the genome landscape of all the subtribal lineages, confirming the substantial contribution of the repeatome to the genome size diversification of the studied Loliinae genomes (Table 2, Figure 1, and Supplementary Figure 1E). The repetitive elements represent more than half of the holoploid genome of most surveyed Loliinae taxa and accounted for the largest percentages (>60%) in the BL and *Lolium* genomes (Table 2, Figure 1, and Supplementary Figure 1E). Our data has demonstrated that the 1.5- to 3-fold downsizing monoploid genome trend observed by previous authors between BL and FL Loliinae lineages (Catalán, 2006; Šmarda et al., 2008) can be attributed to proportional amounts of their respective repetitive elements (Tables 2, 3, Figure 1, and Supplementary Figure 1E). Unlike other studies that found no evidence of repeat activity causing large variation in genome size among diploid species (e.g., *Anacyclus*; Vitales et al., 2020a), our analyses have corroborated that striking differences in the 1.5-fold increase in genome size between BL and FL Loliinae diploid genomes was caused by significant differences in the repeat contents of the more abundant Retand and Angela retrotransposons (Tables 2, 3, Figures 1, 2, and Supplementary Figure 2). In general, the Loliinae diploid genomes, -either BL, FL or Schedonorus-, showed higher proportions of repeats than the allopolyploid genomes except for some of the South American BL and FL polyploid genomes (Tables 1, 2, Figure 1, and Supplementary Table 1). Thus, our data partially rejects the “polyploid genome shock” hypothesis that predicts increased genome sizes (and correlated repeat expansions) in polyploids, as well as the additive pattern of diploid repeat contents in the derived allopolyploids (e.g., *Melampodium*; McCann et al., 2018). In contrast, it supports the alternative hypothesis that predicts a trend for genome (and repeatome) reduction after polyploidization due to genomic losses of duplicated genome fragments (e.g., *Spartina* and several sequenced plants; Chen, 2007; Parisod et al., 2010; Michael, 2014). The significantly lower genome sizes and correlated lower repeat contents of Old World Loliinae polyploids relative to diploids (Tables 1, 2, Figures 1, 2, and Supplementary Figure 2) could be attributed to the relatively ancestral DNA ages of some of these polyploid lineages [e.g., Schedonorus Mahgrebian (6.3 Ma) and FL Aulaxyper (6.1 Ma) clades; Moreno-Aguilar et al., 2020], which might have eliminated duplicated repeats over time. Furthermore, the high level of ploidy (6x-8x-10x) of these allopolyploids, which have apparently lost more redundant repeats compared to their closely related diploids or lower polyploids, could have resulted from a selective process to limit repetitive DNA damaging activity (Wang et al., 2021). Alternatively, some of these high polyploids could have originated through autopolyploidy or a combination of autopolyploidy and allopolyploidy; those scenarios would better explain the simple 5S graph patterns observed in many of these taxa (Figure 5). However, all thoroughly investigated Loliinae polyploids have been shown to be allopolyploids (Catalán, 2006, and references therein). The considerable reductions in retrotransposon and transposon contents detected in high polyploid Loliinae species are consistent with parallel losses of 35S rDNA loci in the same taxa (e.g., BL *F. africana*-10x, Namaganda, 2007; Schedonorus *F. atlantigena*-8x and *F. letourneuxiana*-10x, Ezquerro-López et al., 2017), suggesting that the two types of repetitive DNA reductions might have occurred after large genomic rearrangements in these high polyploids. In contrast, the large repeat contents of some Old World Loliinae diploids could be explained by the dynamic activity of young repeat types that have proliferated in recent diploid lineages (e.g., Athila in *Lolium*; Table 2 and Figures 1, 2; Zwyrtková et al., 2020).

As in many angiosperms (Eickbush and Malik, 2002), the retrotransposons LTR-Gypsy Retand (1.6–21.3%) and LTR-copia Angela (0.02–27.5%) were the most widely represented repeat family in the Loliinae genomes (Table 2 and Figure 1). The Tekay, Athila and SIRE elements followed, while other retrotransposons (Ogre, CRM) and transposons (CACTA) were less common (Table 2 and Figure 1). Together, they showed a strong correlation with genome size (*R*^2^ = 0.83, *p* = 1.8E-09) and a considerable contribution to the differences in genome sizes (65.2%) between Loliinae lineages (Table 3 and Figure 2), although these contributions varied for the most abundant types. The Retand repeats contributed significantly to the larger genome sizes of the BL and Schedonorus genomes compared to the FL genomes (Table 2), while the Angela repeats also contributed to the large sizes of the BL genomes and, notably, to some relatively large genomes of FL American I and American II genomes (Table 2). The Angela elements showed the highest correlation of repeat content with genome size (*R*^2^ = 0.71) and also explained the greatest differences in genome size between species (19.6%), in contrast to the Retand repeats that presented lower correlation and contribution values (*R*^2^ = 0.21; 10.7%) (Table 3 and Supplementary Figure 2). The important role of Angela retrotransposons in genome size diversification of Loliinae genomes is likely related to the relatively higher conservatism of these repeats, compared to the more variable behavior of Retand and other repeat elements (Figure 3). In agreement with other studies that have also detected older and less active Angela copies in Fabaceae (Macas et al., 2015) and Triticeae (Wicker et al., 2017, 2018), but in contrast to the finding of a high turnover of Angela families in *Brachypodium distachyon* (Stritt et al., 2020), our data indicated that Angela repeats also tend to be relatively conserved in Loliinae and have probably better fitted long-term genomic diversification trends of their ancestral genomes (19.4 Ma; Moreno-Aguilar et al., 2020). In contrast, young and highly heterogeneous Athila families likely experienced a recent burst within the *Lolium* clade and especially in the allogamous *L. perenne* and *L. rigidum* genomes (23–25%) and were moderately abundant in other studied ray-grasses and their close *F. pratensis* and *F. fontqueri* relatives (7–8%) (Table 2 and Figure 1). Noticeably, Athila elements also proliferated in recent FL *F. rubra* (8.5%) and *F. ovina* (7.1%) genomes, constituting the best represented annotated family in the red and sheep fescues (Table 2 and Figure 1).

### Phylogenetic Value of the Loliinae Repeatome and Deconvolution of the Origins of Some Genomes From 5S Cluster Graphs

In agreement with previous studies from other angiosperms (Dodsworth et al., 2015; McCann et al., 2018, 2020; Vitales et al., 2020b; Herklotz et al., 2021), the different amounts of shared repeats retrieved from comparative RE2 analyses of Loliinae have been shown to contain phylogenetic information at different systematic levels across the four Loliinae evolutionary groups. All evolutionary analyses have confirmed their ability to recover deep-to-shallow evolutionary relationships that were highly or relatively consistent with those based on the 35S rDNA and the plastome and combined data sets, respectively (Tables 1, 4, Figures 4, 5, Supplementary Tables 3, 4, and Supplementary Figures 1, 3). Some of the networks have, however, uncovered repeatome-specific topological features, which were not observed in the MSA trees (Figure 4).

The unrooted Loliinae and BL repeatome networks have demonstrated the high isolation of Schedonorus from the remaining Loliinae lineages (Figures 4A,C). This large divergence was based on the uniqueness of the Schedonorus repeat amounts within the representatives of the subtribe (Supplementary Table 3). Although Schedonorus has traditionally been considered a recent split within the broad-leaved Loliinae in all previous evolutionary studies (Minaya et al., 2017; Moreno-Aguilar et al., 2020, and references therein), and in the current combined tree of Loliinae (Figure 1 and Supplementary Figure 1B), this position is mostly based in the strong plastome topology (Supplementary Figure 1C) and its large sequence dataset. By contrast, the weak nuclear 35S ML topology showed extremely low support for the potentially basal paraphyletic divergences of the BL lineages and an unclear position for Schedonorus within them (Supplementary Figure 1D). The repeatome network placed Schedonorus more closely related to the FL than to the BL group (Figure 4A). More reliable phylogenies based on single-copy nuclear genes would be needed to decipher the evolution of Schedonorus and other Loliinae nuclear genomes. Here, the phylogeny of tall fescues and ray-grasses has been enriched with three new taxa, showing the sister relationships of the eastern Canary Islands endemic *Lolium saxatile*-2x (Scholz and Scholz, 2005) to *L. canariense*-2x, of Siberian *F. gudoschnikovii*-4x (Stepanov, 2015; Probatova et al., 2017) to its morphologically close Eurosiberian relative *F. gigantea*-6x, and of previously unstudied South African *F. dracomontana* (Linder, 1986) to *F. arundinacea*-6x (plastome tree) or to the ‘European’ clade (35S tree) (Figure 1 and Supplementary Figures 1A–D). A notable geographical signal of the repeatome was observed in the close relationships of NW African *F. fontqueri*-2x and Tropical African *F. simensis*-4x with Mahgrebian *F. mairei*-4x (Figure 4D), in contrast to their nesting positions within the predominantly diploid “European” clade in the plastome, 35S and combined trees (Supplementary Figures 1B–D). Also, the position of *F. dracomontana* in the repeatome network suggest that this austral Schedonorus species could be a polyploid close to the tall fescues (Figure 4D and Supplementary Figures 1B–D).

Geographically based evolutionary patterns of repetitive elements, congruent with those of the nuclear 35S rDNA tree, have been also observed in the FL and BL repeatome networks (Figures 4B,C and Supplementary Figure 1D). Within the FL network group, South American representatives of the American I, American-Pampas and American II lineages are closely related to each other (Figure 4B and Supplementary Figure 1D), while interspersed with other FL lineages in the plastome and combined Loliinae trees (Supplementary Figures 1B,C). These lineages are characterized by similar levels of Angela, Retand and LTR repeats (Table 2 and Figure 1) and were inferred to be of similar age (late Miocene_Pliocene transition, 3.4–5.4 Ma; Minaya et al., 2017). They are probably the descendants of the same paternal lineage, which probably evolved *in situ* but crossed with distinct maternal FL lineages giving rise to these close but separate allopolyploid clades (Supplementary Figures 1B,C). Within the BL group, the close relationships between South African *F. scabra* and Tropical and South African *F. africana*/*F. mekiste* and between Mediterranean-European *F. spectabilis* (Leucopoa) and *F. paniculata*/*F. durandoi* (Subbulbosae) based on shared repeat contents are more similar to those recovered in the 35S tree than in the plastome tree (Figure 4C and Supplementary Figure 1A–C), also suggesting a concerted evolution of nuclear repetitive DNA families and different hybridizations or chloroplast capture events with other BL lineages. In contrast, the close relationship of Central-American *F. amplissima* to the South American *F. superba*/*F. caldasii* lineage shown in the repeatome network is more similar to that observed in the plastome and combined Loliinae trees than in the 35S tree, probably due to the lower resolution of the nuclear topology (Figure 4C and Supplementary Figures 1A–C). Interestingly, these Central and South American taxa show some of the highest Loliinae genomic repeat contents (Tables 1, 2, Figure 1, and Supplementary Figure 1E) despite their high 6x-8x ploidy-levels. It could be a consequence of their relatively young ages (∼5 Ma; Moreno-Aguilar et al., 2020) and the lack of a time course to purge the excess of repetitive DNA (Michael, 2014), or a recent bloating of repeats. The phylogenetic value of the Loliinae repetitive elements has been further corroborated by the significant phylogenetic signals carried by different repeat clusters when tested on the respective tree cladograms of each of the four Loliinae groups (Supplementary Table 5 and Supplementary Figure 4). In most of the groups, the conservative Angela clusters had significant *K* values above 0.5 and close to 1, indicating their strong phylogenetic signal at different taxonomic levels.

Although tandem-repeated 5S rDNA did not retrieve a congruent evolutionary history for Loliinae (Supplementary Figure 5), their cluster graph topologies revealed their presumable number of loci (Figure 5), indicative of their potential hybridization events (Vozárová et al., 2021) and ploidy levels (Garcia et al., 2020). In contrast to the instability of 35S rDNA loci, the maintenance of 5S rDNA loci in high allopolyploid Loliinae species (Ezquerro-López et al., 2017) is consistent with their conserved patterns in other angiosperm allopolyploids (Garcia et al., 2017). Studies of allopolyploids with known subgenomes have demonstrated that species showing complex graphs with two IGS loops correspond to allotetraploids and those showing three loops to allohexaploids (Garcia et al., 2020), while in highly hybridogenous diploid rose species graphs with two loops probably correspond to ancient 5S rDNA families (Vozárová et al., 2021). Within the Loliinae studied, several polyploid taxa displayed 5S graphs with fewer loops than expected for their ploidy level (Figure 5), suggesting the existence of convergent evolution to one or few ribotypes. In contrast, three diploid species, BL *F. triflora* and *F. paniculata* and FL *F. francoi*, showed a 5S graph pattern typical of allotetraploids (Figure 5), supporting the hypothesis of their putative paleo-polyploid hybrid origin.

### Recurrent Rounds of Allopolyploidizations and Diploidizations Within Loliinae Lineages Revealed by Their Repeats

The widely accepted evolutionary scenario for the origin of the angiosperms, consisting of several rounds of hybridizations and allopolyploidizations followed by a return to the diploid state (Soltis et al., 2016) has been also inferred for the grasses and their main lineages. Evidence suggests that protograss whole genome duplication (WGD) was likely followed by later diploidizations that ended in current paleo-ancestral diploid karyotypes for temperate and tropical grasses (Salse et al., 2008). These involved distinct and profound genomic rearrangements, such as nested chromosome fusions, chromosome inversions and paleocentromere inactivation, along with differential losses of heterologous duplicated copies in subgenomes of divergent lineages (Murat et al., 2010). In contrast, new allopolyploidization events apparently led to the emergence of grass mesopolyploids, originated some million years ago, and grass neopolyploids, considered to have emerged during or after the Quaternary glaciations (Stebbins, 1985; Marcussen et al., 2014). Our data allow us to hypothetize that the evolution of Loliinae could have resulted from relatively rapid recurrent rounds of allopolyploidizations and diploidizations during the last 19–22 Ma (Minaya et al., 2017; Moreno-Aguilar et al., 2020) that have leaved their signatures on their repeats (Figure 1 and Supplementary Figure 1E) and 5S graph topologies (Figure 5). We postulate that the large genomes of the early diverging BL diploids (Lojaconoa, Drymanthele, Subulbosae; 7.5–5 Ma, Minaya et al., 2017) likely resulted from WGD of ancestral interspecific hybrids that later reverted to the diploid state with large chromosomes (Catalán, 2006), relatively large monoploid genome sizes and repeat contents (Table 2, Figures 1, 2, and Supplementary Figure 1E) and complex 5S graphs indicative of putative allotetraploids (Figure 5). This polyploid hybrid origin could also explain the potential heterosis of these robust broad-leaved fescues (Catalán, 2006). We also hypothetize that the large genomes and repeatomes of the basal BL polyploid lineages (Central-South American, South African) may have resulted from more recent allopolyploidizations (5–2.5 Ma, Minaya et al., 2017), with genomes that still maintain large sizes and proportions of repeats, and retain traces of more than one 5S ribotype (Table 2, Figures 1, 2, 5 and Supplementary Figure 1E).

Our findings are not fully compatible with the hypotheses of drastic genome contractions from a hypothetical large-genome Loliinae ancestor to the FL Loliinae lineage and in allopolyploids with large progenitor genomes but not in autopolyploids with small progenitor genomes (Loureiro et al., 2007; Šmarda et al., 2008). The observed reduction in repeat content and correlated genome size from the large BL Loliinae, through intermediate Schedonodorus and *F. eskia*, to the small FL Loliinae genomes (Figures 2, 5) could have resulted from independent genome size diversifications along the major Loliinae lineages (Figures 1, 5 and Supplementary Figure 1). Our data also support an alternative scenario of independent hybridization and polyploidization events across FL Loliinae, which are similar in age (∼16 Ma, Minaya et al., 2017) to BL Loliinae. Their small chromosomes and genome sizes (Catalán, 2006), especially for the taxa of the core Eurasian and Mediterranean Vulpia, Festuca and Aulaxyper (plus Exaratae) lineages (Tables 1, 2 and Figures 1, 2, 5), are similar to those of the close subtribes Parapholiinae, Cynosuriinae, and Dactylidiinae with which they also share 35S rDNA families (Catalán et al., 2004). Therefore, it could be hypothesized that the ancestor of these FL Loliinae did not undergo the same double genome enlargement as the ancestor of BL Loliinae. In addition, the various polyploid New World FL lineages (American I, American-Pampas, Subulatae-Hawaiian, American II), which show larger genome sizes and geographically structured repeat contents (Tables 1, 2, Figures 1, 4A,C, 5) are probably the results of recent allopolyploidizations (5–2.5 Ma, Minaya et al., 2017) that have not yet experienced considerable purging in their repeats.

The isolated Schedonorus lineage emerges as a highly dynamic repeat-driven evolving group, also accumulating evidence of various allopolyploidizations and diploidizations. A distinctive feature is the bloating of Athila repeats in the recently evolved diploid clade *Lolium*, especially in allogamous ray-grasses (Table 2, Figures 1, 2, and Supplementary Figure 2; Zwyrtková et al., 2020). In contrast, the Mahgrebian clade constitute a relatively ancestral lineage with unknown diploid relatives (Inda et al., 2014), although it shows signatures of ancient hybridizations in its 5S graph topologies (Figure 5). The Schedonorus Mahgrebian and the FL Aulaxyper allopolyploid lineages have experienced the most pronounced reductions in their repeats and genome sizes of all Loliinae studied (Table 2 and Figures 1, 2, 5). Interestingly, these two lineages also exhibit the highest and most extensive hybridization rates among the Loliinae, producing both intra- and intergeneric hybrids (Catalán, 2006). Schedonorus *Festuca* taxa spontaneously hybridize with each other and with close species of *Lolium* (x *Festulolium*) while Aulaxyper *Festuca* taxa (*F*. gr. *rubra*) also interbreed with each other and with close species of *Vulpia* (x *Festulpia*) (Catalán, 2006, and references therein). Therefore, it might be plausible that these two highly hybridogenous allopolyploid lineages have undergone large genome reshufflings to accommodate their highly divergent heterologous subgenomes and avoid DNA damage (Michael, 2014; Wang et al., 2021). These genomic rearrangements would have caused more severe losses in their respective repeats and genome sizes than those of other high polyploid American BL and FL Loliinae of similar ancestry that resulted from crosses of genomically similar progenitor species and presumably did not experience large repeat contractions (Table 2 and Figures 1, 2, 5).

## Data Availability Statement

The newly studied grass plastome and 35S and 5S rDNA cistron sequences have been deposited in the Genbank data base under accession numbers SAMN27777779–SAMN27777788, ON243855–ON243864 and ON248974–ON249019, and at the Github repository (https://github.com/Bioflora/Loliinae_Repeatome).

## Author Contributions

PC designed the study. MM-A, IA, LI, and PC collected the samples. MM-A and LI developed the experimental work. PC, MM-A, LI, IA, and PC analyzed the data and interpreted the results. PC and MM-A prepared the manuscript. PC, MM-A, LI, IA, and AS-R revised the manuscript. All authors contributed to the article and approved the submitted version.

## Conflict of Interest

The authors declare that the research was conducted in the absence of any commercial or financial relationships that could be construed as a potential conflict of interest.

## Publisher’s Note

All claims expressed in this article are solely those of the authors and do not necessarily represent those of their affiliated organizations, or those of the publisher, the editors and the reviewers. Any product that may be evaluated in this article, or claim that may be made by its manufacturer, is not guaranteed or endorsed by the publisher.

## Footnotes

1
http://www.plantsoftheworldonline.org/taxon/urn:lsid:ipni.org:names:328907-2

2
https://repeatexplorer-elixir.cerit-sc.cz

## Supplementary Material

Supplementary Table 1 Taxa included in the repeatome analysis of Loliinae. Taxonomic rank, taxon authorship, detailed localities and vouchers, and source of cytogenetic and genomic data. Group: BL, broad-leaved Loliinae; FL, fine-leaved Loliinae; Sch, Schedonorus. Chromosome number (2n), ploidy, genome size (2C, pg), monoploid genome size (1Cx, pg; 1Cx, Mbp) and GenBank accession codes for plastome and nuclear ribosomal 35S and 5S genes are given for each sample. Values in bold correspond to new data generated in this study. Outgroups used in the phylogenomic analyses: *Oryza sativa, Brachypodium distachyon*.

Supplementary Table 2 Loliinae samples used in the repetitive DNA analysis. Genome skimming paired-end (PE) reads per sample and PE reads selected by Repeat Explorer 2 per sample in each of the comparative analyses of the four Loliinae groups: Loliinae, BL (broad-leaved Loliinae), FL (fine-leaved Loliinae), Schedonorus.

Supplementary Table 3 Repeat Explorer 2 comparative analysis. Repeat content data for top clusters (repeat families) in each of the four evolutionary groups of Loliinae: **(A)** Loliinae; **(B)** broad-leaved (BL) Loliinae; **(C)** fine-leaved (FL) Loliinae; **(D)** Schedonorus.

Supplementary Table 4 Repeat Explorer 2 comparative analysis. Repeat content data for phylogenetically analyzed clusters (repeat families) in each of the four evolutionary groups of Loliinae: **(A)** Loliinae; **(B)** broad-leaved (BL) Loliinae; **(C)** fine-leaved (FL) Loliinae; **(D)** Schedonorus.

Supplementary Table 5 Phylogenetic signal based on Blomberg’s *K* values of repeat cluster contents obtained from the comparative RE2 analysis of Loliinae samples assessed in each of the four Loliinae groups: **(A)** Loliinae (38 samples, 38 clusters), **(B)** Broad-leaved (BL) Loliinae (15 samples, 96 clusters), **(C)** fine-leaved (FL) Loliinae (17 samples, 122 clusters), **(D)** Schedonorus (16 samples, 167 clusters), using the *phylosig* option of the *phytools* R package. Cluster abundance values (number of PE reads) are indicated in Supplementary Table 4. *K* values close to one indicate phylogenetic signal, values close to zero phylogenetic independence, and values >1 more phylogenetic signal than expected. *p*-Values based on 1000 randomizations. Significant values are highlighted in bold.

Supplementary Figure 1 **(A)** Combined (plastome + 35S rDNA) Loliinae coalescent species tree computed through Singular Value Decomposition quartets (SVDq) analysis showing bootstrap support values on branches. **(B–D)** Maximum Likelihood phylogenomic trees of 47 Loliinae samples based on **(B)** Combined (plastome + 35S rDNA) data, **(C)** plastome data, **(D)** nuclear 35S rDNA data, **(E)** Histograms of repeat contents per holoploid genome (1C) retrieved from the individual Repeat Explorer 2 analyses of the studied Loliinae samples mapped onto the Maximum Likelihood combined phylogenomic tree (plastome + nuclear 35S rDNA cistron) of Loliinae. Ultrafast bootstrap support values are indicated on branches. *Oryza sativa* and *Brachypodium distachyon* outgroups were used to root the trees. Color codes of Loliinae lineages are indicated in the charts. Scale bar: number of mutations per site.

Supplementary Figure 2 Correlation plots of repeat content and genome size variation (1Cx) for the 23 Loliinae taxa with known genome sizes. Individual plots for the most represented repeat types found across the 23 Loliinae taxa with known genome size data (see Table 2 and Figure 2). Color codes of Loliinae lineages correspond to those indicated in Figure 1.

Supplementary Figure 3 Evolutionary networks based on standardized repeat data sets obtained from the comparative RE2 analysis of the four Loliinae evolutionary groups: **(A)** Loliinae, **(B)** broad-leaved (BL) Loliinae, **(C)** fine-leaved (FL) Loliinae, **(D)** Schedonorus. The networks were constructed from distance-based NJ trees computed with pairwise inverse distances between samples (see text). Color codes of Loliinae lineages are indicated in the respective charts. Scale bar: number of mutations per site.

Supplementary Figure 4 Maximum Likelihood Loliinae tree cladograms (combined plastome + nuclear 35S rDNA cistron) showing the relationships among the studied samples in each of the four evolutionary groups of Loliinae and phyloheatmaps of normalized values for different sets of repeat clusters retrieved by RE2 from the comparative analysis of each group: **(A)** Loliinae (38 samples, 38 clusters), **(B)** broad-leaved (BL) Loliinae (15 samples, 96 clusters), **(C)** fine-leaved (FL) Loliinae (17 samples, 122 clusters), **(D)** Schedonorus (16 samples, 167 clusters). Repeat clusters showing significant phylogenetic signal are highlighted with dotted lines.

Supplementary Figure 5 Maximum Likelihood nuclear 5S rDNA cistron tree showing the relationships among the 47 studied Loliinae samples. Ultrafast bootstrap support values are indicated on branches. *Oryza eichingeri* and *Brachypodium distachyon* outgroups were used to root the tree. Color codes of Loliinae lineages are indicated in the chart. Scale bar: number of mutations per site.
