# Supplementary material for: Evolutionary Dynamics of the Repeatome Explains Contrasting Differences in Genome Sizes and Hybrid and Polyploid Origins of Grass Loliinae Lineages
Source: Front Plant Sci. 2022 Jul 1;13:901733. doi: 10.3389/fpls.2022.901733 (PMC9284676; doi:10.3389/fpls.2022.901733)
Supplement: Supplementary Table 1 — Taxa included in the repeatome analysis of Loliinae. Taxonomic rank, taxon authorship, detailed localities and vouchers, and source of cytogenetic and genomic data. Group: BL, broad-leaved Loliinae; FL, fine-leaved Loliinae; Sch, Schedonorus. Chromosome number (2n), ploidy, genome size (2C, pg), monoploid genome size (1Cx, pg; 1Cx, Mbp) and GenBank accession codes for plastome and nuclear ribosomal 35S and 5S genes are given for each sample. Values in bold correspond to new data generated in this study. Outgroups used in the phylogenomic analyses: Oryza sativa, Brachypodium distachyon. [file Table_1.DOCX]

**Table S1**. Taxa included in the repeatome analysis of Loliinae. Taxonomic rank, taxon authorship, detailed localities and vouchers, and source of cytogenetic and genomic data. Group: BL, broad-leaved Loliinae; FL, fine-leaved Loliinae; Sch, Schedonorus. Chromosome number (2n), Ploidy, Genome size (2C, pg), Monoploid genome size (1Cx, pg; 1Cx, Mbp) and Genbank accession codes for plastome and nuclear ribosomal 35S and 5S genes are given for each sample. Values in bold correspond to new data generated in this study. Outgroups used in the phylogenomic analyses: *Oryza sativa, Brachypodium distachyon*.

| **Taxon** | **Code** | **Group** | **Locality** | **2n** | **Ploidy** | **2C (pg)** | **1Cx (pg)** | **1Cx (Mbp)** | **Genbank accession No.** | | | **Data source** |
| --- | --- | --- | --- | --- | --- | --- | --- | --- | --- | --- | --- | --- |
|  |  |  |  |  |  |  |  |  | **Plastome** | **35S rDNA** | **5S rDNA** |  |
| *Festuca africana* (Hack.) Clayton | FF | Broad-Leaved | Uganda: Gahinga; Namaganda 190Vg; MHU1603 | 70 | 10x | --- | --- | --- | SAMN14647044 | MT145277 | **ON248974** | 2n: Namaganda et al., 2006; genome data: this study |
| *Festuca amplissima* Rupr. | GG | Broad-Leaved | Mexico: Chihuahua; Barranca del Cobre; PC. 17573 | 42 | 6x | --- | --- | --- | SAMN14647045 | MT145278 | **ON248975** | 2n: Bolkhouskikh et al. 1969; genome data: this study |
| *Festuca caldasii* (Kunth) Kunth | NN | Broad-Leaved | Ecuador: Catamayo; Chinchas-Tambara; HUTPL14055 | 28 | **4x** | **20.36** | 5.09 | 4978.02 | SAMN14647047 | MT145280 | **ON248977** | 2n, 2C, genome data: this study |
| *Festuca durandoi* Clauson | PP | Broad-Leaved | Portugal: Serra Arga; Alto do Espinheiro; | 14 | 2x | 14.66 (4x) | 3.66 | 3584.86 | SAMN14647050 | MT145283 | **ON248980** | 2n: Devesa & Martinez-Segarra 2020; 2C: Šmarda et al., 2008; genome data: this study |
| *Festuca lasto* Boiss. | HH | Broad-Leaved | Cadiz: Jerez de la Frontera; Los Alcornocales; UZ 29.08 | 14 | 2x | --- | --- | --- | SAMN14647058 | MT145291 | **ON248989** | 2n: Harper et al. 2004; genome data: this study |
| *Festuca mekiste* Clayton | AM | Broad-Leaved | Kenya: Mt. Elgon National Park, Kambi Mtamaiwa; Carvalho 4521 | --- | --- | --- | --- | --- | **SAMN27777779** | **ON243855** | **ON248992** | genome data: this study |
| *Festuca molokaiensis* Soreng, P.M. Peterson & Catalán | II | Broad-Leaved | USA: Hawai: Molokai, BISH 728771 | --- | --- | --- | --- | --- | SAMN14647061 | MT145294 | **ON248993** | genome data: this study |
| *Festuca paniculata* (L.) Schinz & Thell | QQ | Broad-Leaved | Spain: Caceres, Puerto de los Castaños, UZ 40.07 | 14 | 2x | 7.65 | 3.83 | 3740.85 | SAMN14647064 | MT145297 | **ON248996** | 2n, 2C: Šmarda et al., 2008; genome data: this study |
| *Festuca parvigluma* Steud. | JJ | Broad-Leaved | China: Baotianman; Henan; Neixiang Xian; MO 4922557 | 28 | 4x | --- | --- | --- | SAMN14647065 | MT145298 | **ON248997** | 2n: Tateoka 1954; genome data: this study |
| *Festuca scabra* Vahl | AP | Broad-Leaved | South Africa: KwaZulu Natal; Cathedral Peak; UZ-SA047 | 28 | 4x | --- | --- | --- | **SAMN27777781** | **ON243857** | **ON249003** | 2n: Spies and Plessis 1986; genome data: this study |
| *Festuca spectabilis* Jan | LL | Broad-Leaved | Bosnia-Hercegovina: Troglav; Sajkovacko zdrlo | 42 | 6x | --- | --- | --- | SAMN14647071 | MT145304 | **ON249004** | 2n: Markgraf-Dannenberg 1980; genome data: this study |
| *Festuca superba* Parodi ex Türpe | RR | Broad-Leaved | Argentina: Jujuy; Yala; Laguna Rodeo; PC 356.08 | 56 | 8x | --- | --- | --- | SAMN14647072 | MT145305 | **ON249005** | 2n: Dubcovsky and Martínez, 1992; genome data: this study |
| *Festuca triflora* J.F. Gmel. | MM | Broad-Leaved | Morocco: Rif Mountains, Bab Barret-Ketama; PC 39.17 | 14 | 2x | **7.84** | 3.92 | 3833.76 | SAMN14647073 | MT145306 | **ON249006** | 2n, 2C, genome data: this study |
| *Festuca abyssinica* Hochst. ex A. Rich. | AB | Fine-Leaved | Tanzania: Kilimanjaro; Afroalp O-DP-42737 | 28 | 4x | --- | --- | --- | SAMN14647043 | MT145276 | **ON248973** | 2n: Namaganda et al. 2006; genome data: this study |
| *Festuca asplundii* E.B. Alexeev | AC | Fine-Leaved | Ecuador: Loja; Saraguro; HUTPL14046 | --- | 6x | **21.23** | 3.54 | 3460.49 | SAMN14647046 | MT145279 | **ON248976** | 2n: Smarda & Stancik 2006; 2C, genome data: this study |
| *Festuca capillifolia* Dufour ex Roem. & Schult. | SS | Fine-Leaved | Morocco: Middle Atlas; Ifrane National Park; PC 77.17 | 14 | 2x | --- | --- | --- | SAMN14647048 | MT145281 | **ON248978** | 2n: Devesa & Martinez-Segarra 2020; genome data: this study |
| *Festuca chimborazensis* E.B. Alexeev subsp. *micacochensis* Stančík | AD | Fine-Leaved | Ecuador: Riobamba; Chimborazo; HUTPL14066 | 42 | 6x | **13.48** | 2.25 | 2197.24 | SAMN14647049 | MT145282 | **ON248979** | 2n, 2C, genome data: this study |
| *Festuca eskia* Ramond ex DC. | OO | Fine-Leaved | Spain: León, Picos de Europa, Colladines, FE321 | 14 | 2x | 5.7 | 2.85 | 2787.3 | SAMN14647051 | MT145284 | **ON248981** | 2n: Kerguelen 1975; 2C: Marques et al. 2016; genome data: this study |
| *Festuca fimbriata* Nees | AE | Fine-Leaved | Argentina: Misiones; Dpto. Apóstoles; UZ 498.08 | 42 | 6x | --- | --- | --- | SAMN14647053 | MT145286 | **ON248983** | 2n: Dubcovsky & Martinez 1992; genome data: this study |
| *Festuca francoi* Fern. Prieto, C. Aguiar, E. Días & M.I. Gut | TT | Fine-Leaved | Portugal: Açores; Terceira; MS4403 | 12 | 2x | --- | --- | --- | SAMN14647057 | MT145290 | **ON248984** | 2n: Sequeira et al. 2009; genome data: this study |
| *Festuca gracillima* Hook. F. | AF | Fine-Leaved | Argentina: Tierra de Fuego; Estancia San Pablo; UZ482.08 | 42 | 6x | --- | --- | --- | SAMN14647055 | MT145288 | **ON248986** | 2n: Dollenz 1978; genome data: this study |
| *Festuca holubii* Stančík | AG | Fine-Leaved | Ecuador: Saraguro; route to Cerro de Arcos; HUTPL14071 | --- | --- | --- | --- | --- | SAMN14647056 | MT145289 | **ON248988** | genome data: this study |
| *Festuca ovina* L. | UU | Fine-Leaved | Rusia: Leningradskaya Oblast’; Gatchinskii Raion; PC 54 | 14 | **2x** | 4.82 | 2.41 | 2356.98 | SAMN14647062 | MT145295 | **ON248994** | 2n, 2C: Smarda & al. 2008; genome data: this study |
| *Festuca pampeana* Speg. | VV | Fine-Leaved | Argentina: Buenos Aires; Sierra de la Ventana; PC 428.08 | 56 | 8x | --- | --- | --- | SAMN14647063 | MT145296 | **ON248995** | 2n: Dubcovsky & Martinez 1988; genome data: this study |
| *Festuca procera* Kunth | AH | Fine-Leaved | Ecuador:Chimborazo; Riobamba; HUTPL14079 | 28 | 4x | **14.88** | 3.72 | 3638.16 | SAMN14647067 | MT145299 | **ON248999** | 2n: Smarda & Stancik 2006; 2C, genome data: this study |
| *Festuca pyrenaica* Reut. | WW | Fine-Leaved | Spain: Huesca; Pyrenees, Tobacor | 28 | 4x | --- | --- | --- | SAMN14647068 | MT145300 | **ON249000** | 2n: Kerguelen 1975; genome data: this study |
| *Festuca pyrogea* Speg. | XX | Fine-Leaved | Argentina: Tierra de fuego, Cabo San Pablo; PC 494.08 | --- | --- | --- | --- | --- | SAMN14647069 | MT145302 | **ON249001** | genome data: this study |
| *Festuca rubra* L. | AN | Fine-Leaved | Argentina: Tierra de fuego; Cabo Annicolta; UZ 03.09 | 42 | **6x** | 13.68 | 2.28 | 2229.84 | **SAMN27777780** | **ON243856** | **ON249002** | 2n, 2C: Smarda et al. 2008; genome data: this study |
| *Megalachne masafuerana* (Skottsb. & Pilg. ex. Pilg.) Matthei | AJ | Fine-Leaved | Chile: Juan Fernandez islands; Masafuera. L. Gaete. 9150 (0S) | --- | --- | --- | --- | --- | SAMN14647075 | MT145308 | **ON249018** | genome data: this study |
| *Vulpia ciliata* Dumort. | YY | Fine-Leaved | Spain: Toledo; Mar de Ontígola; UZ 109.07 | 28 | 4x | 8.28 | 1.38 | 1349.64 | SAMN14647076 | MT145309 | **ON249009** | 2n: Stace & Cotton 1980; 2C: Smarda et al. 2008; genome data: this study |
| *Festuca arundinacea* Schreb. subsp. *arundinacea* | AX | Schedonorus | Spain: Coruña: Ferrol | 42 | 6x | 17.46 | 2.91 | 2845.98 | **SAMN27777774** | **ON243850** | **ON249007** | 2n, 2C: Kopecký et al., 2010; genome data: this study |
| *Festuca arundinacea* subsp*. atlantigena* (St.-Yves) Auquier | AY | Schedonorus | Morocco: Atlas mountains, ABY BN 807 | 56 | 8x | 16.22 | 2.03 | 1982.895 | **SAMN27777775** | **ON243851** | **ON248990** | 2n, 2C: Ezquerro-López et al., 2017; genome data: this study |
| *Festuca arundinacea* Schreb. subsp. *arundinacea* var. *letourneuxiana* (St.-Yves) Torrecilla & Catalán | CC | Schedonorus | Morocco: Atlas Mountains, ABY BN 400 | 70 | 10x | 19.7 | 1.97 | 1926.66 | SAMN14647059 | MT145292 | **ON249010** | 2n, 2C: Ezquerro-López et al., 2017; genome data: this study |
| *Festuca dracomontana* H.P. Linder | AQ | Schedonorus | South Africa: TVL; Haernertsburg; PRE 66429 | --- | --- | --- | --- | --- | **SAMN27777776** | **ON243852** | **ON249011** | genome data: this study |
| *Festuca fenas* Lag. | AA | Schedonorus | Spain:W Mediterranean; PI289654 | 28 | 4x | 10.48 | 2.62 | 2562.36 | SAMN14647052 | MT145285 | **ON248982** | 2n, 2C: Ezquerro-López et al. 2017; genome data: this study |
| *Festuca fontqueri* St.-Yves | BB | Schedonorus | Morocco: Rif Mountains; Talassemtane National Park, PC 59.17 | 14 | 2x | **5.54** | 2.77 | 2709.06 | SAMN14647054 | MT145287 | **ON249008** | 2n: Favarger et al. 1980; 2C, genome data: this study |
| *Festuca gigantea* (L.) Vill. | AO | Schedonorus | Norway: cultivated; 12/P2007 | 42 | 6x | 20.75 | 3.46 | 3382.25 | **SAMN27777777** | **ON243853** | **ON248985** | 2n, 2C: Smarda et al. 2008; genome data: this study |
| *Festuca gudoschnikovii* Stepanov | AR | Schedonorus | Russia:Krasnoyarskii Krai; Yermakovskii Raion; PC 87 | 28 | 4x | --- | --- | --- | **SAMN27777778** | **ON243854** | **ON248987** | 2n: Probatova et al. 2017; genome data: this study |
| *Festuca mairei* St.-Yves | DD | Schedonorus | Morocco: Atlas Mountains; PI-610941 | 28 | 4x | 10.04 | 2.51 | 2454.78 | SAMN14647060 | MT145293 | **ON248991** | 2n, 2C: Ezquerro-López et al. 2017; genome data: this study |
| *Festuca pratensis* Huds. | EE | Schedonorus | UK: England, USDA PI 283306 | 14 | 2x | 6.5 | 3.25 | 3178.5 | SAMN14647066 | MT145301 | **ON248998** | 2n, 2C: Kopecky et al. 2010; genome data: this study |
| *Festuca simensis* Hochst. ex A. Rich. | AS | Schedonorus | Kenya: Mt. Kenya, Meteorological station; Namaganda 1750 | 28 | 4x | --- | --- | --- | **SAMN27777782** | **ON243858** | **ON249012** | 2n: Namaganda et al. 2006; genome data: this study |
| *Lolium canariense* Steud | AT | Schedonorus | Spain: Canary Islands; USDA PI 320544 | 14 | 2x | 4.3 | 2.15 | 2102.7 | **SAMN27777783** | **ON243859** | **ON249013** | 2n: Inda & Wolny 2013; 2C: Huchinson et al. 1979; genome data: this study |
| *Lolium perenne* L | AL | Schedonorus | UK: Wales; USDA PI 619001 | 14 | 2x | 5.51 | 2.76 | 2694.39 | **SAMN27777784** | **ON243860** | **ON249014** | 2n: Inda & Wolny 2013; 2C: Smarda et al. 2008; genome data: this study |
| *Lolium persicum* Boiss. & Hohen | AK | Schedonorus | Georgia; USDA PI 314446 | 14 | 2x | 6.4 | 3.2 | 3129.6 | **SAMN27777785** | **ON243861** | **ON249015** | 2n: Inda & Wolny 2013; 2C: Huchinson et al. 1979; genome data: this study |
| *Lolium rigidum* Gaudin | AV | Schedonorus | Turkey; USDA PI 545604 | 14 | 2x | 5.49 | 2.75 | 2684.61 | **SAMN27777786** | **ON243862** | **ON249017** | 2n: Inda and Wolny, 2013; 2C: Smarda et al. 2008; genome data: this study |
| *Lolium saxatile* H. Scholz & S. Scholz | AW | Schedonorus | Spain: Canary islands; AS | --- | --- | --- | --- | --- | **SAMN27777787** | **ON243863** | **ON249016** | genome data: this study |
| *Micropyropsis tuberosa* Romero-Zarco & Cabezudo | AU | Schedonorus | Spain: Huelva: Almonte; UZ89.07 | 14 | 2x | --- | --- | --- | **SAMN27777788** | **ON243864** | **ON249019** | 2n: Romero-Zarco 1988; genome data: this study |
| *Brachypodium distachyon* (L.) P.Beauv. | --- | --- | Spain: Caceres; UZ 28.07 | 10 | 2x | --- | --- | --- | NC_011032.1 | Phytozome Bd21 v.3.1 | ---- | ---- |
| *Oryza sativa* L. | --- | --- | China: National Hybrid Rice Research and Development Center (NHRRDC); China |  | 2x | --- | --- | --- | AY522331.1 | AP008215 | ---- | ---- |
| *Oryza eichingeri*  Peter | --- | --- | Philippines: International Rice Reserch Institute (IRRI) | --- | 2x | --- | --- | --- | ---- | ---- | EF197127.1 | ---- |

References for Supplementary Table S1:

Bolkhovskikh Z., Grif, V. Matvejeva, T. Zakhareyeva, O. (1969). *Chromosome numbers of flowering plants*. Leningrad. V.L. Komarov Botanical Institute

Devesa JA and Martínez-Segarra G. (2020). “*Festuca* subgen. *Festuca*”. in *Flora Ibérica* Vol. XIX (I). ed. J. A. Devesa. Real Jardin Botánico. Madrid.

Dollenz, O. (1978). Números cromosómicos de espermatófitas de los géneros Berberis, Ribes y Festuca. *Anales Inst. Patagonia, Ser. Ci. Nat*. 9, 141–144.

Dubcovsky J. and Martínez A. (1988). Cariotipos y comportamiento meiótico de las especies de *Festuca* (Poaceae) endémicas de Sierra de la Ventana. *Bol. Soc. Argent. Bot*. 25, 415–423.

Dubcovsky, J., and Martínez, A. (1992). Distribución geográfica de los niveles de ploidía en *Festuca*. *Parodiana* 7, 91–99.

Ezquerro-López, D., Kopecký, D., and Inda, L. A. (2017). Cytogenetic relationships within the Maghrebian clade of Festuca subgen. Schedonorus (Poaceae), using flow cytometry and FISH. *An. del Jard. Bot. Madrid* 74, 1–9.

Favarger C, Galland N., Küpfer, Ph. (1980). Recherches cytotaxonomiques sur la flore orophile du Maroc. *Naturalia Monspe. Sér. Bot*. 29, 1–64.

Harper JA, ThomasID, JA Lovatt JA, Thomas, H M. (2004). Physical mapping of rDNA sites in possible diploid progenitors of polyploid *Festuca* species. *Pl. Syst. Evol*. 245, 163–168.

Hutchinson J, Rees H, Seal AG. (1979). An assay of the activity of supplementary DNA in Lolium. *Heredity* 43, 411–421.

Inda, L. A., and Wolny, E. (2013). Fluorescent in situ hybridization of the ribosomal RNA genes (5S and 35S) in the genus Lolium: Lolium canariense, the missing link with Festuca? *An. del Jard. Bot. Madrid* 70, 97–102.

Kerguelen M. (1975). Les Gramineae (Poaceae) de la flore Francaise essai de mise au point taxonomique et nomenclaturale. *Lejeunia* 75, 1–343.

Kopecký, D., Havránková, M., Loureiro, J., Castro, S., Lukaszewski, A. J., Bartoš, J., et al. (2010). Physical distribution of homoeologous recombination in individual chromosomes of Festuca pratensis in Lolium multiflorum. *Cytogenet. Genome Res.* 129, 162–172.

Markgraf-Dannenberg I. (1980). "*Festuca*". in: Flora Europea Vol. 5. eds. Tutin et al. Cambridge University Press.

Marques I, Draper D, López-Herranz ML, Garnatje T, Segarra-Moragues JG, Catalán P. (2016). Past climate changes facilitated homoploid speciation in three mountain spiny fescues (Festuca, Poaceae). *Sci. Reports* 6, 36283.

Namaganda, M., Lye, K. A., Friebe, B., and Heun, M. (2006). AFLP-based differentiation of tropical African Festuca species compared to the European Festuca complex. *Theor. Appl. Genet.* 113, 1529–1538.

Probatova NS, Barkalov Nikolai VY, Stepanov NV. (2017). Chromosome numbers in some vascular plant species from Siberia and the Russian Far East. *Botanica Pacifica* 6, 51–55.

Romero-Zarco C. (1988). Números cromosomáticos de plantas occidentales. *An. del Jard. Bot. Madrid* 45, 472–486.

Sequeira M, Díaz Pérez A, Santos Guerra A, Viruel J, Catalán Rodríguez P. (2009). Karyological analysis of the five native Macaronesian Festuca (Gramineae) grasses supports a distinct diploid origin of two schizoendemic groups. *An. del Jard. Bot. Madrid* 66, 55–63.

Šmarda P. and Stancik D. (2006). Ploidy level variability in South American fescues (Festuca L., Poaceae): use of flow cytometry in up to 5 1/2-year-old caryopses and herbarium specimens. *Plant Biology* 8, 73–80.

Šmarda, P., Bureš, P., Horová, L., Foggi, B., and Rossi, G. (2008). Genome size and GC content evolution of Festuca: Ancestral expansion and subsequent reduction. *Ann. Bot.* 101, 421–433.

Spies JJ and Plessis H. (1986). Chromosome studies on African plants. 2. *Brothalia* 16, 269–270.

Stace CA and Cotton R. (1980). "*Vulpia*". in *Flora Europea Vol. 5*. T eds. Tutin et al. Cambridge University Press.

Tateoka T. (1954). Karyotaxonomy in Poaceae II. Somatic chromosomes of some species. *Cytologia* 19, 317–328.
