# Supplementary material for: Evolutionary Dynamics of the Repeatome Explains Contrasting Differences in Genome Sizes and Hybrid and Polyploid Origins of Grass Loliinae Lineages
Source: Front Plant Sci. 2022 Jul 1;13:901733. doi: 10.3389/fpls.2022.901733 (PMC9284676; doi:10.3389/fpls.2022.901733)
Supplement: Supplementary Table 2 — Loliinae samples used in the repetitive DNA analysis. Genome skimming paired-end (PE) reads per sample and PE reads selected by Repeat Explorer 2 per sample in each of the comparative analyses of the four Loliinae groups: Loliinae, BL (broad-leaved Loliinae), FL (fine-leaved Loliinae), Schedonorus. [file Table_2.DOCX]

**Table S2.**  Loliinae samples used in the repetitive DNA analysis. Genome skimming paired-end (PE) reads per sample and PE reads selected by Repeat Explorer2 per sample in each of the comparative analyses of the four Loliinae groups: Loliinae, BL (broad-leaved Loliinae), FL (fine-leaved Loliinae), Schedonorus.

| **Taxon** | **Code** | **Group** | ***Genome skimming* reads** | **Insert size** | **Loliinae** | | **Schenodorus** | | **Broad_leaved** | | **Fine_leaved** | |
| --- | --- | --- | --- | --- | --- | --- | --- | --- | --- | --- | --- | --- |
|  |  |  |  |  | **Repeat Explorer reads** | **Repeat Explorer**  **Genome coverage** | **Repeat Explorer**  **reads** | **Repeat Explorer**  **Genome coverage** | **Repeat Explorer reads** | **Repeat Explorer**  **Genome coverage** | **Repeat**  **Explorer reads** | **Repeat**  **Explorer Genome coverage** |
| *Festuca africana* (Hack.) Clayton | FF | Broad-Leaved | 13,549,000 | 195 | 93,068 | --- | --- | --- | 231,344 | --- | --- | --- |
| *Festuca amplissima* Rupr. | GG | Broad-Leaved | 12,058,000 | 220 | 83,358 | --- | --- | --- | 204,494 | --- | --- | --- |
| *Festuca caldasii* (Kunth) Kunth | NN | Broad-Leaved | 9,863,000 | 248 | 68,088 | 0.094452353 | --- | --- | 168,162 | 0.08957966 |  | --- |
| *Festuca durandoi* Clauson | PP | Broad-Leaved | 12,688,000 | 217 | 87,702 | 0.131176665 | --- | --- | 215,648 | --- | --- | --- |
| *Festuca lasto* Boiss. | HH | Broad-Leaved | 21,581,000 | 300 | 148,516 | --- | --- | --- | 367,034 | --- | --- | --- |
| *Festuca mekiste* Clayton | AM | Broad-Leaved | 16,245,000 | 201 | 111,228 | --- | --- | --- | 276,810 | --- | --- | --- |
| *Festuca molokaiensis* Soreng, P.M. Peterson & Catalán | II | Broad-Leaved | 12,188,000 | 219 | 83,800 | --- | --- | --- | 206,804 | --- | --- | --- |
| *Festuca paniculata* (L.) Schinz & Thell | QQ | Broad-Leaved | 35,808,000 | 300 | 246,446 | 0.125689537 | --- | --- | 609,220 | 0.11920535 | --- | --- |
| *Festuca parvigluma* Steud. | JJ | Broad-Leaved | 15,872,000 | 190 | 108,708 | --- | --- | --- | 270,022 | --- | --- | --- |
| *Festuca scabra* Vahl | AP | Broad-Leaved | 21,174,000 | 211 | 145,346 | --- | --- | --- | 359,062 | --- | --- | --- |
| *Festuca spectabilis* Jan | LL | Broad-Leaved | 12,960,000 | 221 | 89,008 | --- | --- | --- | 220,088 | --- | --- | --- |
| *Festuca superba* Parodi ex Türpe | RR | Broad-Leaved | 12,193,000 | 221 | 83,988 | --- | --- | --- | 207,694 | --- | --- | --- |
| *Festuca triflora* J.F. Gmel. | MM | Broad-Leaved | 24,472,000 | 300 | 168,142 | 0.122643489 | --- | --- | 416,196 | 0.11631645 | --- | --- |
| *Festuca abyssinica* Hochst. ex A. Rich. | AB | Fine-Leaved | 12,041,000 | 166 | 82,794 | --- | --- | --- | --- | --- | 158,960 | --- |
| *Festuca asplundii* E.B. Alexeev | AC | Fine-Leaved | 25,088,000 | 300 | 172,022 | 0.13587258 | --- | --- | --- | --- | 331,124 | 0.114396833 |
| *Festuca capillifolia* Dufour ex Roem. & Schult. | SS | Fine-Leaved | 13,430,000 | 228 | 92,674 | --- | --- | --- | --- | --- | 177,206 | --- |
| *Festuca chimborazensis* E.B. Alexeev subsp. *micacochensis* Stančík | AD | Fine-Leaved | 10,913,000 | 254 | 75,418 | 0.213989234 | --- | --- | --- | --- | 143,276 | 0.180166525 |
| *Festuca eskia* Ramond ex DC. | OO | Fine-Leaved | 24,041,000 | 300 | 164,798 | 0.168688589 | --- | --- | --- | --- | 317,762 | 0.142026009 |
| *Festuca fimbriata* Nees | AE | Fine-Leaved | 15,741,000 | 173 | 107,956 | --- | --- | --- | --- | --- | 207,162 | --- |
| *Festuca francoi* Fern. Prieto, C. Aguiar, E. Días & M.I. Gut | TT | Fine-Leaved | 17,592,000 | 186 | 120,860 | --- | --- | --- | --- | --- | 231,806 | --- |
| *Festuca gracillima* Hook. F. | AF | Fine-Leaved | 13,888,000 | 224 | 95,164 | --- | --- | --- | --- | --- | 183,168 | --- |
| *Festuca holubii* Stančík | AG | Fine-Leaved | 10,264,000 | 249 | 70,992 | --- | --- | --- | --- | --- | 135,198 | --- |
| *Festuca ovina* L. | UU | Fine-Leaved | 11,364,000 | 215 | 78,084 | 0.199486506 | --- | --- | --- | --- | 149,724 | 0.167956069 |
| *Festuca pampeana* Speg. | VV | Fine-Leaved | 14,862,000 | 223 | 102,358 | --- | --- | --- | --- | --- | 196,018 | --- |
| *Festuca procera* Kunth | AH | Fine-Leaved | 40,669,000 | 263 | 280,040 | 0.129237225 | --- | --- | --- | --- | 536,736 | 0.108810249 |
| *Festuca pyrenaica* Reut. | WW | Fine-Leaved | 30,021,000 | 300 | 205,996 | --- | --- | --- | --- | --- | 395,608 | --- |
| *Festuca pyrogea* Speg. | XX | Fine-Leaved | 16,835,000 | 193 | 115,054 | --- | --- | --- | --- | --- | 221,512 | --- |
| *Festuca rubra* L. | AN | Fine-Leaved | 25,260,000 | 220 | 174,248 | 0.210860736 | --- | --- | --- | --- | 333,992 | 0.177532512 |
| *Megalachne masafuerana* (Skottsb. & Pilg. ex. Pilg.) Matthei | AJ | Fine-Leaved | 6,134,000 | 184 | 43,824 | --- | --- | --- | --- | --- | 82,838 | --- |
| *Vulpia ciliata* Dumort. | YY | Fine-Leaved | 11,801,000 | 220 | 81,058 | 0.348378608 | --- | --- | --- | --- | 155,632 | 0.293314585 |
| *Festuca arundinacea* Schreb. subsp. *arundinacea* | AX | Schedonorus | 15,556,000 | 206 | --- | --- | 136,784 | 0.09663341 | --- | --- | --- | --- |
| *Festuca arundinacea* Schreb. subsp. *arundinacea* var. *letourneuxiana* (St.-Yves) Torrecilla & Catalán | CC | Schedonorus | 16,839,000 | 250 | 116,320 | 0.244041867 | 151,754 | 0.14274275 | --- | --- | --- | --- |
| *Festuca arundinacea* subsp. *atlantigena* (St.-Yves) Auquier | AY | Schedonorus | 15,091,000 | 224 | --- | --- | 140,518 | 0.13869455 | --- | --- | --- | --- |
| *Festuca dracomontana* H.P. Linder | AQ | Schedonorus | 15,835,000 | 139 | --- | --- | 142,276 | --- | --- | --- | --- | --- |
| *Festuca fenas* Lag. | AA | Schedonorus | 16,112,000 | 271 | 109,944 | 0.183497129 | 145,146 | 0.10732947 | --- | --- | --- | --- |
| *Festuca fontqueri* St.-Yves | BB | Schedonorus | 22,187,000 | 300 | 152,486 | 0.173560462 | 200,066 | 0.1015174 | --- | --- | --- | --- |
| *Festuca gigantea* (L.) Vill. | AO | Schedonorus | 20,914,000 | 223 | 143,564 | 0.139015656 | 188,600 | 0.08131177 | --- | --- | --- | --- |
| *Festuca gudoschnikovii* Stepanov | AR | Schedonorus | 13,994,000 | 208.75 | --- | --- | 126,240 | --- | --- | --- | --- | --- |
| *Festuca mairei* St.-Yves | DD | Schedonorus | 19,134,000 | 254 | 131,436 | 0.191538836 | 172,776 | 0.11203315 | --- | --- | --- | --- |
| *Festuca pratensis* Huds. | EE | Schedonorus | 12,189,000 | 271 | 83,958 | 0.147926916 | 109,432 | 0.08652406 | 207.212 | 0.14029553 | --- | --- |
| *Festuca simensis* Hochst. ex A. Rich. | AS | Schedonorus | 14,159,000 | 192.5 | --- | --- | 127,318 | --- | --- | --- | --- | --- |
| *Lolium canariense* Steud | AT | Schedonorus | 16,359,000 | 231.5 | --- | --- | 147,858 | 0.13079219 | --- | --- | --- | --- |
| *Lolium perenne* L | AL | Schedonorus | 28,103,000 | 244 | 192,992 | 0.174505437 | 253,800 | 0.10207013 | 478.612 | 0.16550289 | --- | --- |
| *Lolium persicum* Boiss. & Hohen | AK | Schedonorus | 25,523,000 | 241 | 175,334 | 0.150238275 | 229,658 | 0.087876 | --- | --- | --- | --- |
| *Lolium rigidum* Gaudin | AV | Schedonorus | 16,730,000 | 201.5 | --- | --- | 150,880 | 0.10244197 | --- | --- | --- | --- |
| *Lolium saxatile* H. Scholz & S. Scholz | AW | Schedonorus | 16,001,000 | 236 | --- | --- | 144,232 | --- | --- | --- | --- | --- |
| *Micropyropsis tuberosa* Romero-Zarco & Cabezudo | AU | Schedonorus | 19,803,000 | NA | --- | --- | 178,422 | --- | --- | --- | --- | --- |
