# Supplementary material for: Evolutionary Dynamics of the Repeatome Explains Contrasting Differences in Genome Sizes and Hybrid and Polyploid Origins of Grass Loliinae Lineages
Source: Front Plant Sci. 2022 Jul 1;13:901733. doi: 10.3389/fpls.2022.901733 (PMC9284676; doi:10.3389/fpls.2022.901733)
Supplement: Supplementary Table 5 — Phylogenetic signal based on Blomberg’s K values of repeat cluster contents obtained from the comparative RE2 analysis of Loliinae samples assessed in each of the four Loliinae groups: (A) Loliinae (38 samples, 38 clusters), (B) Broad-leaved (BL) Loliinae (15 samples, 96 clusters), (C) fine-leaved (FL) Loliinae (17 samples, 122 clusters), (D) Schedonorus (16 samples, 167 clusters), using the phylosig option of the phytools R package. Cluster abundance values (number of PE reads) are indicated in Supplementary Table 4. K values close to one indicate phylogenetic signal, values close to zero phylogenetic independence, and values >1 more phylogenetic signal than expected. p-Values based on 1000 randomizations. Significant values are highlighted in bold. [file Table_5.DOCX]

**Supplementary** **Table S5**. Phylogenetic signal based on Blomberg’s K values of repeat cluster contents obtained from the comparative RE2 analysis of Loliinae samples assessed in each of the four Loliinae groups, (**A**) Loliinae (38 samples, 39 clusters), (**B**) Broad-leaved (BL) Loliinae (13 samples, 96 clusters), (**C**) Fine-leaved (FL) Loliinae (17 samples, 122 clusters), (**D**) Schedonorus (16 samples, 167 clusters), using the *phylosig* option of the *phytools* R package. Cluster abundance values (number of PE reads) are indicated in Supplementary Table S3B. K values close to one indicate phylogenetic signal, values close to zero phylogenetic independence, and values >1 more phylogenetic signal than expected. p-values based on 1000 randomizations. Significant values are highlighted in bold.

1. Loliinae

| **N. Cluster** | **Cluster_Name** | **Phylogenetic signal on combined tree** | |
| --- | --- | --- | --- |
|  |  | **K** | **p-value** |
| 35 | LTR_1 | 0.373703 | 0.2050 |
| 39 | **LTR_2** | **0.583327** | **0.0060** |
| 86 | LTR_3 | 0.267521 | 0.6150 |
| 6 | Angela_1 | 0.441390 | 0.0960 |
| 7 | **Angela_2** | **0.847919** | **0.0020** |
| 12 | Angela_3 | 0.465467 | 0.0310 |
| 13 | Angela_4 | 0.520696 | 0.0210 |
| 14 | Angela_5 | 0.513860 | 0.0200 |
| 17 | Angela_6 | 0.415452 | 0.1400 |
| 30 | Angela_7 | 0.415190 | 0.1060 |
| 34 | Angela_8 | 0.399333 | 0.1240 |
| 53 | **Angela_9** | **0.649828** | **0.0030** |
| 71 | Angela_10 | 0.448955 | 0.0910 |
| 94 | **Angela_11** | **0.636886** | **0.0040** |
| 107 | **Angela_12** | **0.743470** | **0.0010** |
| 3 | SIRE_1 | 0.411074 | 0.1290 |
| 9 | SIRE_2 | 0.313160 | 0.4690 |
| 22 | SIRE_3 | 0.361960 | 0.2340 |
| 29 | SIRE_4 | 0.380547 | 0.1730 |
| 58 | SIRE_5 | 0.321802 | 0.4230 |
| 67 | SIRE_6 | 0.329360 | 0.4050 |
| 84 | SIRE_7 | 0.330350 | 0.3800 |
| 85 | SIRE_8 | 0.355221 | 0.3070 |
| 103 | SIRE_9 | 0.333838 | 0.4040 |
| 126 | **SIRE_10** | **0.463612** | **0.0490** |
| 137 | SIRE_11 | 0.339207 | 0.3850 |
| 50 | TAR_1 | 0.358543 | 0.2190 |
| 139 | TAR_2 | 0.279301 | 0.5300 |
| 28 | Athila_1 | 0.386600 | 0.0910 |
| 41 | EnSpm_CACTA_1 | 0.375853 | 0.1800 |
| 59 | **EnSpm_CACTA_2** | **0.475749** | **0.0530** |
| 75 | EnSpm_CACTA_3 | 0.456950 | 0.1710 |
| 87 | **EnSpm_CACTA_4** | **0.555016** | **0.0520** |
| 108 | **EnSpm_CACTA_5** | **0.503111** | **0.0510** |
| 180 | EnSpm_CACTA_6 | 0.374766 | 0.1810 |
| 106 | .45S_rDNA_1 | 0.209001 | 0.8380 |
| 115 | 45S_rDNA_2 | 0.190001 | 0.8970 |
| 203 | 5S_rDNA_1 | 0.177649 | 0.9110 |

(B) Broad-leaved

| **N. Cluster** | **Cluster Name** | **Phylogenetic signal on combined tree** | | | **N. Cluster** | **Cluster**  **Name** | **Phylogenetic signal on combined tree** | |
| --- | --- | --- | --- | --- | --- | --- | --- | --- |
|  |  | **K** | **p-value** |  | |  | **K** | **p-value** |
| 40 | Repeat_1 | 0.3435 | 0.5850 | 1 | | Tekay_1 | 0.5706 | 0.1220 |
| 57 | Repeat_2 | 0.4259 | 0.3090 | 4 | | **Tekay_2** | **1.0530** | **0.0080** |
| 58 | Repeat_3 | 0.3954 | 0.4410 | 7 | | **Tekay_3** | **0.6307** | **0.0730** |
| 70 | Repeat_4 | 0.4548 | 0.2880 | 8 | | Tekay_4 | 0.3789 | 0.4640 |
| 84 | Repeat_5 | 0.3713 | 0.4890 | 10 | | **Tekay_5** | **0.7060** | **0.0780** |
| 108 | Repeat_6 | 0.2951 | 0.7690 | 19 | | **Tekay_6** | **0.9883** | **0.0230** |
| 110 | Repeat_7 | 0.3704 | 0.5180 | 27 | | Tekay_7 | 0.6976 | 0.0370 |
| 179 | Repeat_8 | 0.4154 | 0.3740 | 43 | | Tekay_8 | 1.1727 | 0.0040 |
| 189 | Repeat_9 | 0.3301 | 0.6560 | 46 | | Tekay_9 | 0.3280 | 0.6190 |
| 218 | Repeat_10 | 0.2661 | 0.8800 | 49 | | **Tekay_10** | **0.9624** | **0.0080** |
| 100 | LINE_1 | 0.3406 | 0.6240 | 62 | | **Tekay_11** | **1.0418** | **0.0080** |
| 12 | **LTR_1** | **1.0796** | **0.0050** | 65 | | **Tekay_12** | **1.0957** | **0.0020** |
| 256 | Ale_1 | 0.2603 | 0.7670 | 114 | | **Tekay_13** | **0.8371** | **0.0230** |
| 2 | **Angela_1** | **0.6062** | **0.0830** | 118 | | Tekay_14 | 0.5686 | 0.1650 |
| 3 | Angela_2 | 0.3894 | 0.4440 | 129 | | Tekay_15 | 0.6237 | 0.1210 |
| 5 | Angela_3 | 0.3994 | 0.3840 | 132 | | Tekay_16 | 0.3177 | 0.6310 |
| 13 | Angela_4 | 0.5182 | 0.1780 | 24 | | Athila_1 | 0.2142 | 0.9100 |
| 21 | **Angela_5** | **0.6538** | **0.0540** | 42 | | **Athila_2** | **0.6794** | **0.0550** |
| 22 | Angela_6 | 1.2911 | 0.0020 | 48 | | Athila_3 | 0.5150 | 0.1750 |
| 31 | Angela_7 | 0.5303 | 0.1320 | 55 | | Athila_4 | 0.4772 | 0.2190 |
| 32 | Angela_8 | 0.5690 | 0.0950 | 60 | | **Athila_5** | **0.6296** | **0.0800** |
| 34 | Angela_9 | 0.5083 | 0.1890 | 80 | | Athila_6 | 0.2239 | 0.8610 |
| 39 | Angela_10 | 0.5176 | 0.1500 | 97 | | **Athila_7** | **0.7035** | **0.0540** |
| 53 | Angela_11 | 0.3974 | 0.3990 | 104 | | **Athila_8** | **0.6905** | **0.0380** |
| 64 | Angela_12 | 0.4498 | 0.2500 | 185 | | Athila_9 | 0.5642 | 0.1200 |
| 72 | Angela_13 | 0.4298 | 0.3210 | 11 | | Retand_1 | 0.5333 | 0.1620 |
| 79 | Angela_14 | 0.4175 | 0.3480 | 45 | | EnSpm_CACTA_1 | 0.4333 | 0.4040 |
| 81 | **Angela_15** | **0.7050** | **0.0450** | 47 | | EnSpm_CACTA_2 | 0.5681 | 0.1320 |
| 95 | Angela_16 | 0.3509 | 0.5720 | 59 | | EnSpm_CACTA_3 | 0.4286 | 0.3510 |
| 162 | Angela_17 | 0.4655 | 0.2330 | 127 | | EnSpm_CACTA_4 | 0.3997 | 0.4200 |
| 198 | Ikeros_1 | 0.4370 | 0.2990 | 140 | | EnSpm_CACTA_5 | 0.5053 | 0.2650 |
| 18 | SIRE_1 | 0.4566 | 0.2890 | 144 | | EnSpm_CACTA_6 | 0.4883 | 0.2060 |
| 26 | SIRE_2 | 0.4068 | 0.3970 | 150 | | EnSpm_CACTA_7 | 0.4796 | 0.2840 |
| 35 | SIRE_2.1 | 0.5107 | 0.1750 | 176 | | EnSpm_CACTA_8 | 0.2484 | 0.8980 |
| 68 | SIRE_4 | 0.3873 | 0.4810 | 199 | | EnSpm_CACTA_9 | 0.5272 | 0.1890 |
| 99 | SIRE_5 | 0.4136 | 0.4120 | 86 | | **MuDR_Mutator_1** | **0.9483** | **0.0020** |
| 192 | SIRE_6 | 0.3916 | 0.4380 | 219 | | MuDR_Mutator_2 | 0.5809 | 0.1410 |
| 28 | TAR_1 | 0.3529 | 0.5740 | 190 | | PIF_Harbinger_1 | 0.2860 | 0.7130 |
| 194 | Tork_1 | 0.3740 | 0.4450 | 98 | | 45S_rDNA_1 | 0.2531 | 0.8490 |
| 16 | CRM_1 | 0.3987 | 0.4300 | 133 | | 45S_rDNA_2 | 0.2497 | 0.8550 |
| 82 | CRM_2 | 0.6587 | 0.0990 | 236 | | 5S_rDNA_1 | 0.3941 | 0.4240 |
| 116 | CRM_3 | 0.5217 | 0.1830 | 107 | | Satellite_1 | 0.5113 | 0.2440 |
| 223 | CRM_4 | 0.3788 | 0.4460 |  | |  |  |  |

(C) Fine-leaved

| **N. Cluster** | **Cluster Name** | **Phylogenetic signal on combined tree** | | **N. Cluster** | | **Cluster Name** | | **Phylogenetic signal on combined tree** | | |
| --- | --- | --- | --- | --- | --- | --- | --- | --- | --- | --- |
|  |  | **K** | **p-value** | |  | |  | | **K** | **p-value** |
| 18 | LTR_1 | 0.5850 | 0.3040 | | 178 | | Ivana | | 0.6122 | 0.2780 |
| 124 | LTR_10 | 0.4946 | 0.5210 | | 1 | | SIRE_1 | | 0.4255 | 0.6880 |
| 36 | LTR_2 | 0.3961 | 0.7580 | | 10 | | SIRE_2 | | 0.4795 | 0.5640 |
| 52 | LTR_3 | 0.4874 | 0.4790 | | 17 | | SIRE_3 | | 0.4726 | 0.5560 |
| 61 | LTR_4 | 0.3825 | 0.7960 | | 20 | | SIRE_4 | | 0.5103 | 0.4530 |
| 79 | LTR_5 | 0.6444 | 0.1800 | | 22 | | SIRE_5 | | 0.4442 | 0.6720 |
| 82 | LTR_6 | 0.5286 | 0.4160 | | 33 | | SIRE_6 | | 0.6636 | 0.1920 |
| 92 | LTR_7 | 0.5928 | 0.2790 | | 43 | | SIRE_7 | | 0.4188 | 0.6890 |
| 107 | LTR_8 | 0.6859 | 0.1720 | | 58 | | SIRE_8 | | 0.4299 | 0.6800 |
| 121 | LTR_9 | 0.4593 | 0.6240 | | 66 | | SIRE_9 | | 0.4203 | 0.7200 |
| 51 | 45S_rDNA_1 | 0.5162 | 0.4370 | | 68 | | SIRE_10 | | 0.6319 | 0.2110 |
| 112 | 45S_rDNA_2 | 0.6238 | 0.2660 | | 71 | | SIRE_11 | | 0.5350 | 0.3940 |
| 183 | 5S_rDNA | 0.5924 | 0.2420 | | 86 | | SIRE_12 | | 0.5048 | 0.4990 |
| 16 | **Repeat_1** | **1.0080** | **0.0250** | | 114 | | SIRE_13 | | 0.4121 | 0.7350 |
| 24 | Repeat_2 | 0.4221 | 0.7190 | | 15 | | TAR | | 0.5817 | 0.3120 |
| 67 | Repeat_3 | 0.3501 | 0.8790 | | 145 | | Tork | | 0.6339 | 0.1990 |
| 140 | Repeat_4 | 0.4277 | 0.7000 | | 25 | | Athila_1 | | 0.3757 | 0.8400 |
| 34 | Satellite_1 | 0.5174 | 0.4740 | | 27 | | Athila_2 | | 0.4745 | 0.5670 |
| 96 | Satellite_2 | 0.3895 | 0.7970 | | 32 | | Athila_3 | | 0.4377 | 0.6350 |
| 6 | EnSpm_CACTA_1 | 0.4334 | 0.6900 | | 39 | | Athila_4 | | 0.3759 | 0.8270 |
| 9 | EnSpm_CACTA_2 | 0.4903 | 0.4900 | | 40 | | Athila_5 | | 0.5311 | 0.3540 |
| 46 | EnSpm_CACTA_3 | 0.4561 | 0.6060 | | 47 | | Athila_6 | | 0.4955 | 0.4820 |
| 49 | EnSpm_CACTA_4 | 0.4501 | 0.5980 | | 54 | | Athila_7 | | 0.4510 | 0.5860 |
| 55 | EnSpm_CACTA_5 | 0.5194 | 0.4530 | | 81 | | Athila_8 | | 0.4363 | 0.6530 |
| 60 | EnSpm_CACTA_6 | 0.4620 | 0.6040 | | 131 | | Athila_9 | | 0.4843 | 0.4760 |
| 64 | EnSpm_CACTA_7 | 0.6836 | 0.2210 | | 135 | | Athila_10 | | 0.4966 | 0.4560 |
| 73 | EnSpm_CACTA_8 | 0.4144 | 0.7190 | | 138 | | Athila_11 | | 0.6256 | 0.1840 |
| 80 | EnSpm_CACTA_9 | 0.4872 | 0.5390 | | 149 | | Athila_12 | | 0.3980 | 0.7520 |
| 105 | EnSpm_CACTA_10 | 0.5294 | 0.4210 | | 187 | | Athila_13 | | 0.4041 | 0.7130 |
| 125 | EnSpm_CACTA_11 | 0.4066 | 0.7510 | | 42 | | CRM_1 | | 0.6362 | 0.2460 |
| 144 | EnSpm_CACTA_12 | 0.5082 | 0.4910 | | 59 | | CRM_2 | | 0.3588 | 0.8750 |
| 157 | EnSpm_CACTA_13 | 0.5125 | 0.4800 | | 100 | | CRM_3 | | 0.3510 | 0.8600 |
| 45 | MuDR_Mutator_1 | 0.5426 | 0.3880 | | 103 | | CRM_4 | | 0.3466 | 0.8890 |
| 101 | MuDR_Mutator_2 | 0.5144 | 0.4370 | | 116 | | CRM_5 | | 0.6577 | 0.2860 |
| 219 | MuDR_Mutator_3 | 0.7445 | 0.2030 | | 126 | | CRM_6 | | 0.3726 | 0.8240 |
| 229 | MuDR_Mutator_4 | 0.6158 | 0.3420 | | 136 | | CRM_7 | | 0.6476 | 0.2630 |
| 197 | PIF_Harbinger | 0.5061 | 0.4370 | | 170 | | CRM_8 | | 0.4988 | 0.4740 |
| 3 | Angela_1 | 0.7349 | 0.1480 | | 171 | | CRM_9 | | 0.7605 | 0.0920 |
| 7 | Angela_2 | 0.6601 | 0.2330 | | 152 | | Ogre_1 | | 0.6782 | 0.2880 |
| 11 | Angela_3 | 0.7251 | 0.1630 | | 184 | | Ogre_2 | | 0.4720 | 0.5490 |
| 21 | Angela_4 | 0.5692 | 0.3330 | | 2 | | Retand_1 | | 0.3831 | 0.7980 |
| 26 | Angela_5 | 0.5260 | 0.4440 | | 4 | | Retand_2 | | 0.4944 | 0.5290 |
| 30 | Angela_6 | 0.5605 | 0.3710 | | 5 | | Retand_3 | | 0.5164 | 0.4030 |
| 35 | Angela_7 | 0.5329 | 0.3950 | | 14 | | Retand_4 | | 0.4230 | 0.7090 |
| 41 | Angela_8 | 0.7546 | 0.1210 | | 19 | | Retand_5 | | 0.6771 | 0.1810 |
| 48 | Angela_9 | 0.7401 | 0.1350 | | 37 | | Retand_6 | | 0.3956 | 0.7640 |
| 75 | **Angela_10** | **0.8110** | **0.0830** | | 89 | | Retand_7 | | 0.5288 | 0.4010 |
| 76 | Angela_11 | 0.4887 | 0.5130 | | 193 | | Retand_8 | | 0.7818 | 0.1380 |
| 78 | Angela_12 | 0.5210 | 0.3690 | | 23 | | Tekay_1 | | 0.5026 | 0.5260 |
| 117 | Angela_13 | 0.5121 | 0.4270 | | 85 | | **Tekay_2** | | **1.1647** | **0.0900** |
| 120 | Angela_14 | 0.4530 | 0.6090 | | 97 | | **Tekay_3** | | **1.2022** | **0.0780** |
| 153 | Angela_15 | 0.4987 | 0.4770 | |  | |  | |  |  |

(D) Schedonorus

| **N. Cluster** | **Cluster**  **Name** | **Phylogenetic signal on combined tree** | | **N. Cluster** | | **Cluster**  **Name** | **Phylogenetic signal on combined tree** | |
| --- | --- | --- | --- | --- | --- | --- | --- | --- |
|  |  | **K** | **p-value** |  |  | | **K** | **p-value** |
| 57 | LINE_1 | 0.3705 | 0.3440 | 139 | SIRE_10 | | 0.1483 | 0.9960 |
| 157 | LINE_2 | 0.2186 | 0.8270 | 67 | TAR | | 0.1890 | 0.9090 |
| 2 | **LTR_1** | **1.5842** | **0.0020** | 183 | Tork | | 0.2287 | 0.8030 |
| 12 | LTR_2 | 0.3209 | 0.5250 | 11 | Athila_1 | | 0.5269 | 0.1640 |
| 27 | **LTR_3** | **1.5812** | **0.0010** | 26 | Athila_2 | | 0.4200 | 0.3390 |
| 56 | **LTR_4** | **1.4556** | **0.0010** | 29 | Athila_3 | | 0.3408 | 0.5330 |
| 64 | **LTR_5** | **0.8215** | **0.0210** | 31 | Athila_4 | | 0.2637 | 0.7040 |
| 71 | **LTR_6** | **1.2780** | **0.0010** | 33 | Athila_5 | | 0.4679 | 0.2820 |
| 79 | **LTR_7** | **1.6481** | **0.0010** | 42 | Athila_6 | | 0.6943 | 0.0380 |
| 113 | **LTR_8** | **1.5144** | **0.0010** | 44 | Athila_7 | | 0.3594 | 0.4870 |
| 149 | **LTR_9** | **1.3891** | **0.0020** | 45 | Athila_8 | | 0.1765 | 0.9040 |
| 158 | **LTR_10** | **1.4747** | **0.0020** | 47 | Athila_9 | | 1.5761 | 0.0010 |
| 187 | LTR_11 | 0.2718 | 0.6820 | 48 | Athila_10 | | 0.2335 | 0.7880 |
| 82 | 45S_rDNAS1 | 0.6840 | 0.0570 | 54 | Athila_11 | | 0.3823 | 0.4270 |
| 93 | 45S_rDNA_2 | 0.5688 | 0.1450 | 59 | **Athila_12** | | **1.5937** | **0.0010** |
| 127 | 5S_rDNA | 0.3046 | 0.5960 | 60 | Athila_13 | | 0.2908 | 0.6600 |
| 83 | **Repeat_1** | **0.8499** | **0.0050** | 65 | Athila_14 | | 0.7097 | 0.0500 |
| 99 | Repeat_2 | 0.5225 | 0.1030 | 76 | Athila_15 | | 0.1966 | 0.8560 |
| 105 | **Repeat_3** | **1.1762** | **0.0010** | 78 | Athila_16 | | 0.2506 | 0.7410 |
| 116 | Repeat_4 | 0.4963 | 0.2720 | 80 | Athila_17 | | 0.2559 | 0.7610 |
| 130 | **Repeat_5** | **1.1488** | **0.0010** | 124 | Athila_18 | | 0.5208 | 0.1130 |
| 1 | Satellite_1 | 0.7178 | 0.0290 | 190 | Athila_19 | | 0.2890 | 0.6350 |
| 9 | Satellite_2 | 0.3350 | 0.5370 | 7 | CRM_1 | | 0.4637 | 0.1690 |
| 37 | Satellite_3 | 0.5394 | 0.1320 | 30 | CRM_2 | | 0.2489 | 0.7650 |
| 39 | Satellite_4 | 0.3527 | 0.4060 | 41 | CRM_3 | | 0.2486 | 0.7790 |
| 46 | Satellite_5 | 0.4842 | 0.1040 | 61 | CRM_4 | | 0.4670 | 0.1910 |
| 97 | Satellite_6 | 0.3450 | 0.5240 | 69 | CRM_5 | | 0.2767 | 0.6630 |
| 106 | Satellite_7 | 0.3020 | 0.6540 | 123 | CRM_6 | | 0.2620 | 0.7110 |
| 109 | Satellite_8 | 0.6066 | 0.0950 | 150 | CRM_7 | | 0.2515 | 0.7470 |
| 111 | Satellite_9 | 0.5399 | 0.2640 | 154 | **CRM_8** | | **0.8502** | **0.0050** |
| 126 | Satellite_10 | 0.6675 | 0.0420 | 175 | CRM_9 | | 0.5515 | 0.0930 |
| 133 | Satellite_11 | 0.4684 | 0.2770 | 4 | Retand_1 | | 0.2663 | 0.7290 |
| 171 | Satellite_12 | 0.4450 | 0.2800 | 8 | Retand_2 | | 0.3144 | 0.5930 |
| 179 | Satellite_13 | 0.3971 | 0.4150 | 10 | Retand_3 | | 0.3599 | 0.4780 |
| 185 | Satellite_14 | 0.6319 | 0.0940 | 17 | Retand_4 | | 0.3321 | 0.5640 |
| 186 | Satellite_15 | 0.5587 | 0.0530 | 18 | Retand_5 | | 0.2748 | 0.6990 |
| 22 | EnSpm_CACTA_1 | 0.1688 | 0.9490 | 19 | Retand_6 | | 0.2932 | 0.6330 |
| 32 | EnSpm_CACTA_2 | 0.2353 | 0.8030 | 21 | Retand_7 | | 0.3357 | 0.5110 |
| 87 | EnSpm_CACTA_3 | 0.4075 | 0.2460 | 24 | Retand_8 | | 0.2946 | 0.6320 |
| 135 | EnSpm_CACTA_4 | 0.3635 | 0.3550 | 38 | Retand_9 | | 0.2642 | 0.7020 |
| 136 | EnSpm_CACTA_5 | 0.4831 | 0.1960 | 49 | Retand_10 | | 0.3449 | 0.5200 |
| 137 | EnSpm_CACTA_6 | 0.1642 | 0.9480 | 53 | Retand_11 | | 0.2818 | 0.6370 |
| 203 | EnSpm_CACTA_7 | 0.2418 | 0.7860 | 58 | Retand_12 | | 0.3672 | 0.4280 |
| 148 | MuDR_Mutator_1 | 0.3375 | 0.4670 | 72 | Retand_13 | | 0.3412 | 0.5230 |
| 169 | **MuDR_Mutator_2** | **0.8000** | **0.0050** | 74 | Retand_14 | | 0.2868 | 0.6490 |
| 194 | **MuDR_Mutator_3** | **1.7396** | **0.0010** | 77 | Retand_15 | | 0.3579 | 0.3890 |
| 20 | PIF_Harbinger_1 | 0.7504 | 0.0140 | 84 | Retand_16 | | 0.3713 | 0.3760 |
| 141 | PIF_Harbinger_2 | 0.6238 | 0.0500 | 89 | Retand_17 | | 0.2949 | 0.6100 |
| 3 | Angela_1 | 0.3177 | 0.5200 | 101 | Retand_18 | | 0.2817 | 0.6850 |
| 5 | Angela_2 | 0.2702 | 0.6510 | 103 | Retand_19 | | 0.3941 | 0.3370 |
| 14 | Angela_3 | 0.5073 | 0.1180 | 112 | Retand_20 | | 0.4008 | 0.3000 |
| 15 | Angela_4 | 0.2170 | 0.8160 | 117 | Retand_21 | | 0.3912 | 0.3590 |
| 55 | Angela_5 | 0.2384 | 0.7530 | 132 | Retand_22 | | 0.3261 | 0.4750 |
| 81 | Angela_6 | 0.2473 | 0.7380 | 160 | Retand_23 | | 0.4118 | 0.3610 |
| 86 | Angela_7 | 0.2889 | 0.6420 | 178 | Retand_24 | | 0.5538 | 0.0470 |
| 92 | Angela_8 | 0.2993 | 0.6190 | 180 | Retand_25 | | 0.4416 | 0.1580 |
| 115 | Angela_9 | 0.2473 | 0.7680 | 223 | Retand_26 | | 0.4754 | 0.1160 |
| 167 | Ikeros | 0.4735 | 0.1360 | 23 | Tekay_1 | | 0.3267 | 0.4720 |
| 13 | SIRE_1 | 0.2846 | 0.6170 | 25 | Tekay_2 | | 0.4040 | 0.3530 |
| 16 | SIRE_2 | 0.3148 | 0.5330 | 50 | Tekay_3 | | 0.2894 | 0.5810 |
| 40 | SIRE_3 | 0.3128 | 0.5040 | 63 | **Tekay_4** | | **1.3463** | **0.0040** |
| 43 | SIRE_4 | 0.3197 | 0.5320 | 90 | Tekay_5 | | 0.1691 | 0.9050 |
| 52 | SIRE_5 | 0.2477 | 0.7700 | 108 | Tekay_6 | | 0.4487 | 0.2940 |
| 68 | SIRE_6 | 0.2165 | 0.8290 | 118 | Tekay_7 | | 0.4218 | 0.3150 |
| 95 | SIRE_7 | 0.3357 | 0.4620 | 129 | Tekay_8 | | 0.2556 | 0.7410 |
| 98 | SIRE_8 | 0.3081 | 0.5600 | 220 | Tekay_9 | | 0.5207 | 0.1700 |
| 122 | SIRE_9 | 0.3181 | 0.5200 |  |  | |  |  |
