# Supplementary material for: Evolutionary Dynamics of the Repeatome Explains Contrasting Differences in Genome Sizes and Hybrid and Polyploid Origins of Grass Loliinae Lineages
Source: Front Plant Sci. 2022 Jul 1;13:901733. doi: 10.3389/fpls.2022.901733 (PMC9284676; doi:10.3389/fpls.2022.901733)
Supplement: Supplementary Figure 1 — (A) Combined (plastome + 35S rDNA) Loliinae coalescent species tree computed through Singular Value Decomposition quartets (SVDq) analysis showing bootstrap support values on branches. (B–D) Maximum Likelihood phylogenomic trees of 47 Loliinae samples based on (B) Combined (plastome + 35S rDNA) data, (C) plastome data, (D) nuclear 35S rDNA data, (E) Histograms of repeat contents per holoploid genome (1C) retrieved from the individual Repeat Explorer 2 analyses of the studied Loliinae samples mapped onto the Maximum Likelihood combined phylogenomic tree (plastome + nuclear 35S rDNA cistron) of Loliinae. Ultrafast bootstrap support values are indicated on branches. Oryza sativa and Brachypodium distachyon outgroups were used to root the trees. Color codes of Loliinae lineages are indicated in the charts. Scale bar: number of mutations per site. [file Data_Sheet_1.PDF]

Suppl. Fig S1A

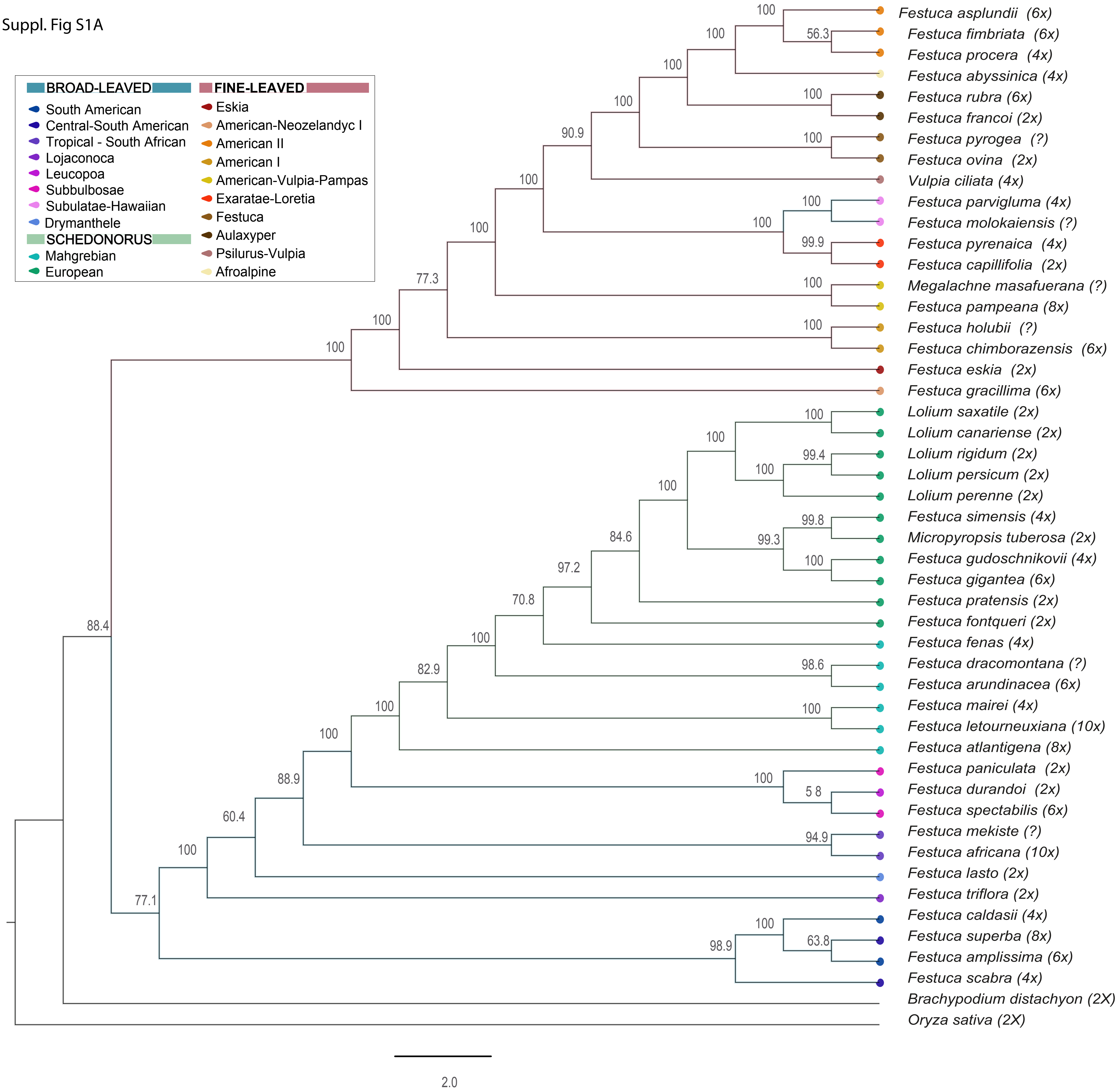

Suppl. Fig S1B

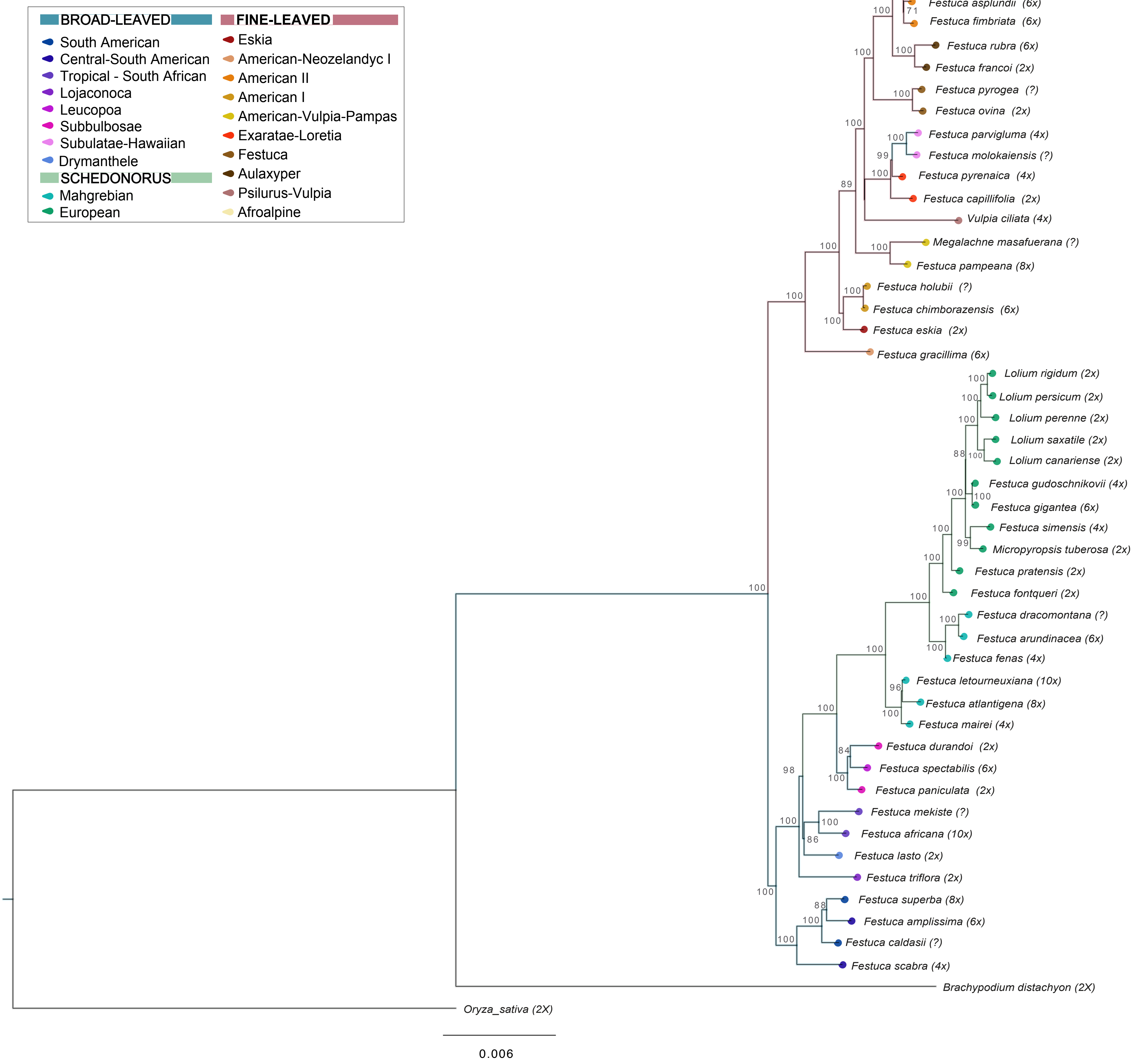

Suppl. Fig S1C

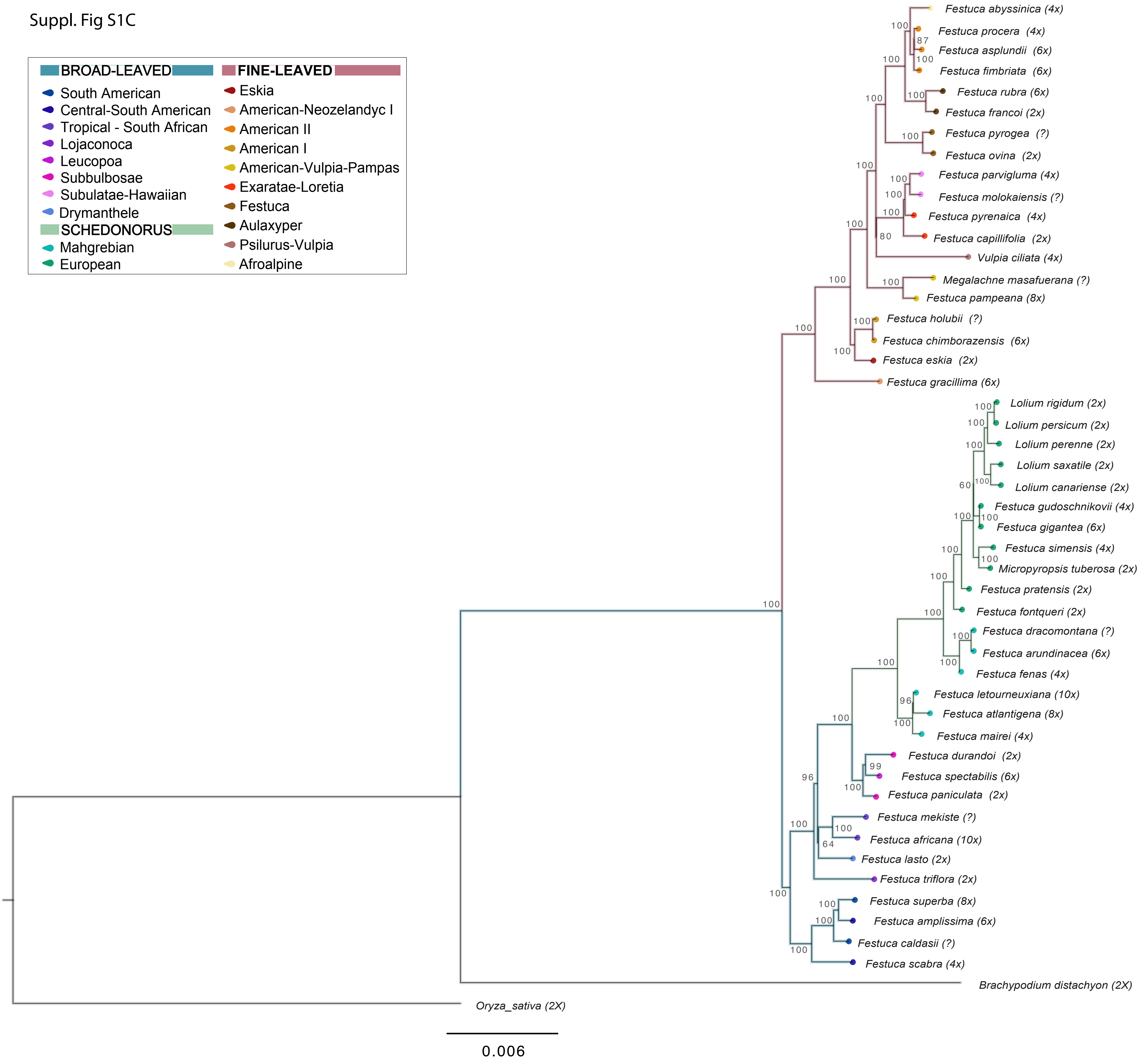

Suppl. Fig S1D

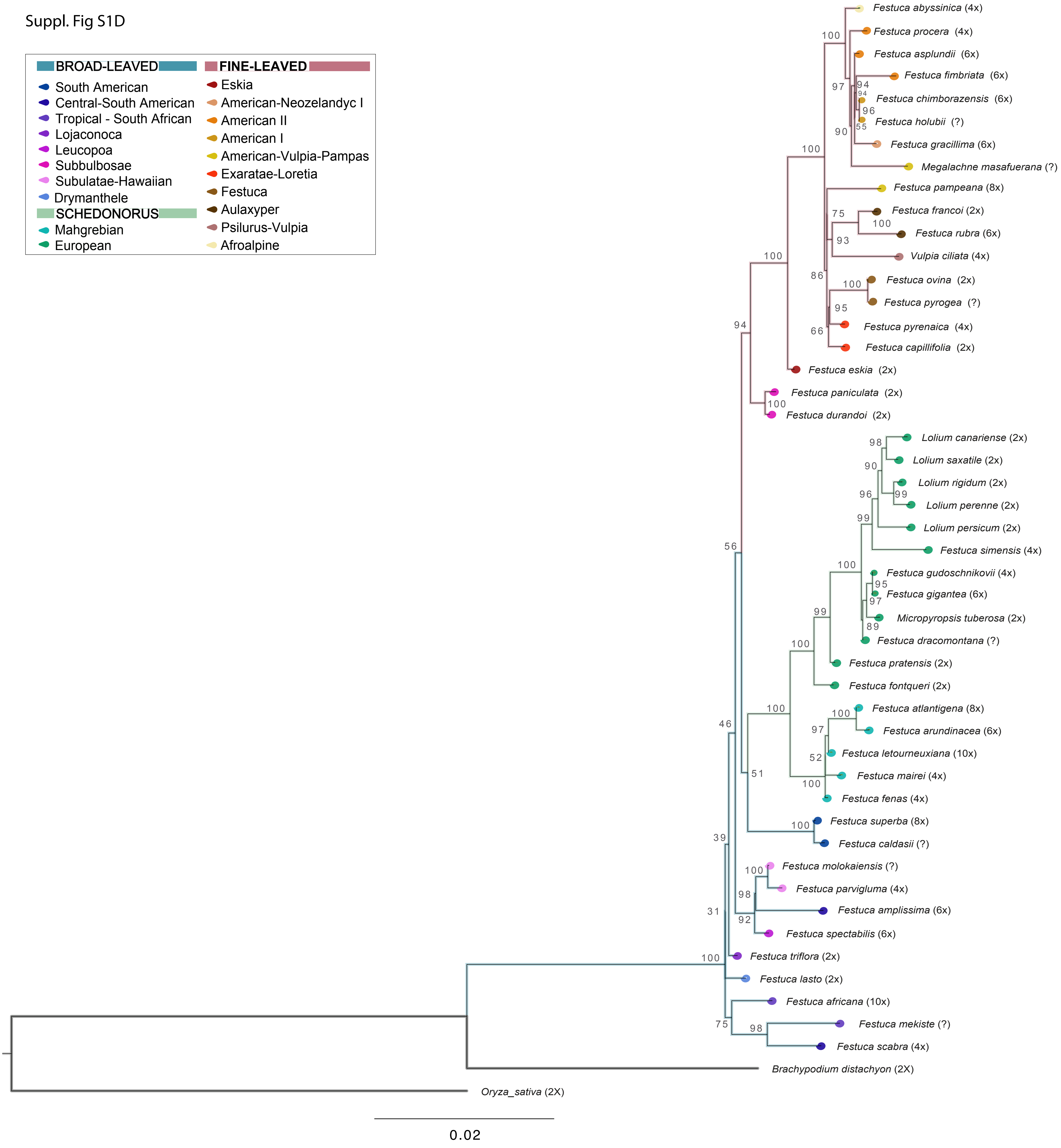

Suppl. Fig S1E

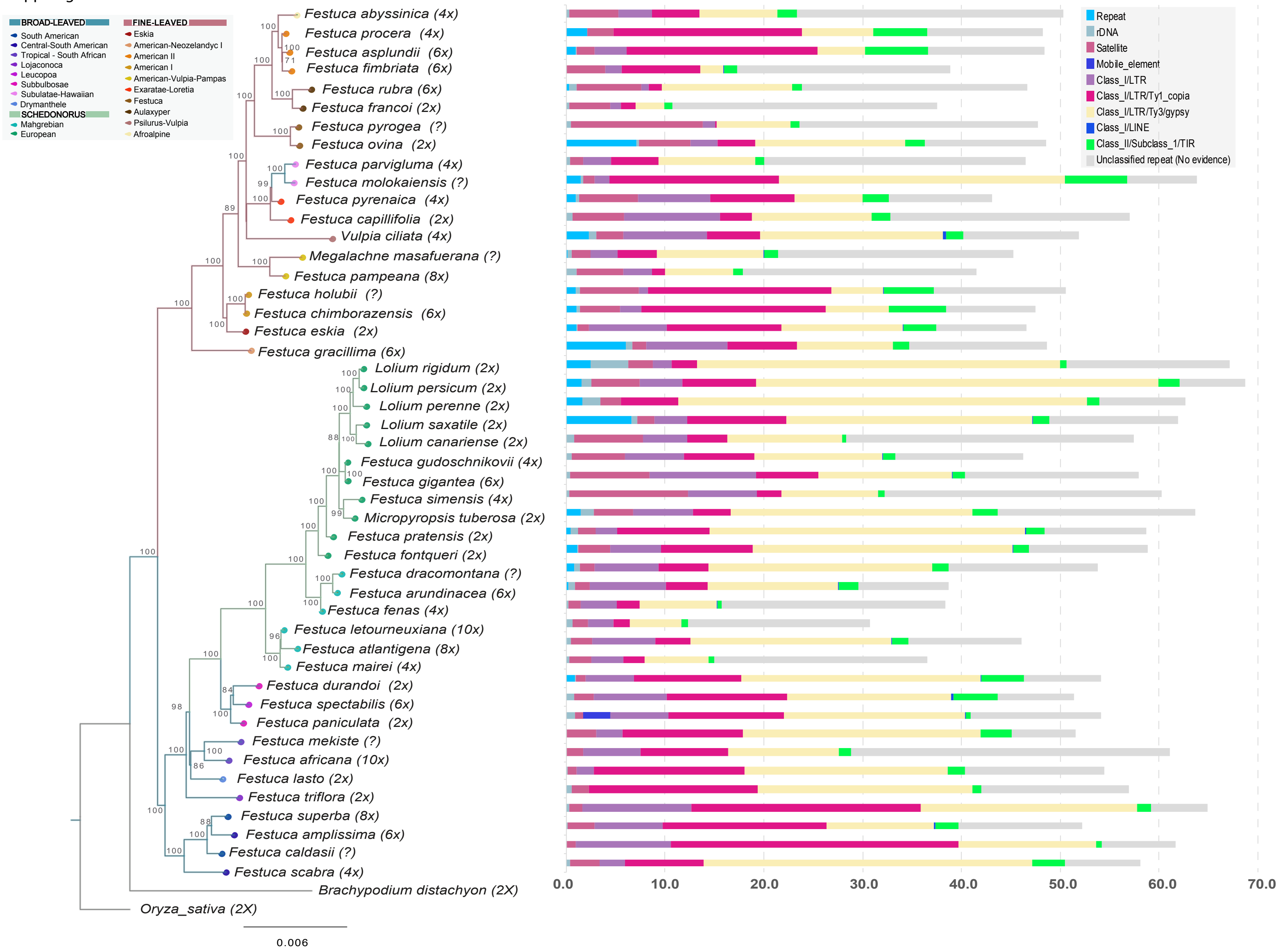

Suppl. Fig. S2

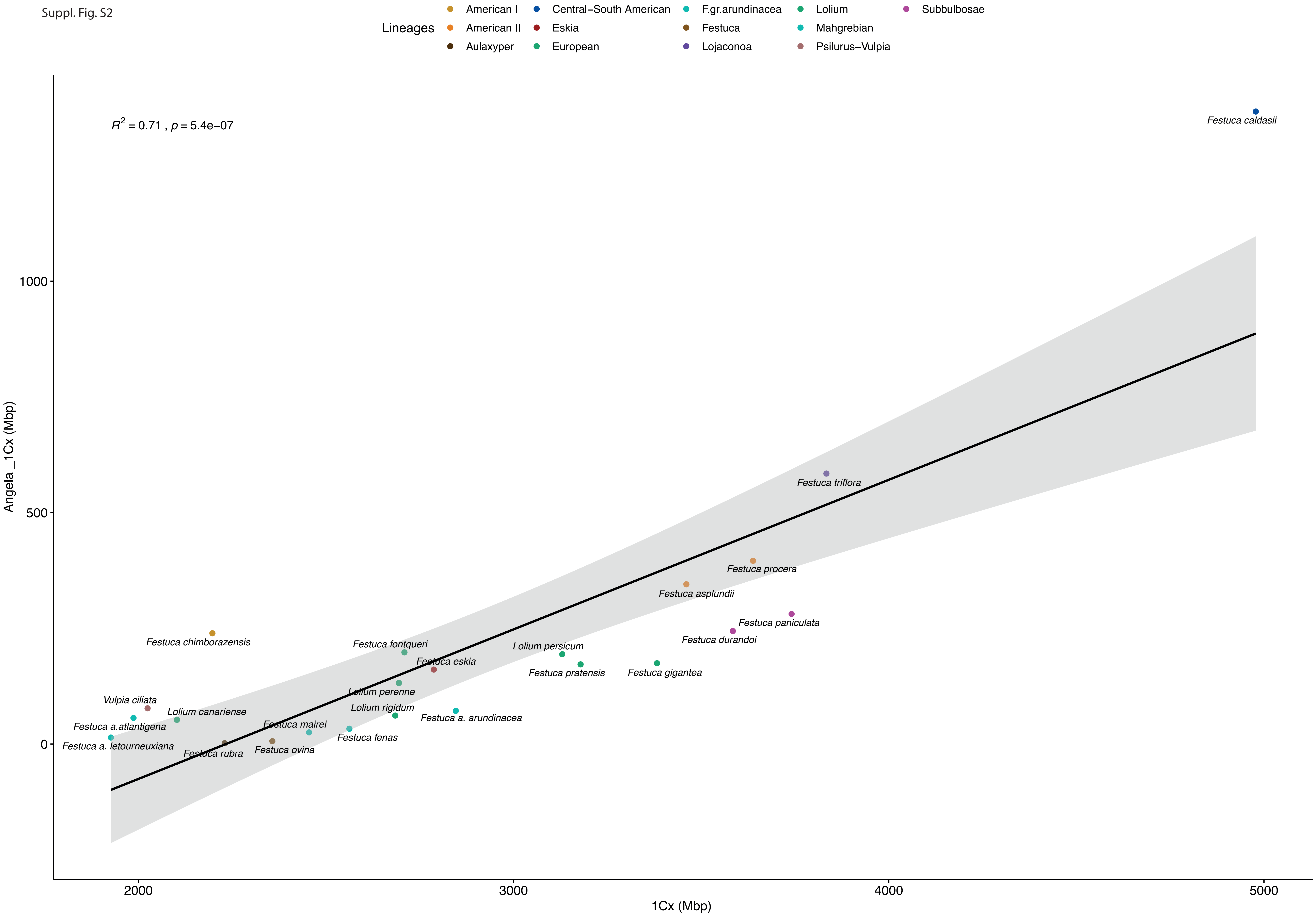
Lineages
American I
American II
Aulaxyper
Central–South American
Eskia
European
F.gr.arundinacea
Festuca
Lojaconoa
Lolium
Mahgrebian
Psilurus–Vulpia
Subbulbosae
R^2 = 0.71 , p = 5.4e–07
Festuca caldasii
Festuca triflora
Festuca procera
Festuca asplundii
Festuca paniculata
Festuca durandoi
Festuca chimborazensis
Festuca fontqueri
Festuca eskia
Lolium persicum
Festuca pratensis
Festuca gigantea
Lolium perenne
Vulpia ciliata
Lolium canariense
Lolium rigidum
Festuca a. arundinacea
Festuca a.atlantigena
Festuca mairei
Festuca fenas
Festuca a. letourneuxiana
Festuca rubra
Festuca ovina
Angela _1Cx (Mbp)
1000
500
0
2000
3000
4000
5000
1Cx (Mbp)

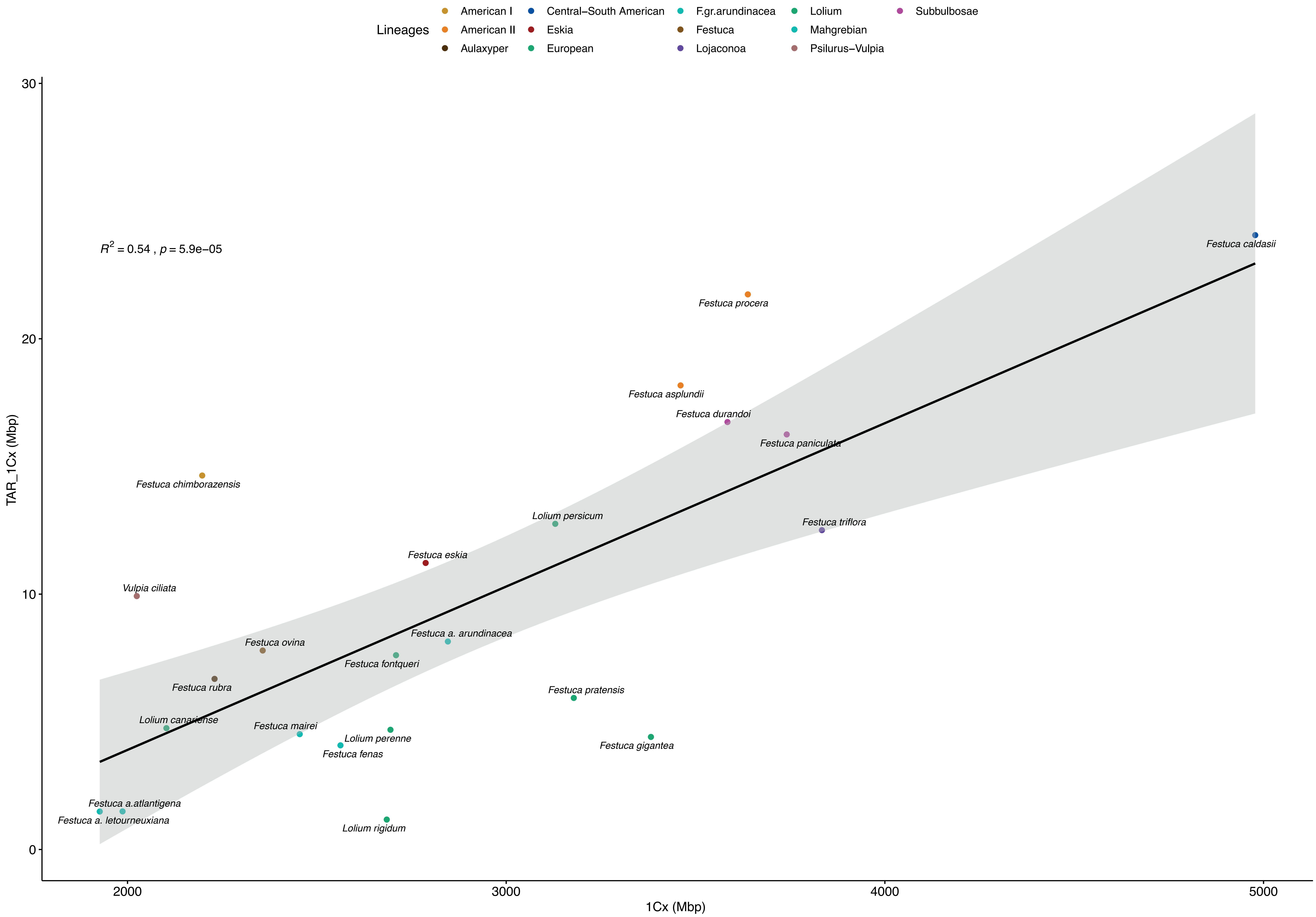

Lineages
American I
American II
Aulaxyper
Central–South American
Eskia
European
F.gr.arundinacea
Festuca
Lojaconoa
Lolium
Mahgrebian
Psilurus–Vulpia
Subbulbosae
$R^2 = 0.54$, $p = 5.9e-05$
TAR_1Cx (Mbp)
1Cx (Mbp)
0
10
20
30
2000
3000
4000
5000
Festuca caldasii
Festuca procera
Festuca asplundii
Festuca durandoi
Festuca paniculata
Festuca chimborazensis
Lolium persicum
Festuca triflora
Festuca eskia
Vulpia ciliata
Festuca a. arundinacea
Festuca ovina
Festuca fontqueri
Festuca rubra
Festuca pratensis
Lolium canariense
Festuca mairei
Lolium perenne
Festuca fenas
Festuca gigantea
Festuca a.atlantigena
Festuca a. letourneuxiana
Lolium rigidum

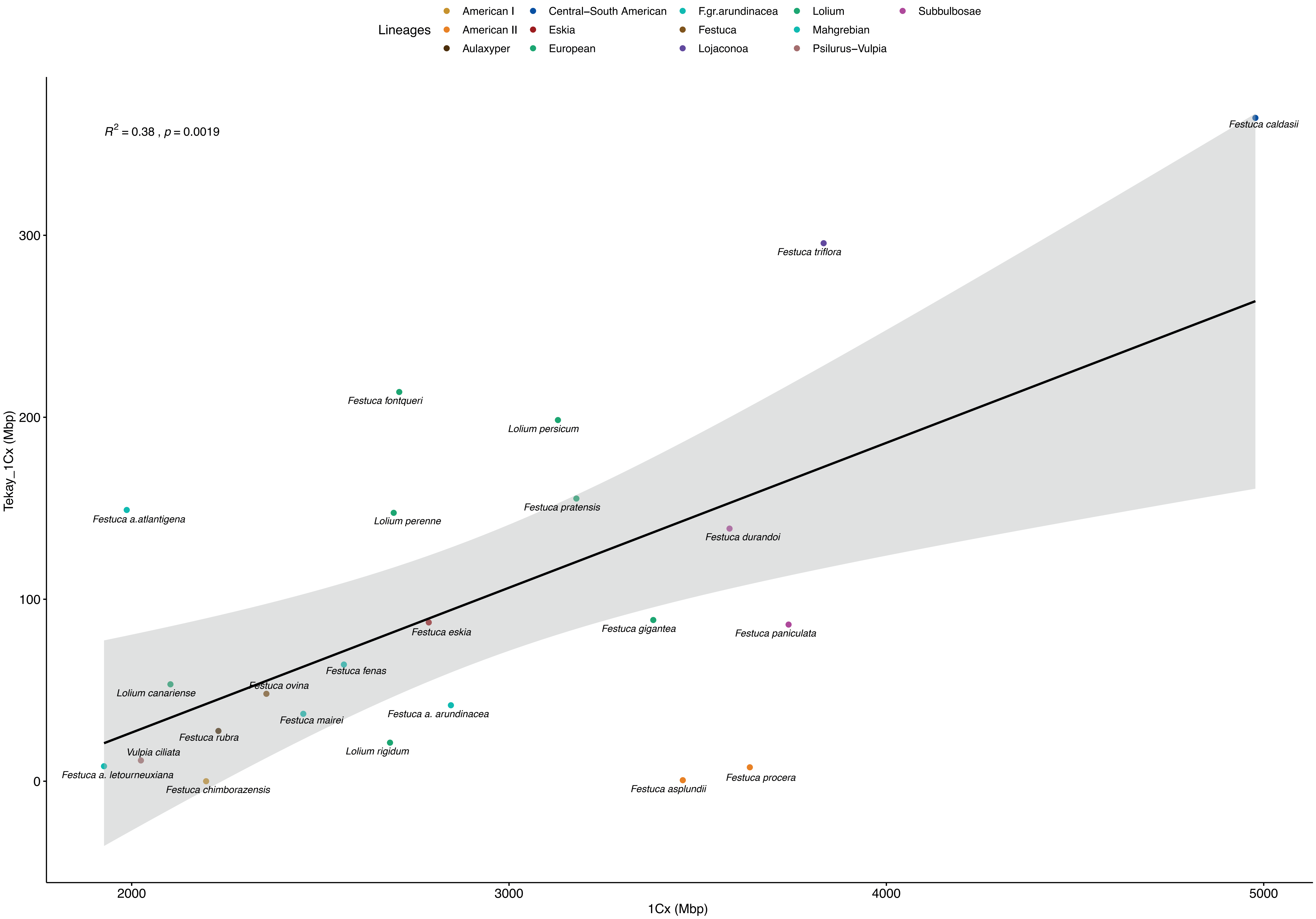

Lineages
American I
American II
Aulaxyper
Central–South American
Eskia
European
F.gr.arundinacea
Festuca
Lojaconoa
Lolium
Mahgrebian
Psilurus–Vulpia
Subbulbosae
$R^2 = 0.38$, $p = 0.0019$
Tekay_1Cx (Mbp)
1Cx (Mbp)
0
100
200
300
2000
3000
4000
5000
Festuca caldasii
Festuca triflora
Festuca fontqueri
Lolium persicum
Festuca pratensis
Festuca a.atlantigena
Lolium perenne
Festuca durandoi
Festuca eskia
Festuca gigantea
Festuca paniculata
Festuca fenas
Lolium canariense
Festuca ovina
Festuca mairei
Festuca a. arundinacea
Festuca rubra
Lolium rigidum
Vulpia ciliata
Festuca a. letourneuxiana
Festuca chimborazensis
Festuca asplundii
Festuca procera

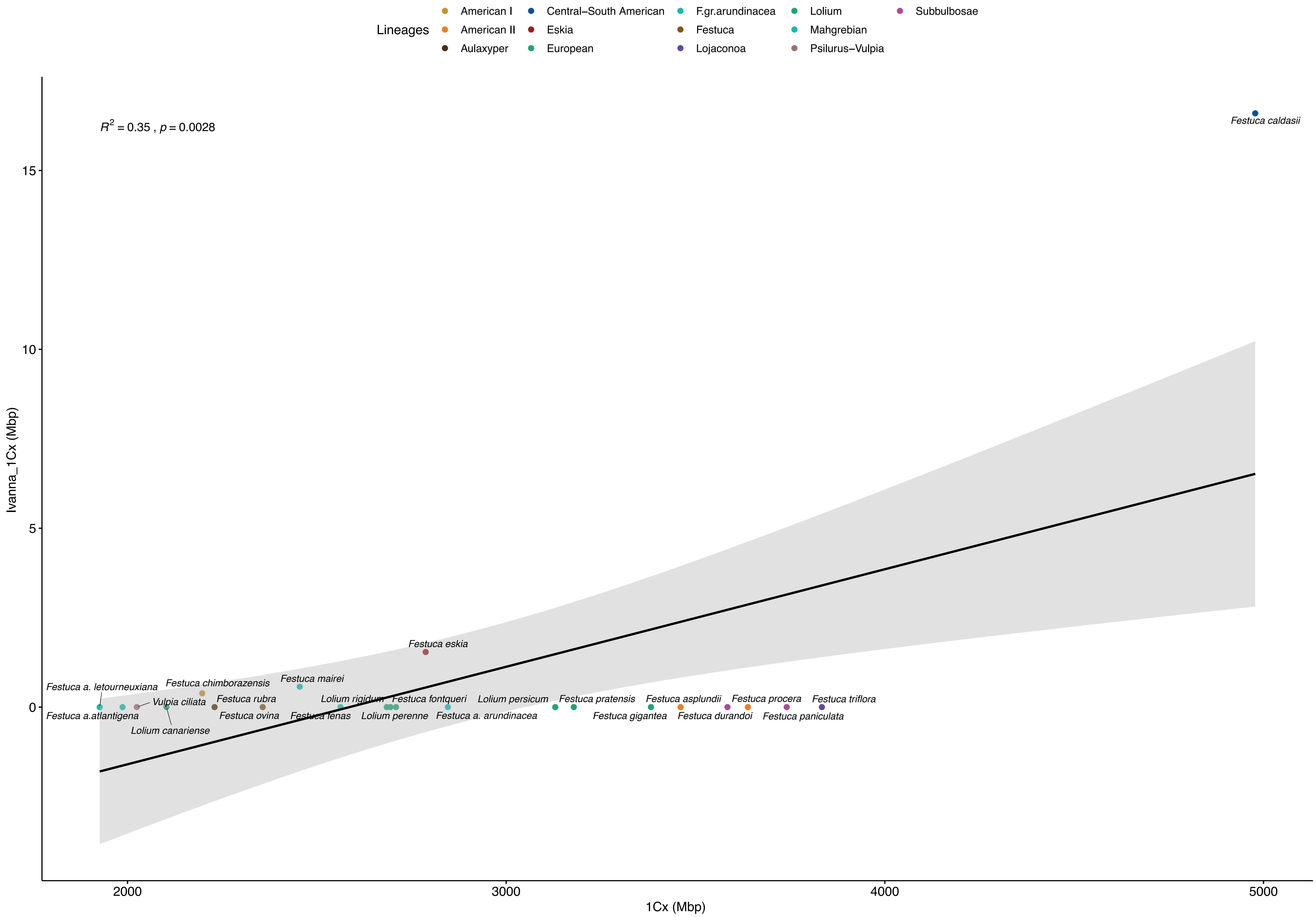

Lineages
American I
American II
Aulaxyper
Central–South American
Eskia
European
F.gr.arundinacea
Festuca
Lojaconoa
Lolium
Mahgrebian
Psilurus–Vulpia
Subbulbosae
$R^2 = 0.35$, $p = 0.0028$
Festuca caldasii
Festuca eskia
Festuca a. letourneuxiana
Festuca chimborazensis
Festuca mairei
Vulpia ciliata
Festuca rubra
Lolium rigidum
Festuca fontqueri
Lolium persicum
Festuca pratensis
Festuca asplundii
Festuca procera
Festuca triflora
Festuca a.atlantigena
Festuca ovina
Festuca lenas
Lolium perenne
Festuca a. arundinacea
Festuca gigantea
Festuca durandoi
Festuca paniculata
Lolium canariense
Ivanna_1Cx (Mbp)
1Cx (Mbp)
0
5
10
15
2000
3000
4000
5000

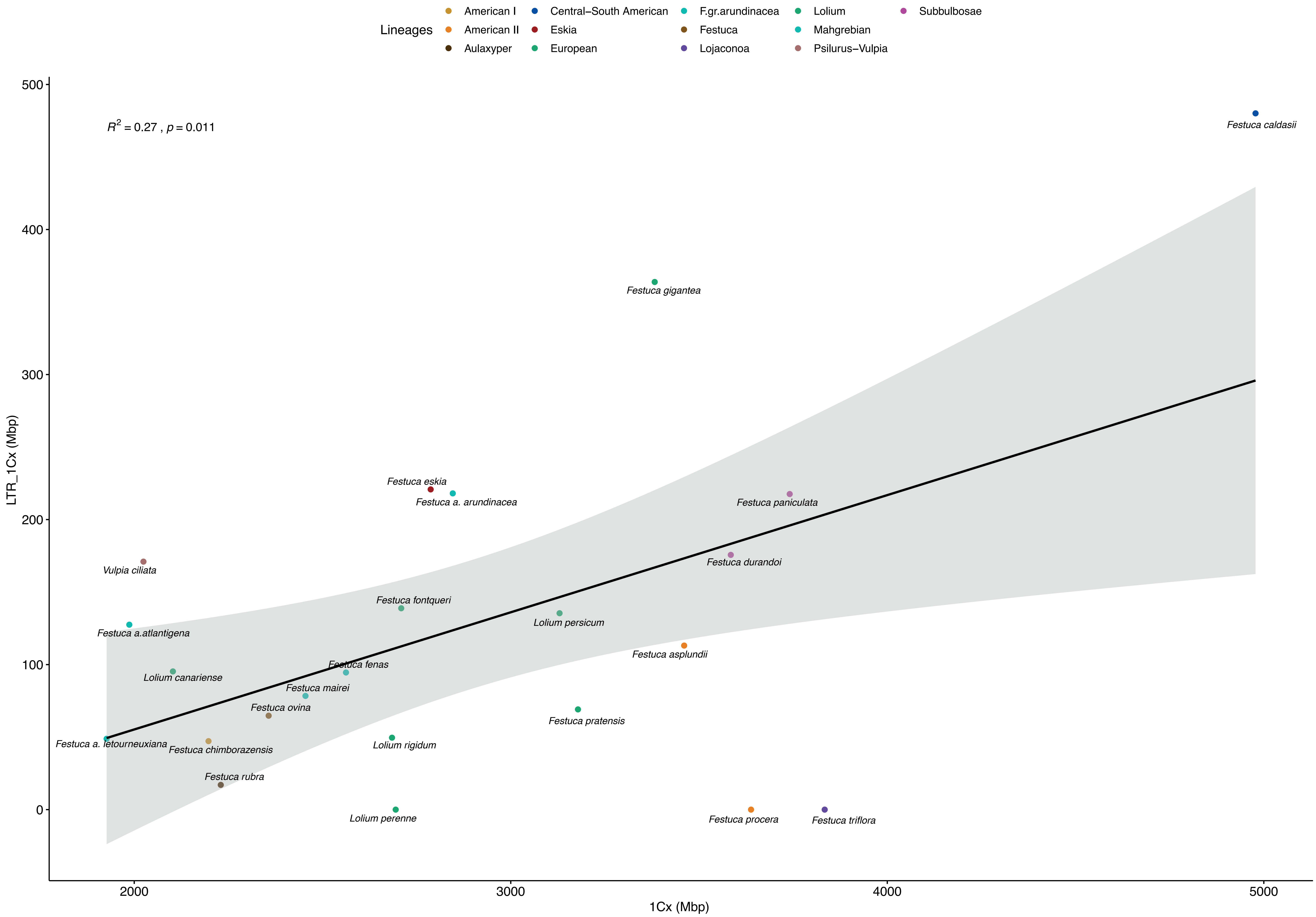

Lineages
American I
American II
Aulaxyper
Central–South American
Eskia
European
F.gr.arundinacea
Festuca
Lojaconoa
Lolium
Mahgrebian
Psilurus–Vulpia
Subbulbosae
$R^2 = 0.27$, $p = 0.011$
Festuca caldasii
Festuca gigantea
Festuca eskia
Festuca a. arundinacea
Festuca paniculata
Vulpia ciliata
Festuca durandoi
Festuca fontqueri
Lolium persicum
Festuca a.atlantigena
Festuca asplundii
Festuca fenas
Lolium canariense
Festuca mairei
Festuca ovina
Festuca pratensis
Festuca a. letourneuxiana
Festuca chimborazensis
Lolium rigidum
Festuca rubra
Lolium perenne
Festuca procera
Festuca triflora
LTR_1Cx (Mbp)
1Cx (Mbp)
0
100
200
300
400
500
2000
3000
4000
5000

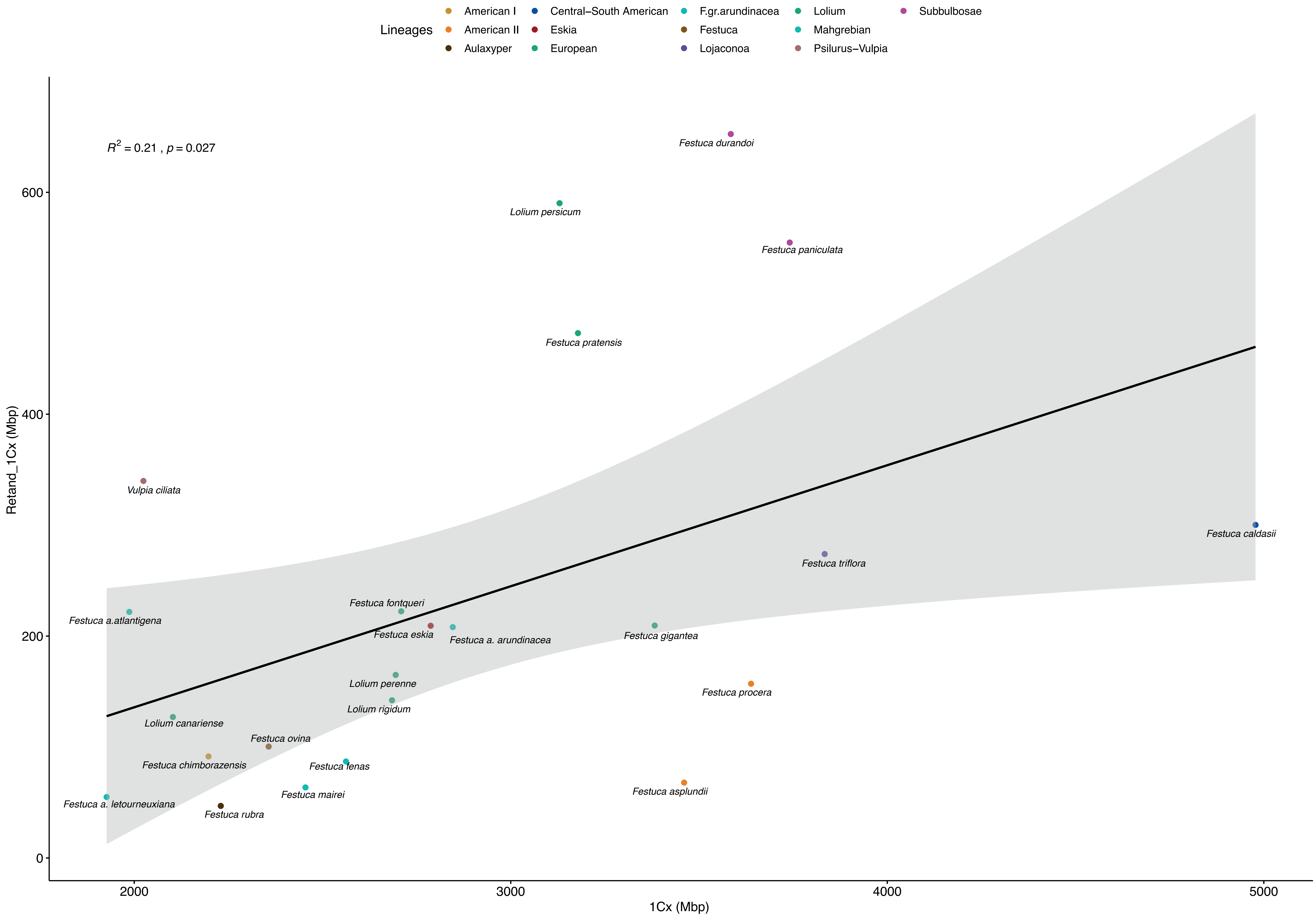
Lineages
American I
American II
Aulaxyper
Central–South American
Eskia
European
F.gr.arundinacea
Festuca
Lojaconoa
Lolium
Mahgrebian
Psilurus–Vulpia
Subbulbosae
$R^2 = 0.21$, $p = 0.027$
Festuca durandoi
Lolium persicum
Festuca paniculata
Festuca pratensis
Vulpia ciliata
Festuca caldasii
Festuca triflora
Festuca fontqueri
Festuca a.atlantigena
Festuca eskia
Festuca a. arundinacea
Festuca gigantea
Lolium perenne
Festuca procera
Lolium rigidum
Lolium canariense
Festuca ovina
Festuca chimborazensis
Festuca lenas
Festuca mairei
Festuca asplundii
Festuca a. letourneuxiana
Festuca rubra
Retand_1Cx (Mbp)
0
200
400
600
2000
3000
4000
5000
1Cx (Mbp)

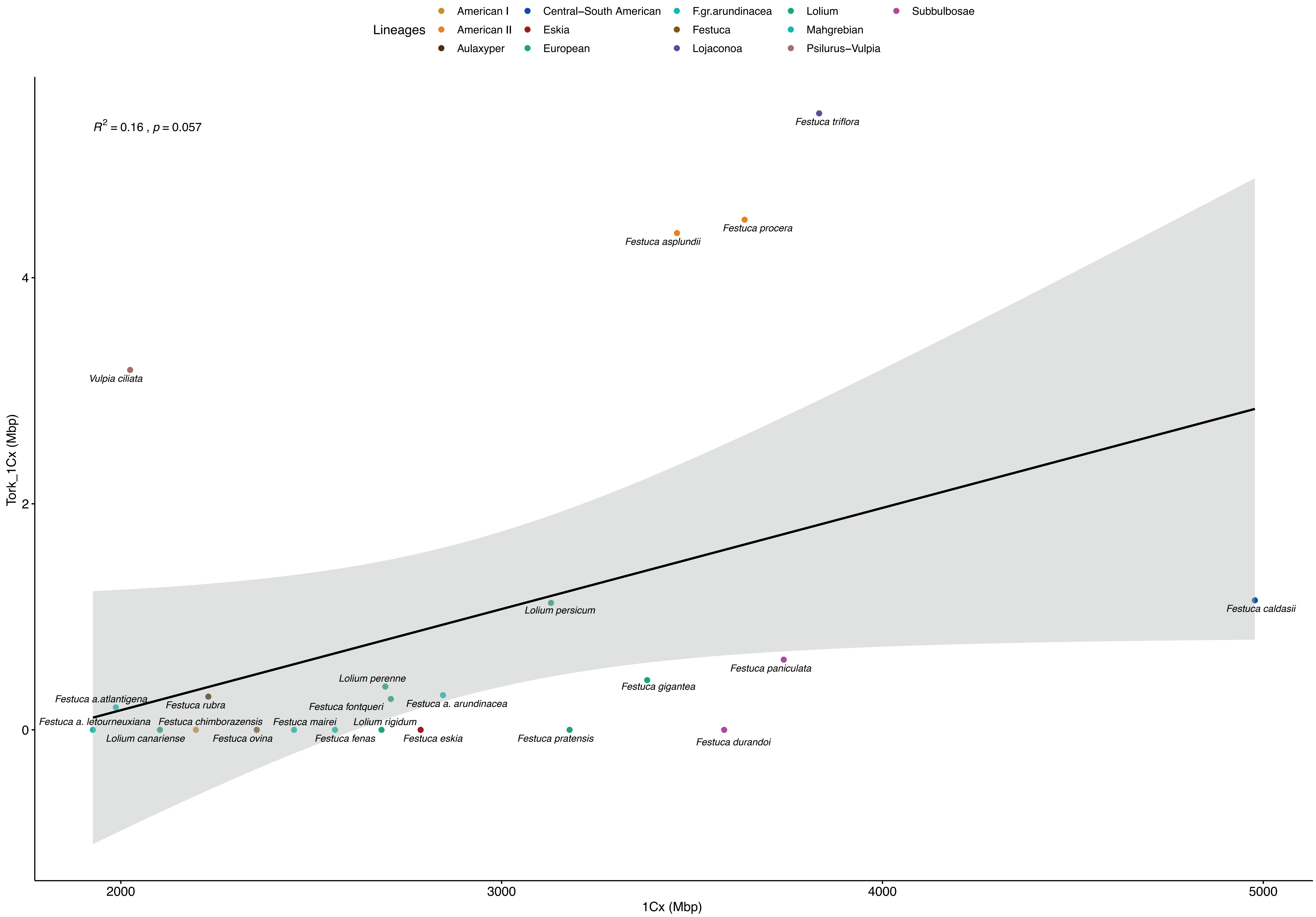

Lineages
American I
American II
Aulaxyper
Central–South American
Eskia
European
F.gr.arundinacea
Festuca
Lojaconoa
Lolium
Mahgrebian
Psilurus–Vulpia
Subbulbosae
$R^2 = 0.16$, $p = 0.057$
Festuca triflora
Festuca procera
Festuca asplundii
Vulpia ciliata
Lolium persicum
Festuca caldasii
Festuca paniculata
Festuca gigantea
Lolium perenne
Festuca a.atlantigena
Festuca rubra
Festuca fontqueri
Festuca a. arundinacea
Festuca a. letourneuxiana
Festuca chimborazensis
Festuca mairei
Lolium rigidum
Lolium canariense
Festuca ovina
Festuca fenas
Festuca eskia
Festuca pratensis
Festuca durandoi
Tork_1Cx (Mbp)
1Cx (Mbp)
0
2
4
2000
3000
4000
5000

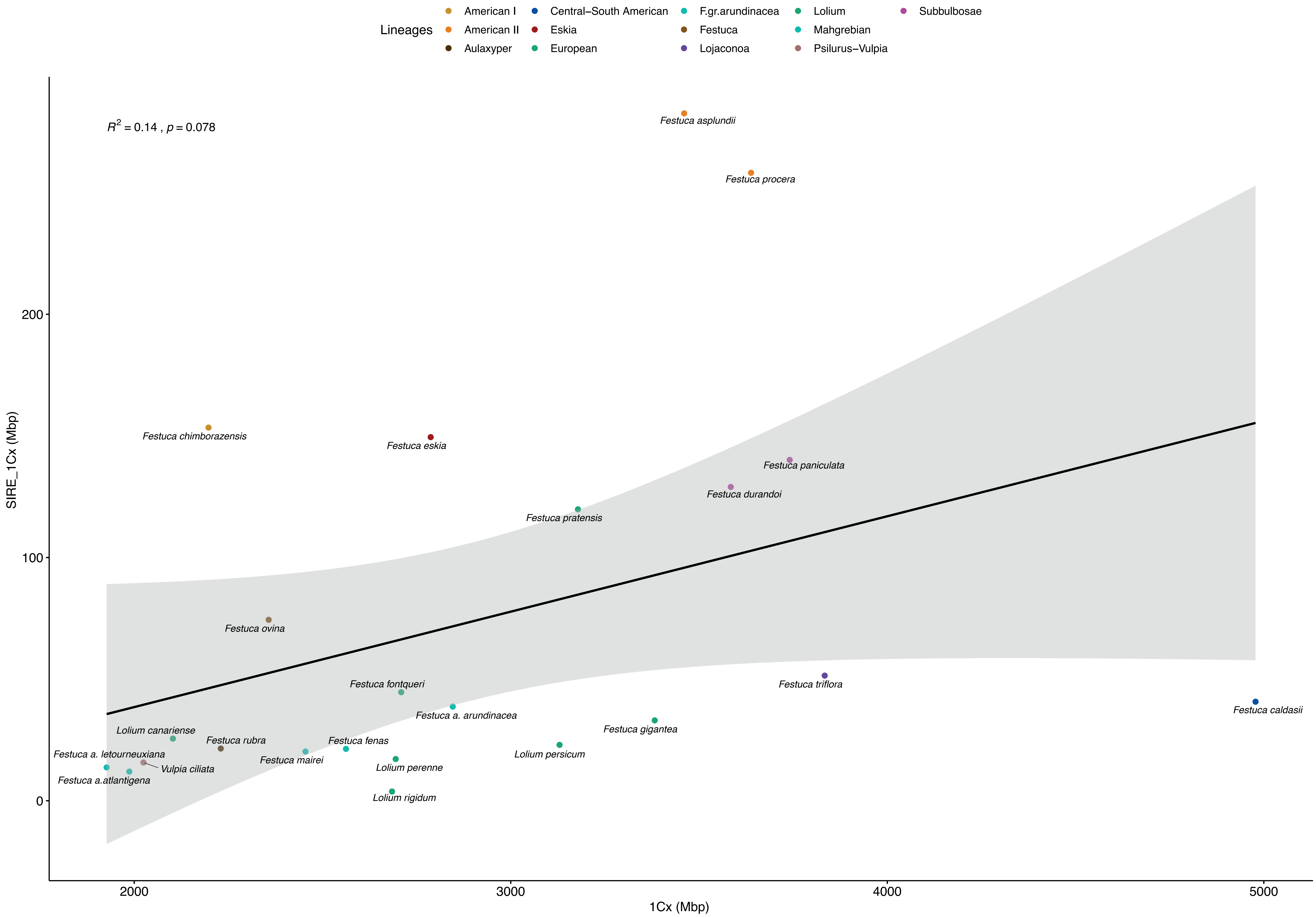

Lineages
American I
American II
Aulaxyper
Central–South American
Eskia
European
F.gr.arundinacea
Festuca
Lojaconoa
Lolium
Mahgrebian
Psilurus–Vulpia
Subbulbosae
$R^2 = 0.14$, $p = 0.078$
Festuca asplundii
Festuca procera
Festuca chimborazensis
Festuca eskia
Festuca paniculata
Festuca durandoi
Festuca pratensis
Festuca ovina
Festuca triflora
Festuca caldasii
Festuca fontqueri
Festuca a. arundinacea
Festuca gigantea
Lolium canariense
Festuca rubra
Festuca fenas
Festuca a. letourneuxiana
Festuca mairei
Lolium persicum
Vulpia ciliata
Lolium perenne
Festuca a.atlantigena
Lolium rigidum
SIRE_1Cx (Mbp)
1Cx (Mbp)
0
100
200
2000
3000
4000
5000

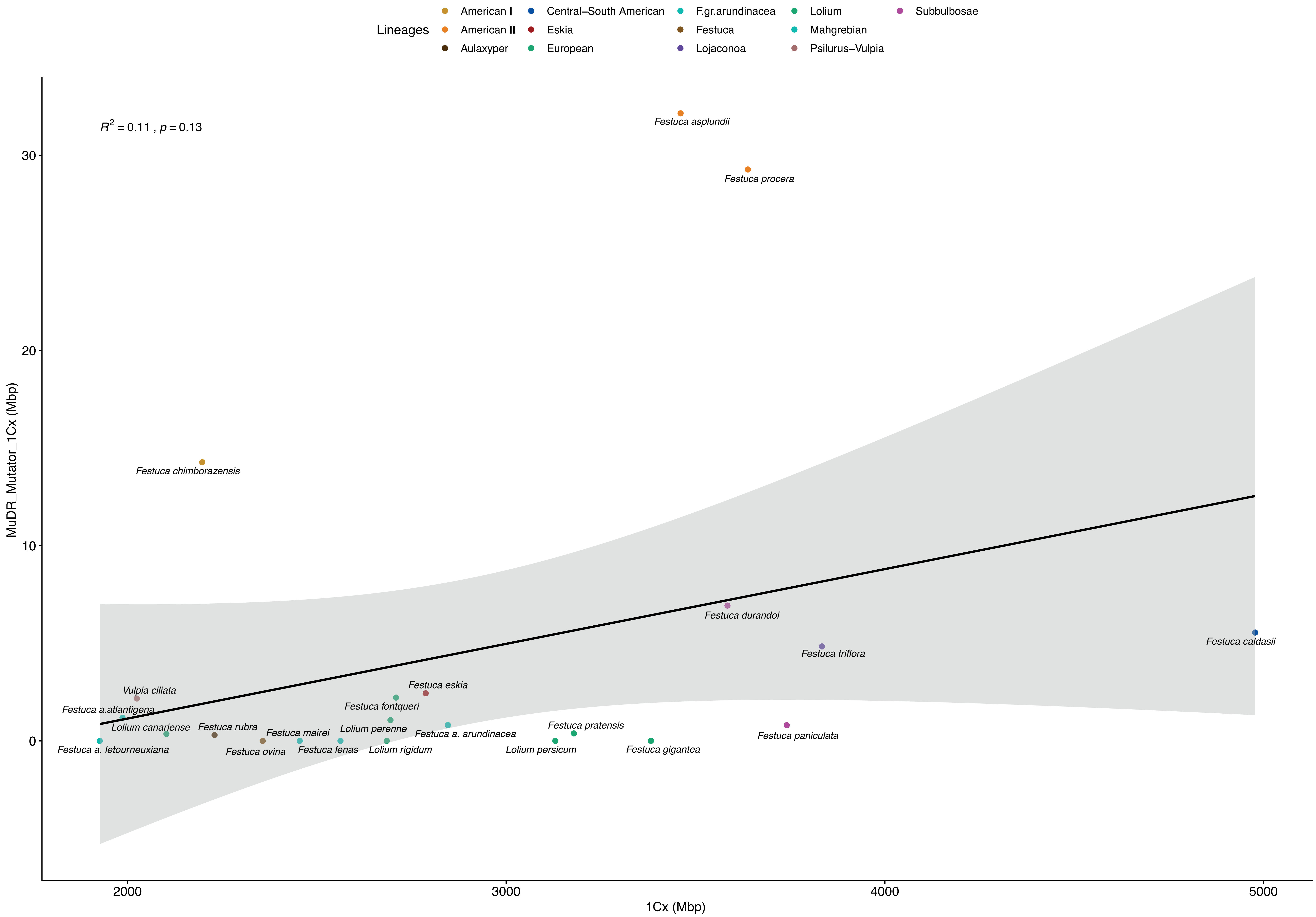
Lineages
American I
American II
Aulaxyper
Central–South American
Eskia
European
F.gr.arundinacea
Festuca
Lojaconoa
Lolium
Mahgrebian
Psilurus–Vulpia
Subbulbosae
$R^2 = 0.11$, $p = 0.13$
Festuca asplundii
Festuca procera
Festuca chimborazensis
Festuca durandoi
Festuca caldasii
Festuca triflora
Vulpia ciliata
Festuca eskia
Festuca fontqueri
Festuca a.atlantigena
Lolium canariense
Festuca rubra
Festuca mairei
Lolium perenne
Festuca a. arundinacea
Festuca pratensis
Festuca paniculata
Festuca a. letourneuxiana
Festuca ovina
Festuca fenas
Lolium rigidum
Lolium persicum
Festuca gigantea
MuDR_Mutator_1Cx (Mbp)
0
10
20
30
2000
3000
4000
5000
1Cx (Mbp)

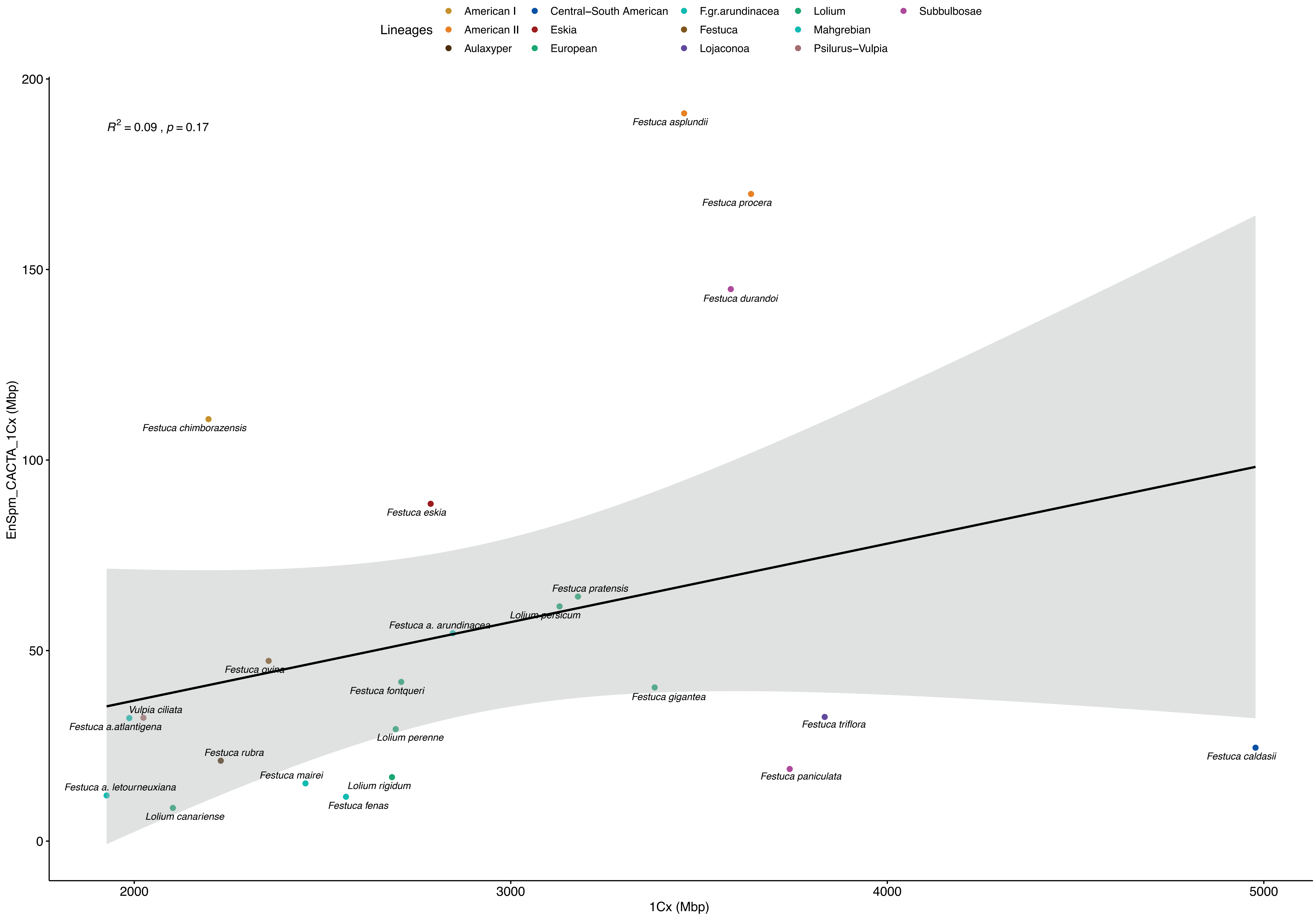

Lineages
American I
American II
Aulaxyper
Central−South American
Eskia
European
F.gr.arundinacea
Festuca
Lojaconoa
Lolium
Mahgrebian
Psilurus−Vulpia
Subbulbosae
$R^2 = 0.09$, $p = 0.17$
EnSpm_CACTA_1Cx (Mbp)
1Cx (Mbp)
200
150
100
50
0
2000
3000
4000
5000
Festuca asplundii
Festuca procera
Festuca durandoi
Festuca chimborazensis
Festuca eskia
Festuca pratensis
Lolium persicum
Festuca a. arundinacea
Festuca ovina
Festuca fontqueri
Festuca gigantea
Vulpia ciliata
Festuca a.atlantigena
Festuca triflora
Lolium perenne
Festuca caldasii
Festuca rubra
Festuca paniculata
Festuca mairei
Lolium rigidum
Festuca a. letourneuxiana
Festuca fenas
Lolium canariense

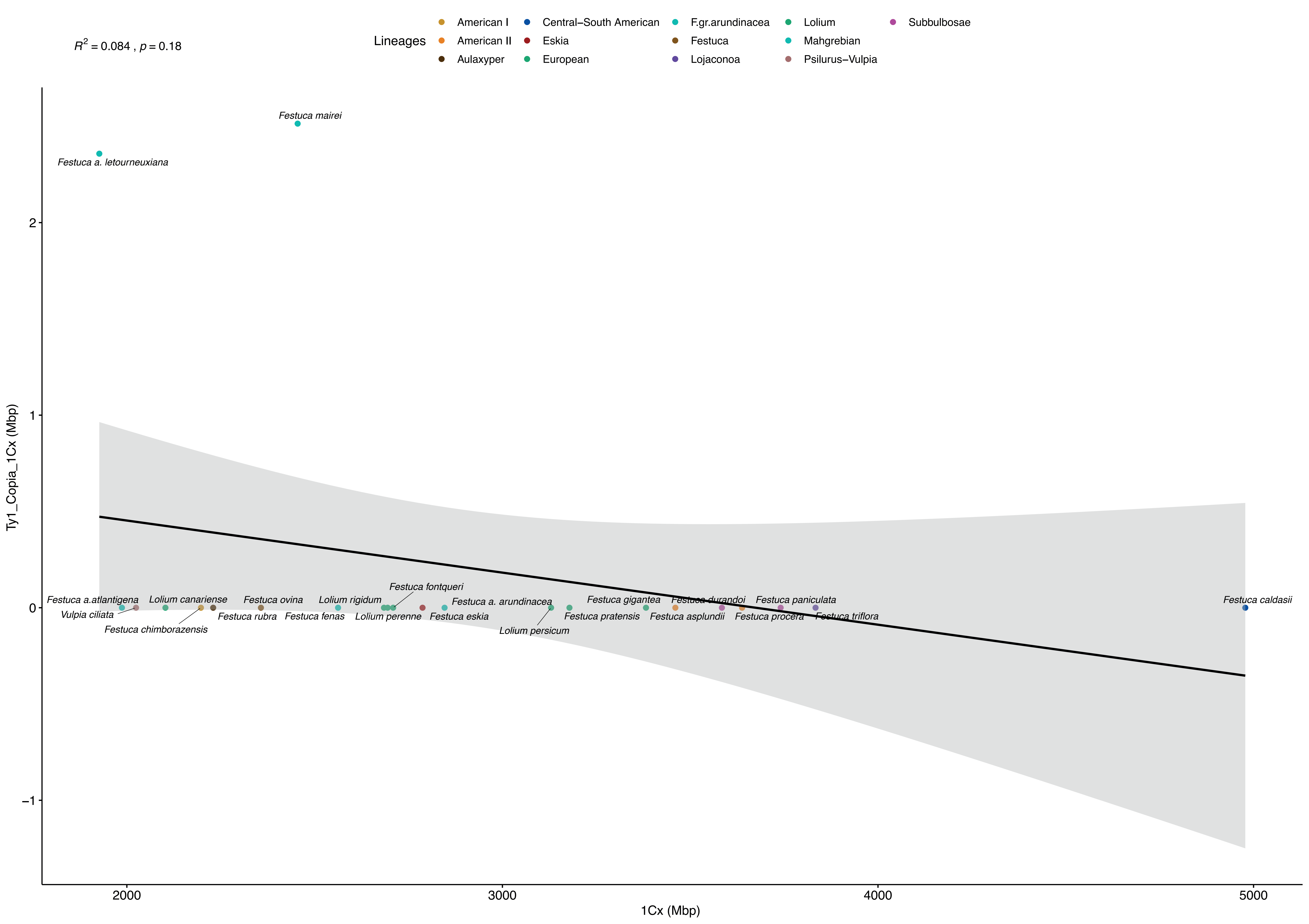

$R^2 = 0.084$, $p = 0.18$
Lineages
American I
American II
Aulaxyper
Central–South American
Eskia
European
F.gr.arundinacea
Festuca
Lojaconoa
Lolium
Mahgrebian
Psilurus–Vulpia
Subbulbosae
Festuca mairei
Festuca a. letourneuxiana
Festuca fontqueri
Festuca a.atlantigena
Lolium canariense
Festuca ovina
Lolium rigidum
Festuca a. arundinacea
Festuca gigantea
Festuca durandoi
Festuca paniculata
Festuca caldasii
Vulpia ciliata
Festuca chimborazensis
Festuca rubra
Festuca fenas
Lolium perenne
Festuca eskia
Lolium persicum
Festuca pratensis
Festuca asplundii
Festuca procera
Festuca triflora
Ty1_Copia_1Cx (Mbp)
2
1
0
−1
1Cx (Mbp)
2000
3000
4000
5000

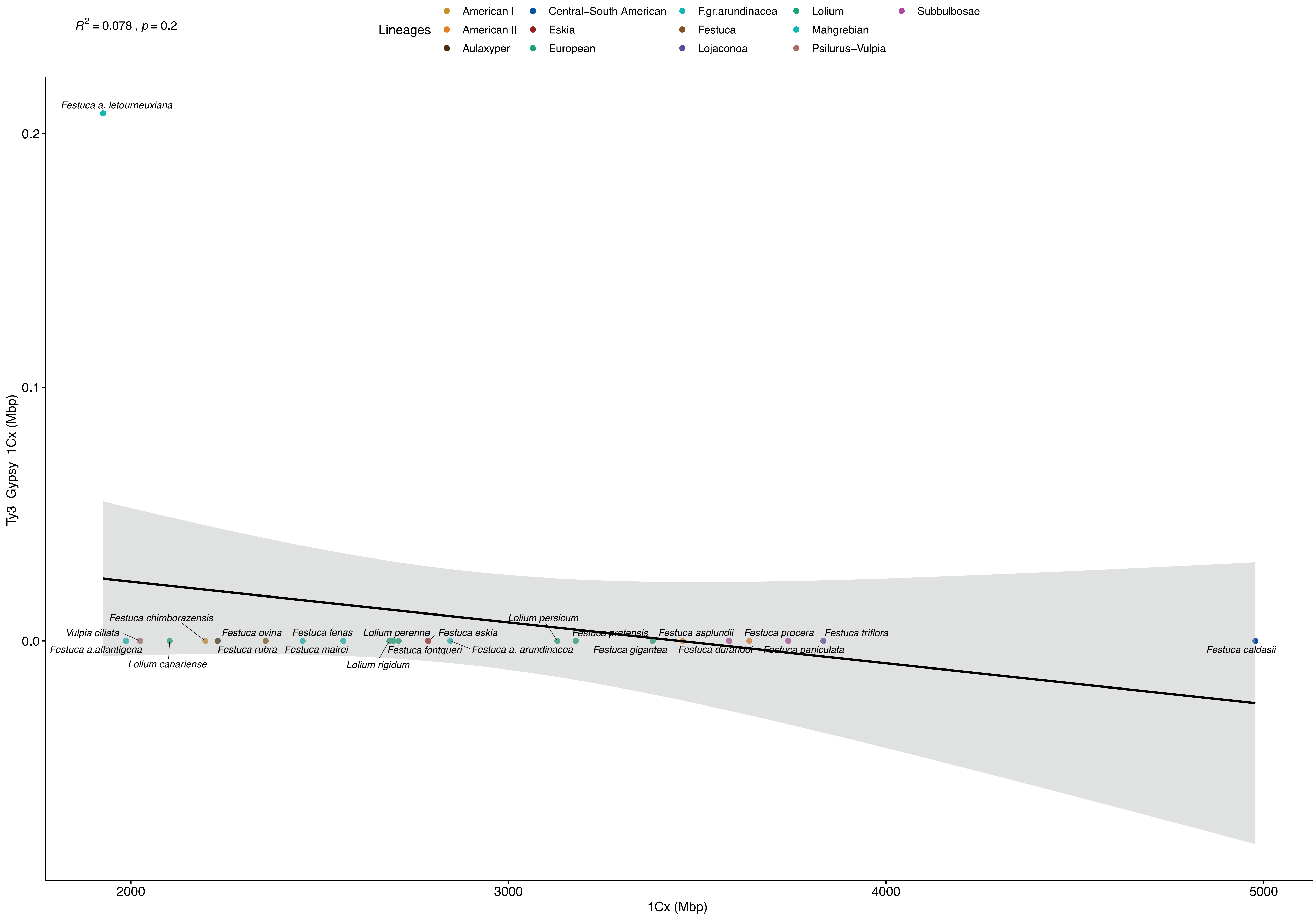

$R^2 = 0.078$, $p = 0.2$
Lineages
American I
American II
Aulaxyper
Central–South American
Eskia
European
F.gr.arundinacea
Festuca
Lojaconoa
Lolium
Mahgrebian
Psilurus–Vulpia
Subbulbosae
Ty3_Gypsy_1Cx (Mbp)
1Cx (Mbp)
0.2
0.1
0.0
2000
3000
4000
5000
Festuca a. letourneuxiana
Festuca chimborazensis
Vulpia ciliata
Festuca a.atlantigena
Lolium canariense
Festuca ovina
Festuca rubra
Festuca fenas
Festuca mairei
Lolium perenne
Lolium rigidum
Festuca eskia
Festuca fontqueri
Festuca a. arundinacea
Lolium persicum
Festuca pratensis
Festuca gigantea
Festuca asplundii
Festuca durandoi
Festuca procera
Festuca paniculata
Festuca triflora
Festuca caldasii

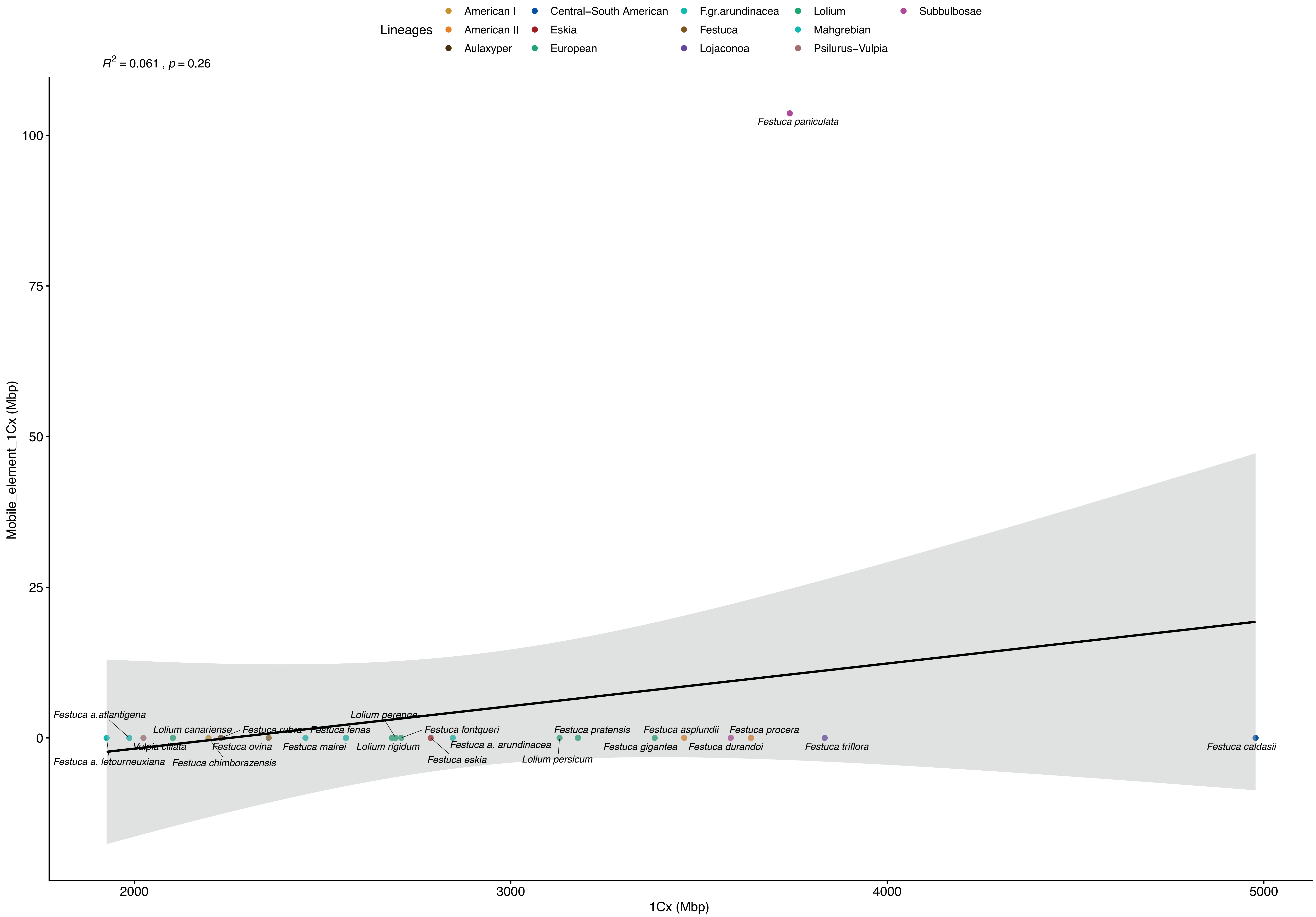

Lineages
American I
American II
Aulaxyper
Central–South American
Eskia
European
F.gr.arundinacea
Festuca
Lojaconoa
Lolium
Mahgrebian
Psilurus–Vulpia
Subbulbosae
$R^2 = 0.061$, $p = 0.26$
Festuca paniculata
Festuca a.atlantigena
Lolium canariense
Festuca rubra
Festuca fenas
Lolium perenne
Festuca fontqueri
Festuca pratensis
Festuca asplundii
Festuca procera
Vulpia ciliata
Festuca ovina
Festuca mairei
Lolium rigidum
Festuca a. arundinacea
Festuca gigantea
Festuca durandoi
Festuca triflora
Festuca caldasii
Festuca a. letourneuxiana
Festuca chimborazensis
Festuca eskia
Lolium persicum
Mobile_element_1Cx (Mbp)
1Cx (Mbp)
0
25
50
75
100
2000
3000
4000
5000

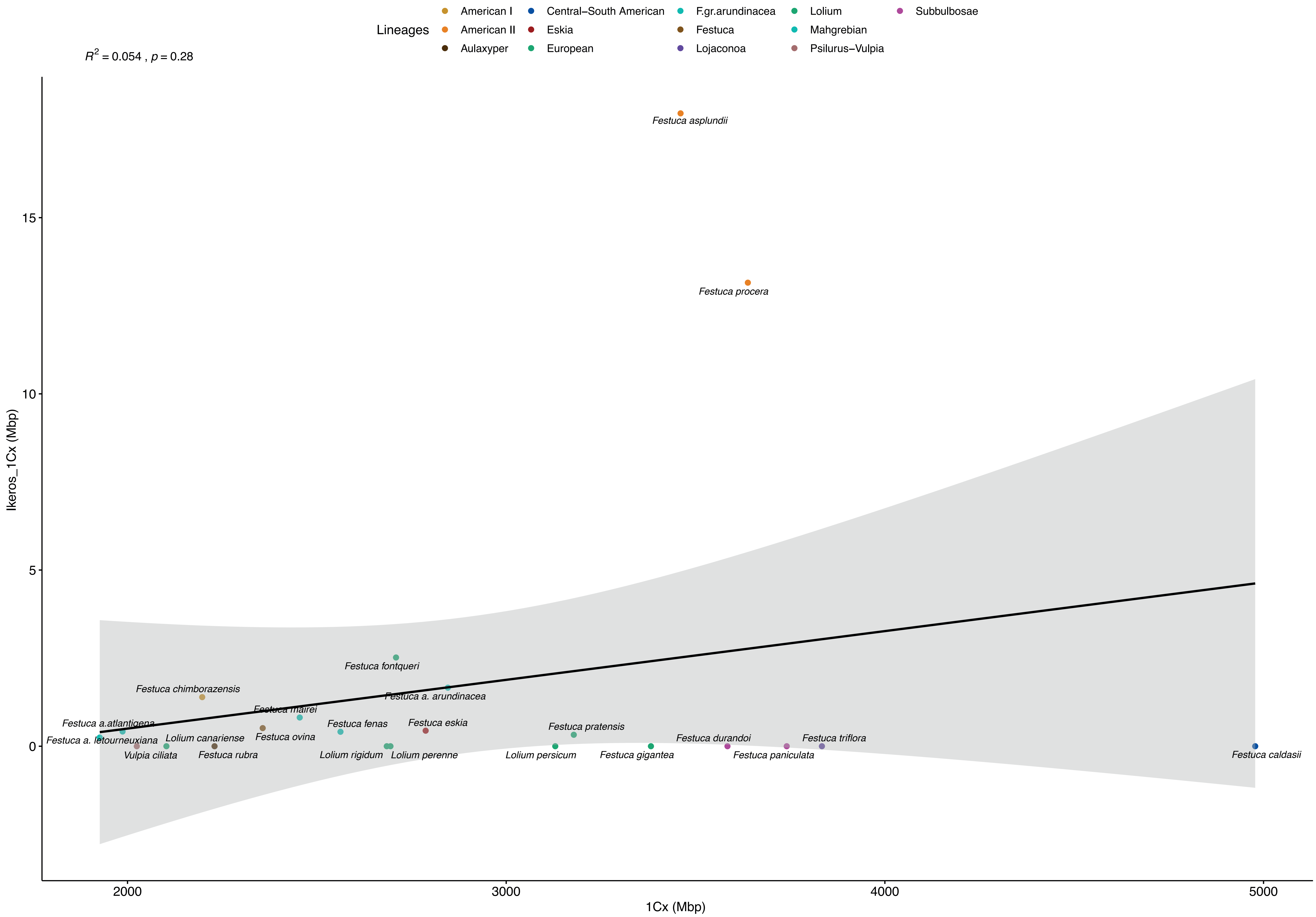

Lineages
American I
American II
Aulaxyper
Central–South American
Eskia
European
F.gr.arundinacea
Festuca
Lojaconoa
Lolium
Mahgrebian
Psilurus–Vulpia
Subbulbosae
$R^2 = 0.054$, $p = 0.28$
Ikeros_1Cx (Mbp)
1Cx (Mbp)
Festuca asplundii
Festuca procera
Festuca fontqueri
Festuca chimborazensis
Festuca a. arundinacea
Festuca mairei
Festuca a.atlantigena
Festuca fenas
Festuca eskia
Festuca pratensis
Festuca a. letourneuxiana
Lolium canariense
Festuca ovina
Festuca durandoi
Festuca triflora
Vulpia ciliata
Festuca rubra
Lolium rigidum
Lolium perenne
Lolium persicum
Festuca gigantea
Festuca paniculata
Festuca caldasii

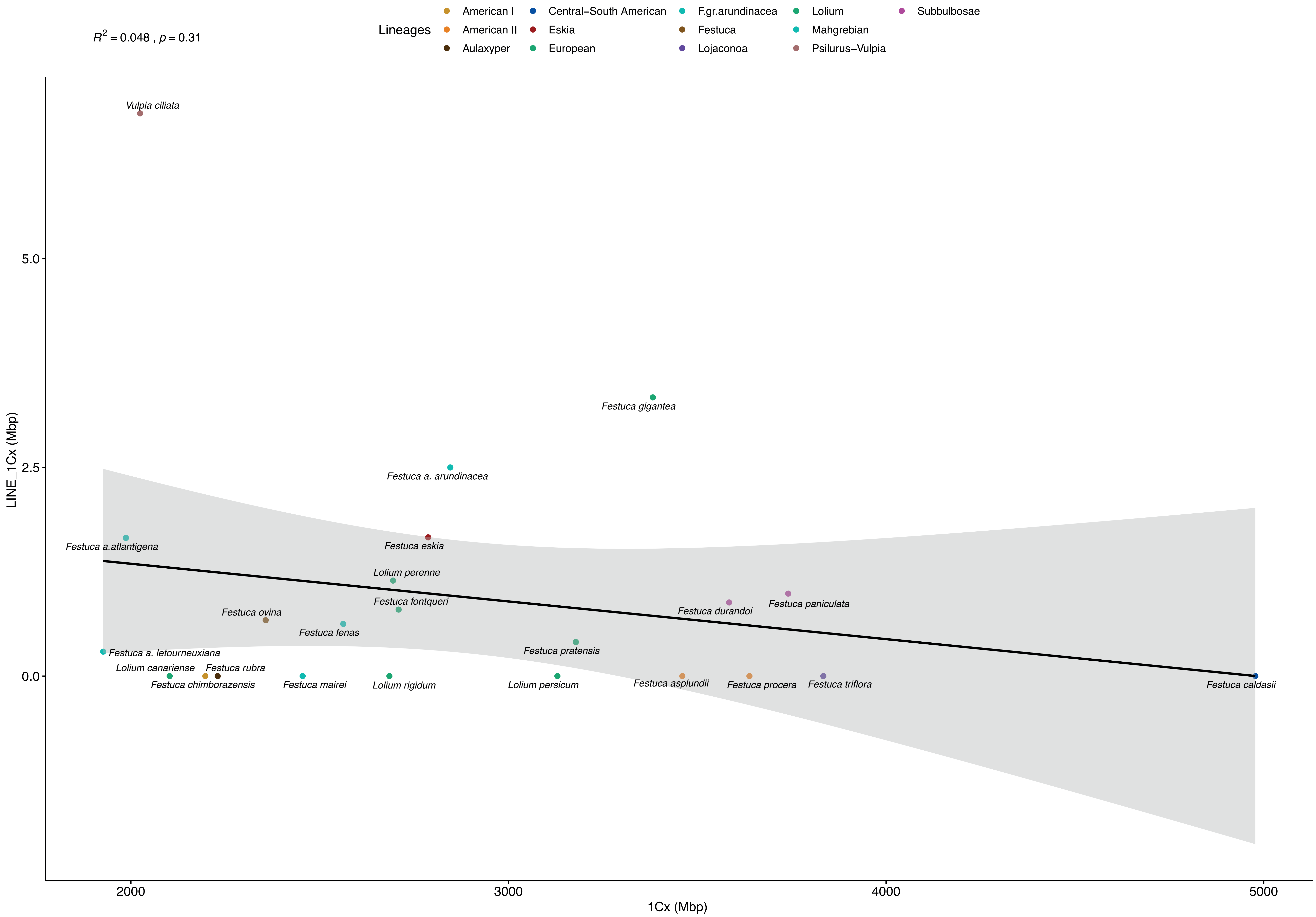

$R^2 = 0.048$, $p = 0.31$
Lineages
American I
American II
Aulaxyper
Central–South American
Eskia
European
F.gr.arundinacea
Festuca
Lojaconoa
Lolium
Mahgrebian
Psilurus–Vulpia
Subbulbosae
Vulpia ciliata
Festuca gigantea
Festuca a. arundinacea
Festuca a.atlantigena
Festuca eskia
Lolium perenne
Festuca fontqueri
Festuca ovina
Festuca fenas
Festuca durandoi
Festuca paniculata
Festuca pratensis
Festuca a. letourneuxiana
Lolium canariense
Festuca rubra
Festuca chimborazensis
Festuca mairei
Lolium rigidum
Lolium persicum
Festuca asplundii
Festuca procera
Festuca triflora
Festuca caldasii
LINE_1Cx (Mbp)
5.0
2.5
0.0
1Cx (Mbp)
2000
3000
4000
5000

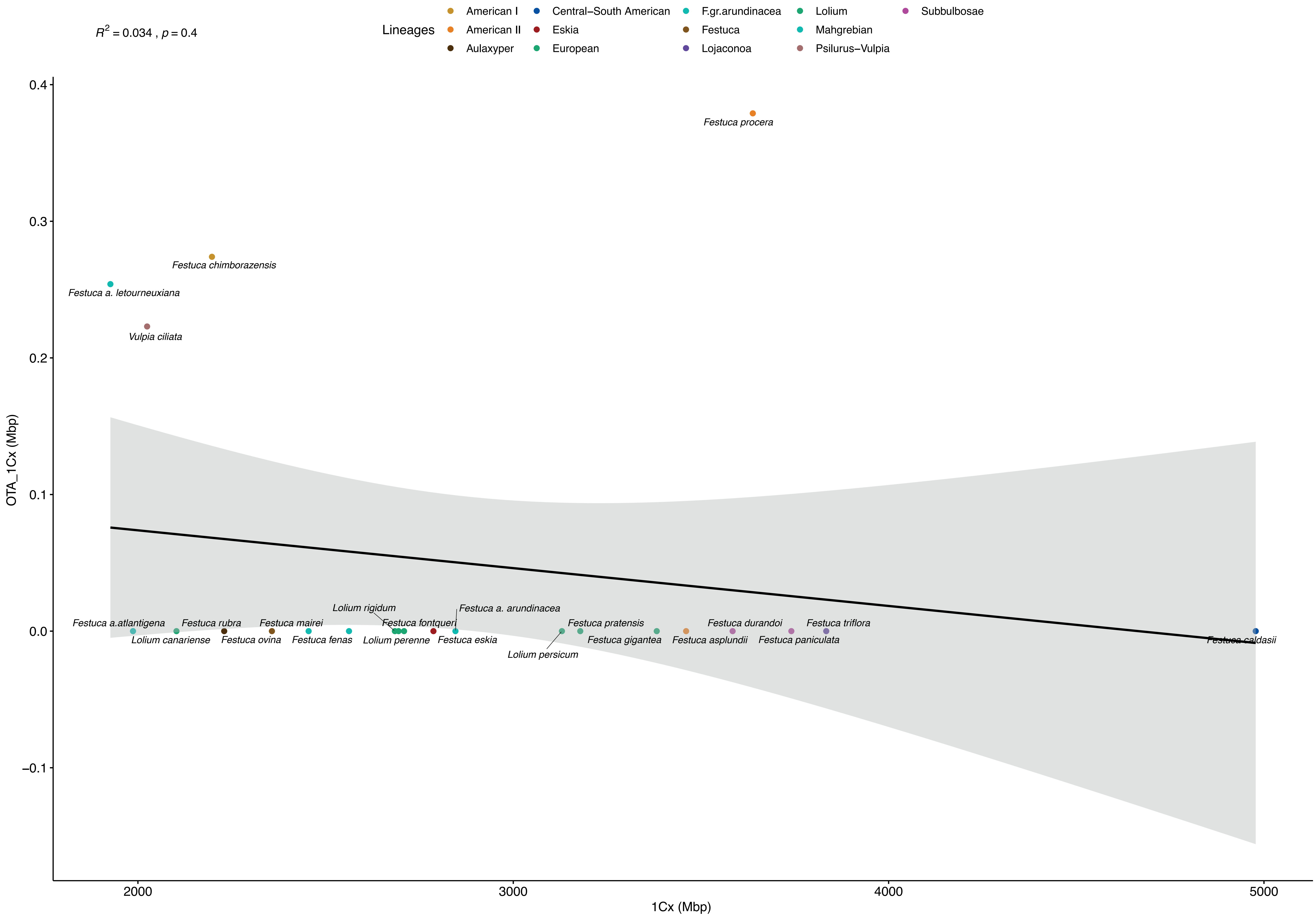

$R^2 = 0.034$, $p = 0.4$
Lineages
American I
American II
Aulaxyper
Central–South American
Eskia
European
F.gr.arundinacea
Festuca
Lojaconoa
Lolium
Mahgrebian
Psilurus–Vulpia
Subbulbosae
Festuca procera
Festuca chimborazensis
Festuca a. letourneuxiana
Vulpia ciliata
Festuca a.atlantigena
Lolium canariense
Festuca rubra
Festuca ovina
Festuca mairei
Festuca fenas
Lolium rigidum
Festuca fontqueri
Lolium perenne
Festuca a. arundinacea
Festuca eskia
Lolium persicum
Festuca pratensis
Festuca gigantea
Festuca asplundii
Festuca durandoi
Festuca paniculata
Festuca triflora
Festuca caldasii
OTA_1Cx (Mbp)
1Cx (Mbp)

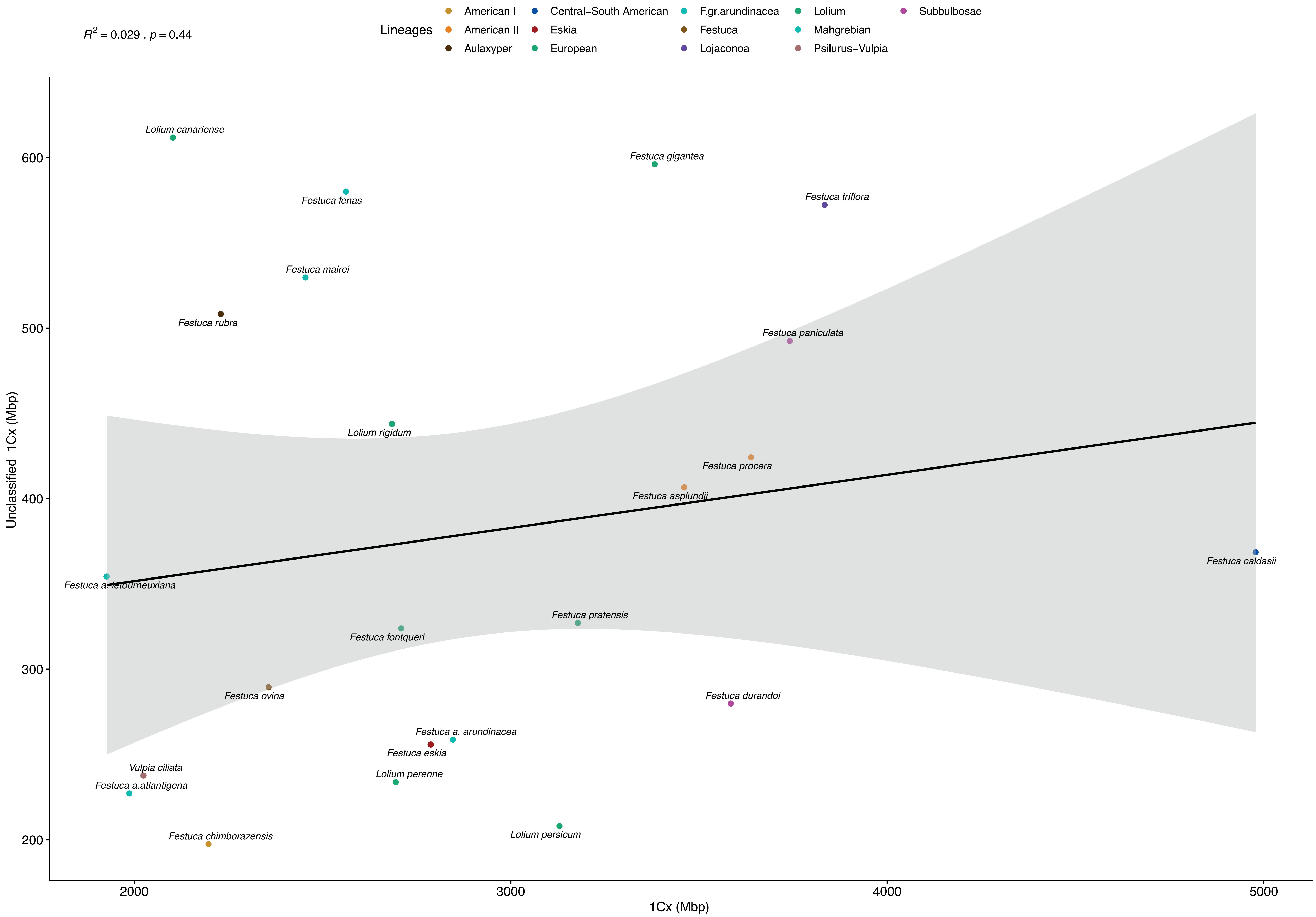

$R^2 = 0.029$, $p = 0.44$
Lineages
American I
American II
Aulaxyper
Central–South American
Eskia
European
F.gr.arundinacea
Festuca
Lojaconoa
Lolium
Mahgrebian
Psilurus–Vulpia
Subbulbosae
Unclassified_1Cx (Mbp)
1Cx (Mbp)
200
300
400
500
600
2000
3000
4000
5000
Lolium canariense
Festuca gigantea
Festuca fenas
Festuca triflora
Festuca mairei
Festuca rubra
Festuca paniculata
Lolium rigidum
Festuca procera
Festuca asplundii
Festuca caldasii
Festuca a. letourneuxiana
Festuca pratensis
Festuca fontqueri
Festuca ovina
Festuca durandoi
Festuca a. arundinacea
Festuca eskia
Vulpia ciliata
Festuca a.atlantigena
Lolium perenne
Lolium persicum
Festuca chimborazensis

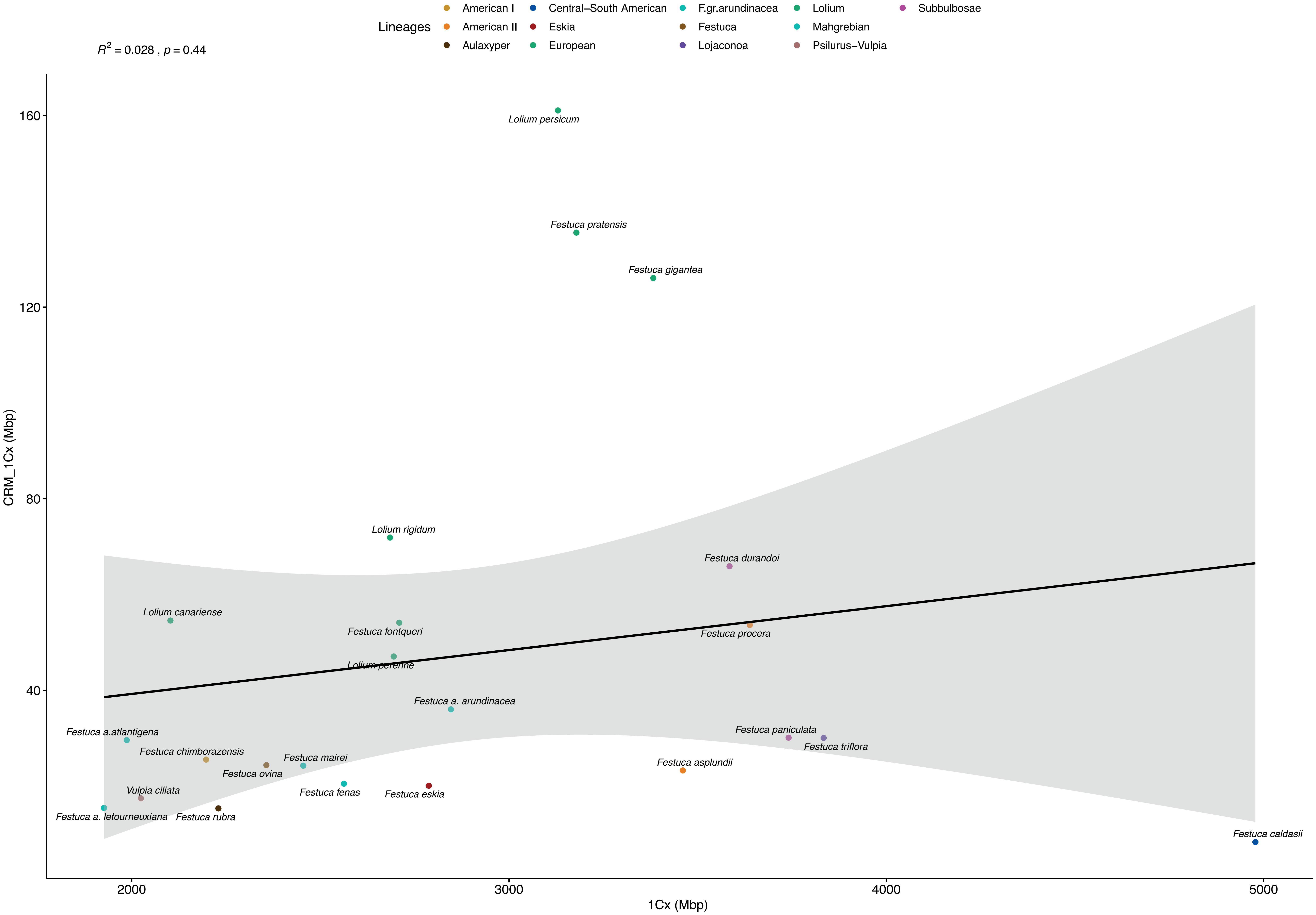

Lineages
American I
American II
Aulaxyper
Central–South American
Eskia
European
F.gr.arundinacea
Festuca
Lojaconoa
Lolium
Mahgrebian
Psilurus–Vulpia
Subbulbosae
$R^2 = 0.028$, $p = 0.44$
160
120
80
40
CRM_1Cx (Mbp)
2000
3000
4000
5000
1Cx (Mbp)
Lolium persicum
Festuca pratensis
Festuca gigantea
Lolium rigidum
Festuca durandoi
Lolium canariense
Festuca fontqueri
Festuca procera
Lolium perenne
Festuca a. arundinacea
Festuca a.atlantigena
Festuca paniculata
Festuca triflora
Festuca chimborazensis
Festuca mairei
Festuca ovina
Festuca asplundii
Festuca fenas
Festuca eskia
Vulpia ciliata
Festuca a. letourneuxiana
Festuca rubra
Festuca caldasii

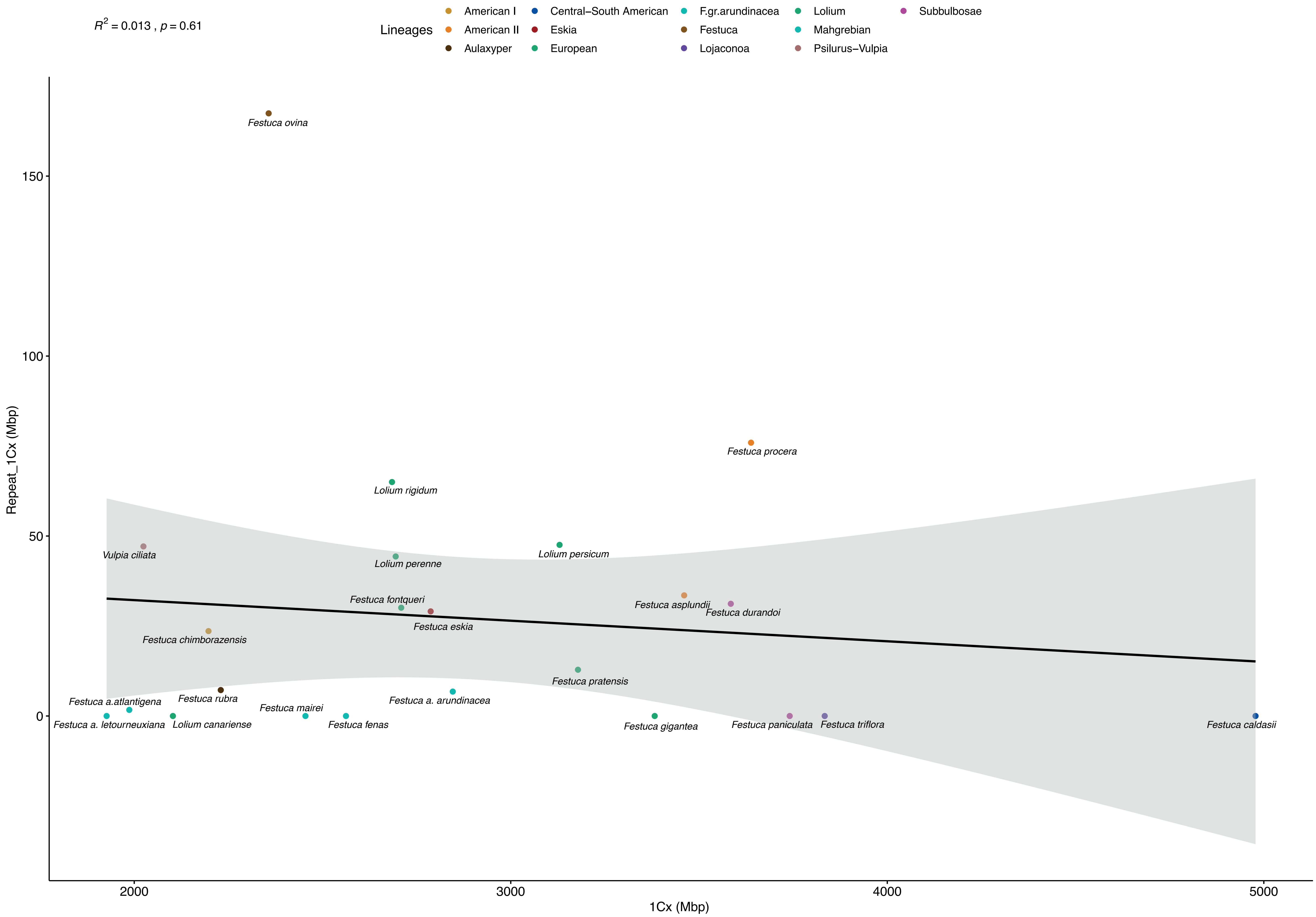
$R^2 = 0.013$, $p = 0.61$
Lineages
American I
American II
Aulaxyper
Central–South American
Eskia
European
F.gr.arundinacea
Festuca
Lojaconoa
Lolium
Mahgrebian
Psilurus–Vulpia
Subbulbosae
Festuca ovina
Festuca procera
Lolium rigidum
Vulpia ciliata
Lolium perenne
Lolium persicum
Festuca fontqueri
Festuca eskia
Festuca asplundii
Festuca durandoi
Festuca chimborazensis
Festuca pratensis
Festuca rubra
Festuca a.atlantigena
Festuca mairei
Festuca a. arundinacea
Festuca a. letourneuxiana
Lolium canariense
Festuca fenas
Festuca gigantea
Festuca paniculata
Festuca triflora
Festuca caldasii
Repeat_1Cx (Mbp)
150
100
50
0
1Cx (Mbp)
2000
3000
4000
5000

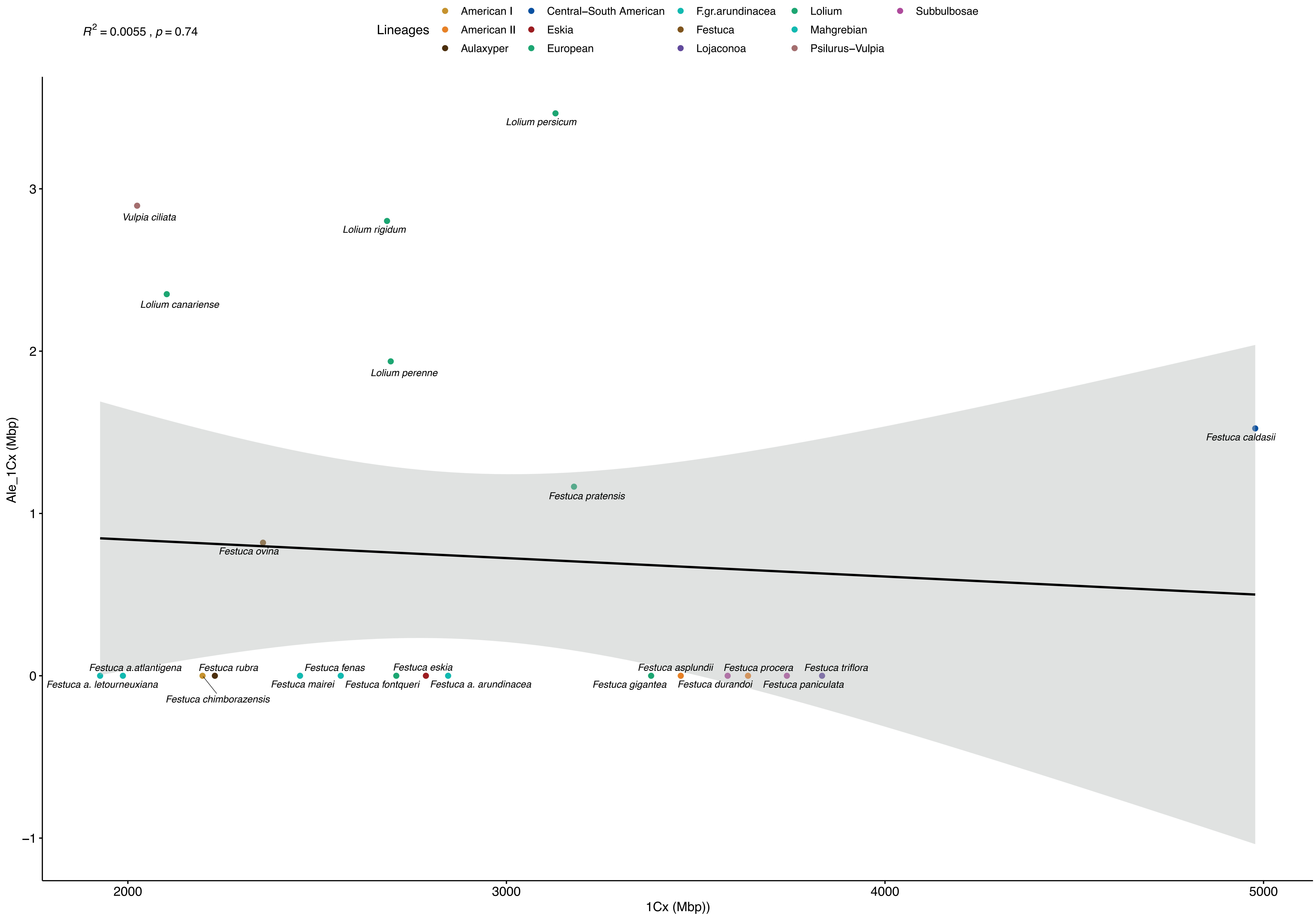

$R^2 = 0.0055$, $p = 0.74$
Lineages
American I
American II
Aulaxyper
Central–South American
Eskia
European
F.gr.arundinacea
Festuca
Lojaconoa
Lolium
Mahgrebian
Psilurus–Vulpia
Subbulbosae
Lolium persicum
Vulpia ciliata
Lolium rigidum
Lolium canariense
Lolium perenne
Festuca caldasii
Festuca pratensis
Festuca ovina
Festuca a.atlantigena
Festuca a. letourneuxiana
Festuca rubra
Festuca chimborazensis
Festuca fenas
Festuca mairei
Festuca eskia
Festuca fontqueri
Festuca a. arundinacea
Festuca asplundii
Festuca gigantea
Festuca procera
Festuca durandoi
Festuca triflora
Festuca paniculata
Ale_1Cx (Mbp)
1Cx (Mbp))

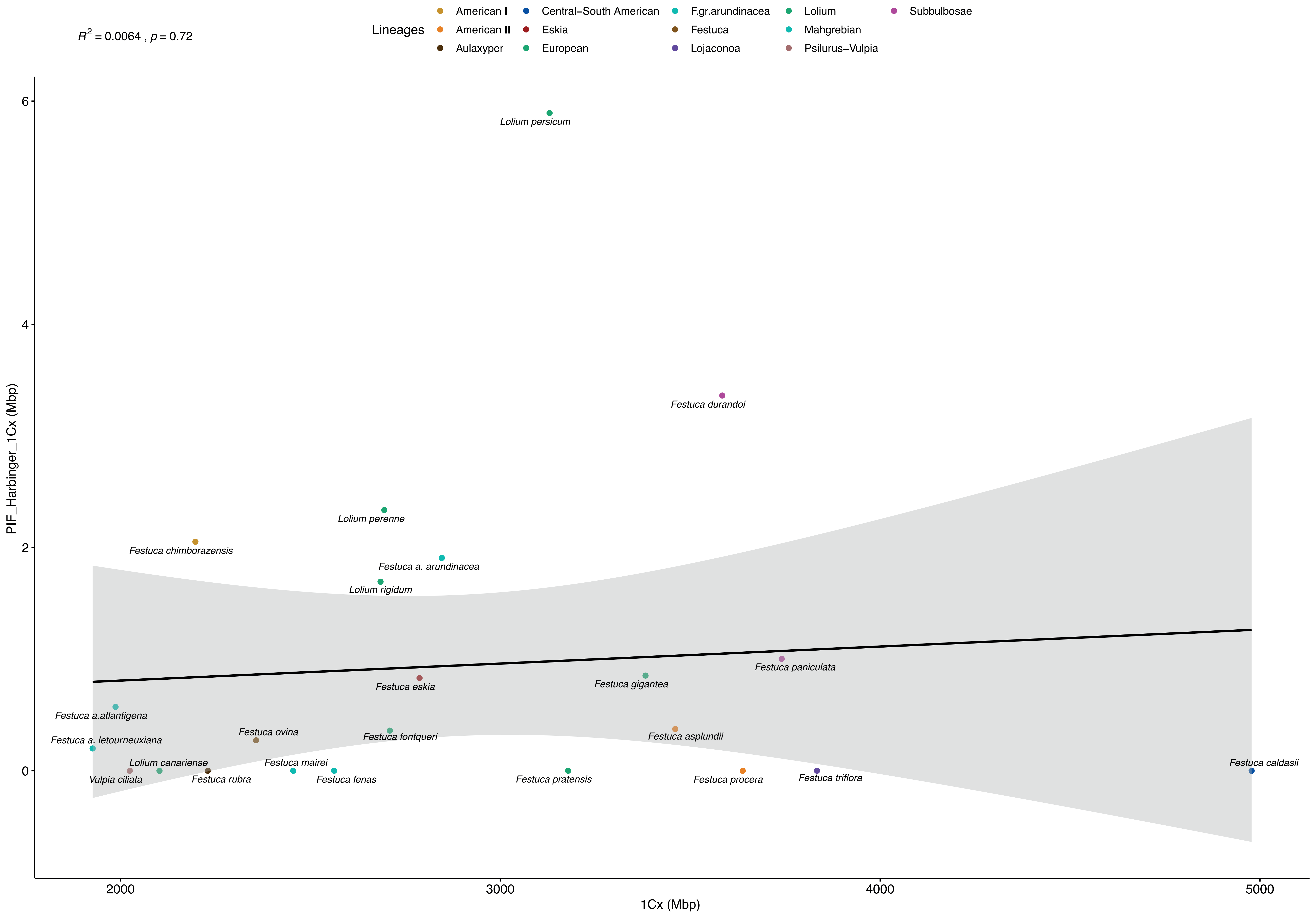

$R^2 = 0.0064$, $p = 0.72$
Lineages
American I
American II
Aulaxyper
Central−South American
Eskia
European
F.gr.arundinacea
Festuca
Lojaconoa
Lolium
Mahgrebian
Psilurus−Vulpia
Subbulbosae
PIF_Harbinger_1Cx (Mbp)
1Cx (Mbp)
Lolium persicum
Festuca durandoi
Lolium perenne
Festuca chimborazensis
Festuca a. arundinacea
Lolium rigidum
Festuca paniculata
Festuca eskia
Festuca gigantea
Festuca a.atlantigena
Festuca a. letourneuxiana
Festuca ovina
Festuca fontqueri
Festuca asplundii
Lolium canariense
Festuca mairei
Festuca caldasii
Vulpia ciliata
Festuca rubra
Festuca fenas
Festuca pratensis
Festuca procera
Festuca triflora

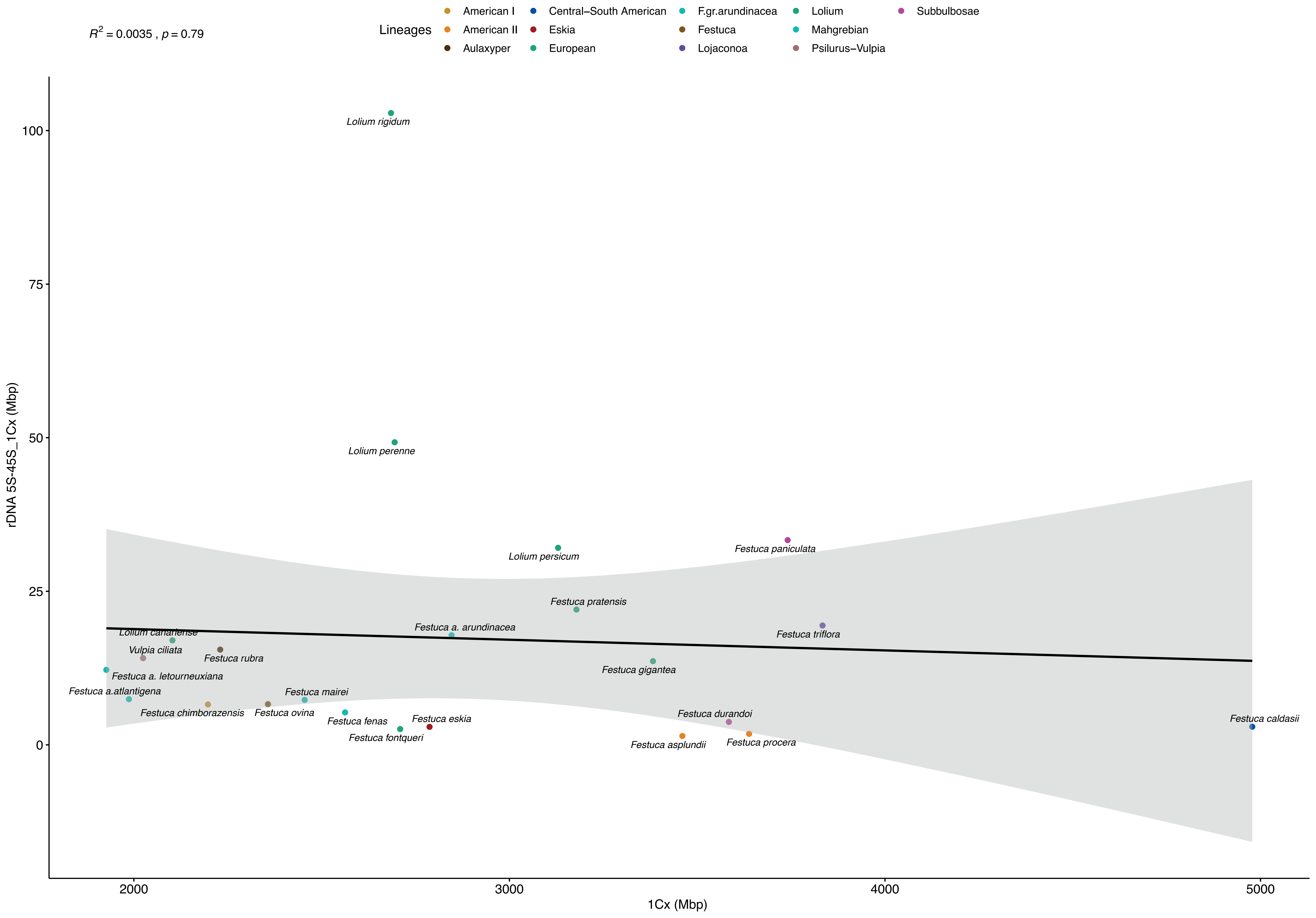

$R^2 = 0.0035$, $p = 0.79$
Lineages
American I
American II
Aulaxyper
Central–South American
Eskia
European
F.gr.arundinacea
Festuca
Lojaconoa
Lolium
Mahgrebian
Psilurus–Vulpia
Subbulbosae
rDNA 5S-45S_1Cx (Mbp)
1Cx (Mbp)
0
25
50
75
100
2000
3000
4000
5000
Lolium rigidum
Lolium perenne
Lolium persicum
Festuca paniculata
Festuca pratensis
Festuca a. arundinacea
Festuca triflora
Lolium canariense
Vulpia ciliata
Festuca rubra
Festuca gigantea
Festuca a. letourneuxiana
Festuca a.atlantigena
Festuca mairei
Festuca chimborazensis
Festuca ovina
Festuca fenas
Festuca eskia
Festuca fontqueri
Festuca durandoi
Festuca caldasii
Festuca asplundii
Festuca procera

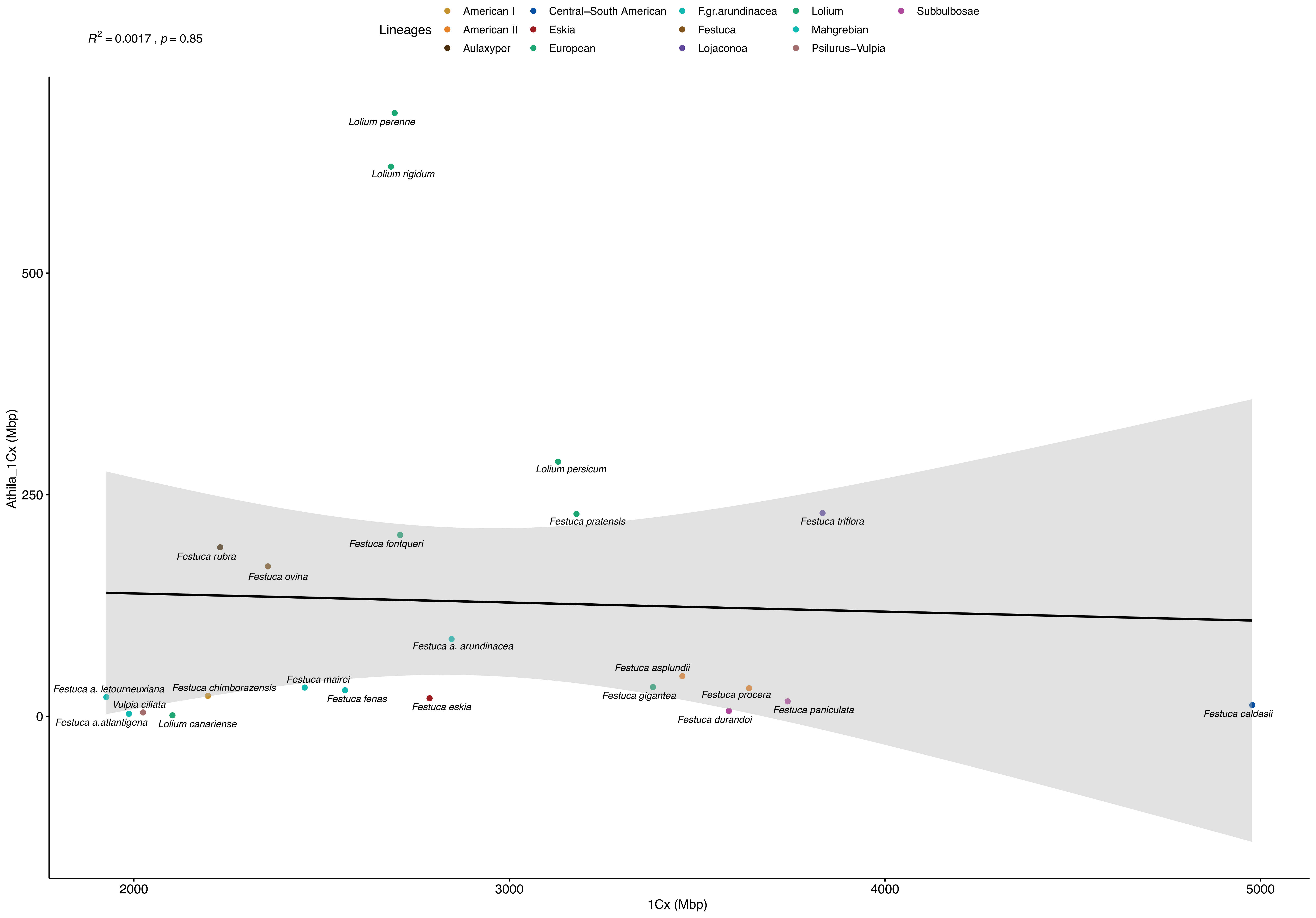
$R^2 = 0.0017$, $p = 0.85$
Lineages
American I
American II
Aulaxyper
Central–South American
Eskia
European
F.gr.arundinacea
Festuca
Lojaconoa
Lolium
Mahgrebian
Psilurus–Vulpia
Subbulbosae
Lolium perenne
Lolium rigidum
Lolium persicum
Festuca pratensis
Festuca triflora
Festuca fontqueri
Festuca rubra
Festuca ovina
Festuca a. arundinacea
Festuca asplundii
Festuca a. letourneuxiana
Festuca chimborazensis
Festuca mairei
Festuca fenas
Festuca eskia
Festuca gigantea
Festuca procera
Festuca paniculata
Vulpia ciliata
Festuca a.atlantigena
Lolium canariense
Festuca durandoi
Festuca caldasii
Athila_1Cx (Mbp)
500
250
0
2000
3000
4000
5000
1Cx (Mbp)

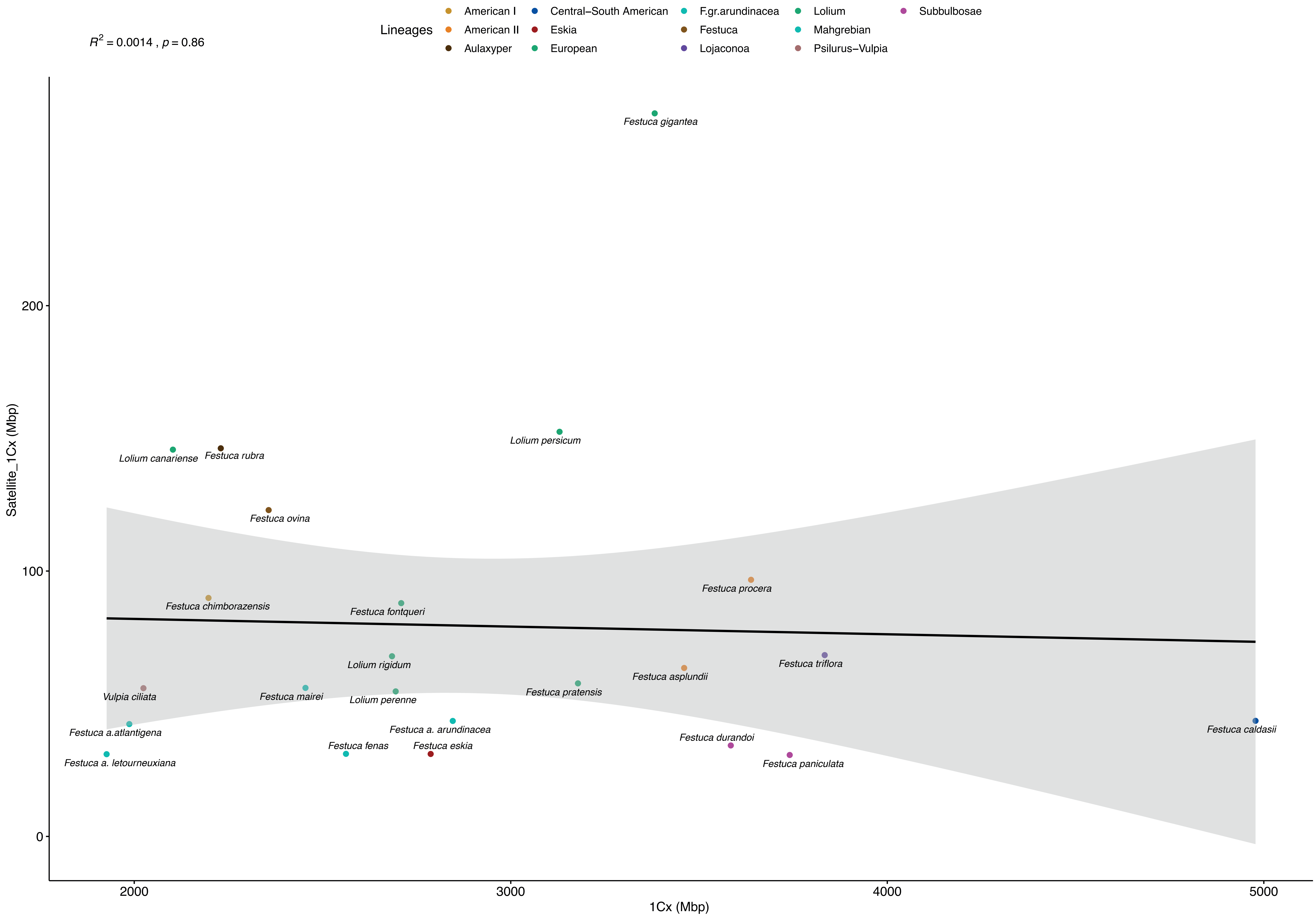

$R^2 = 0.0014$, $p = 0.86$
Lineages
American I
American II
Aulaxyper
Central–South American
Eskia
European
F.gr.arundinacea
Festuca
Lojaconoa
Lolium
Mahgrebian
Psilurus–Vulpia
Subbulbosae
Festuca gigantea
Lolium persicum
Lolium canariense
Festuca rubra
Festuca ovina
Festuca procera
Festuca chimborazensis
Festuca fontqueri
Lolium rigidum
Festuca triflora
Festuca asplundii
Vulpia ciliata
Festuca mairei
Lolium perenne
Festuca pratensis
Festuca a. arundinacea
Festuca caldasii
Festuca a.atlantigena
Festuca fenas
Festuca eskia
Festuca durandoi
Festuca a. letourneuxiana
Festuca paniculata
Satellite_1Cx (Mbp)
1Cx (Mbp)
0
100
200
2000
3000
4000
5000

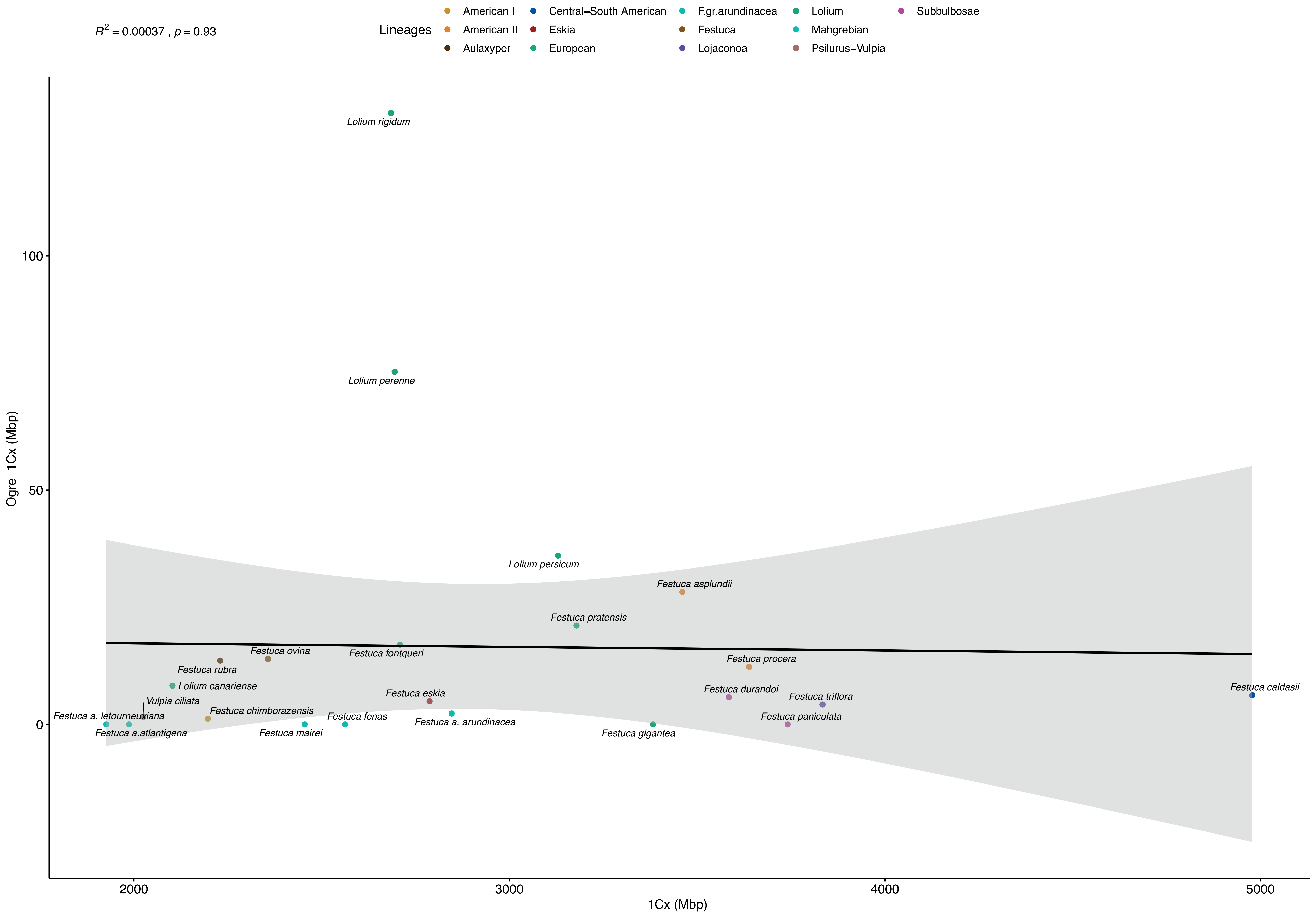

$R^2 = 0.00037$, $p = 0.93$
Lineages
American I
American II
Aulaxyper
Central–South American
Eskia
European
F.gr.arundinacea
Festuca
Lojaconoa
Lolium
Mahgrebian
Psilurus–Vulpia
Subbulbosae
Lolium rigidum
Lolium perenne
Lolium persicum
Festuca asplundii
Festuca pratensis
Festuca ovina
Festuca fontqueri
Festuca rubra
Festuca procera
Lolium canariense
Festuca eskia
Festuca durandoi
Festuca caldasii
Festuca triflora
Vulpia ciliata
Festuca chimborazensis
Festuca a. letourneuxiana
Festuca fenas
Festuca a. arundinacea
Festuca paniculata
Festuca a.atlantigena
Festuca mairei
Festuca gigantea
Ogre_1Cx (Mbp)
100
50
0
2000
3000
4000
5000
1Cx (Mbp)

Suppl. Fig. S3A

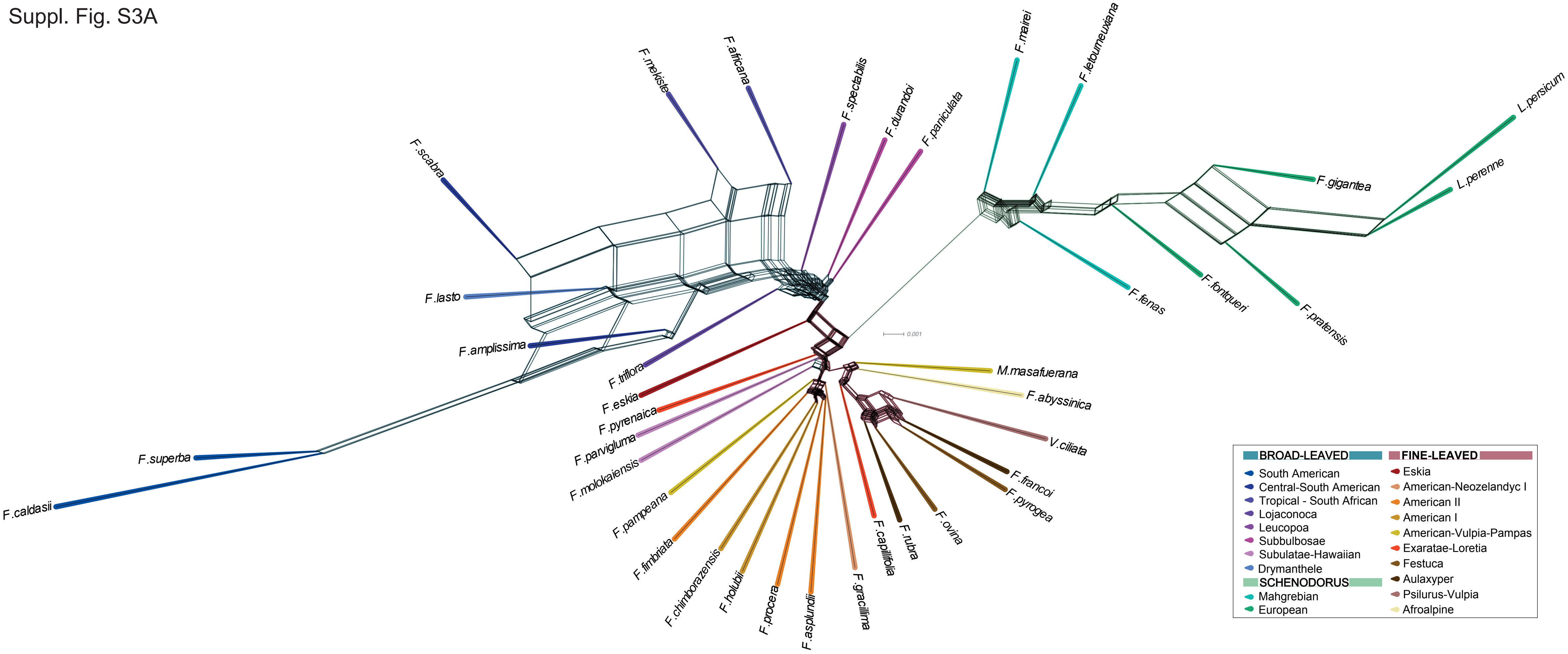

Suppl. Fig. S3B

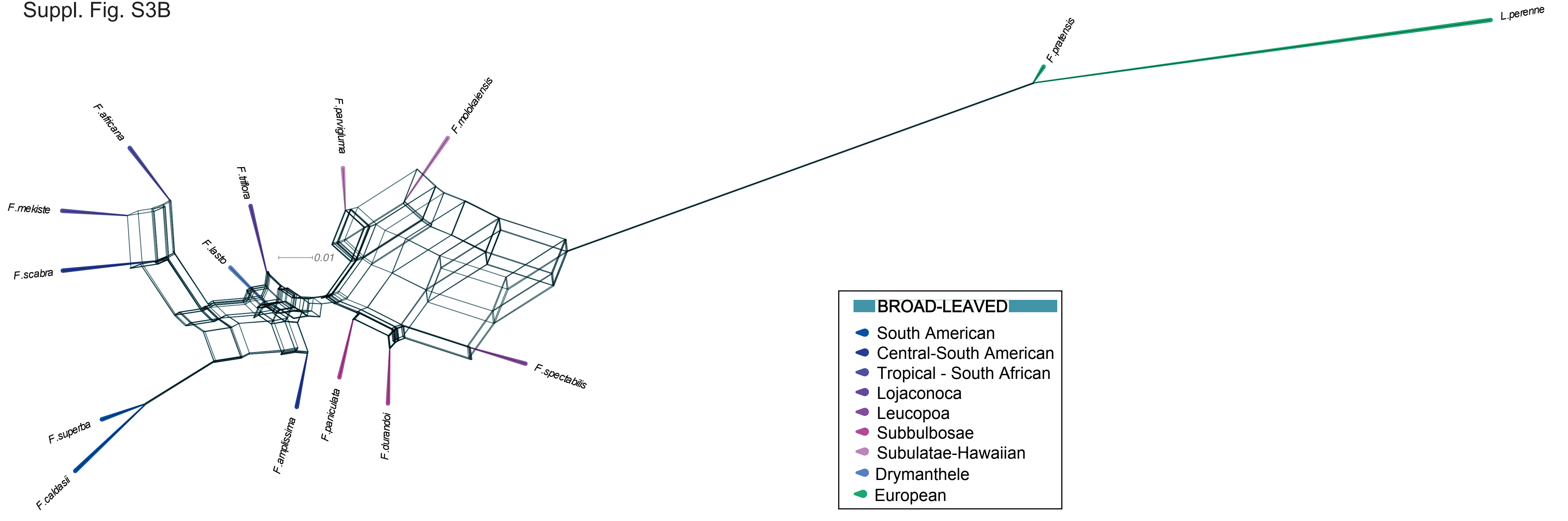

Suppl. Fig. S3C

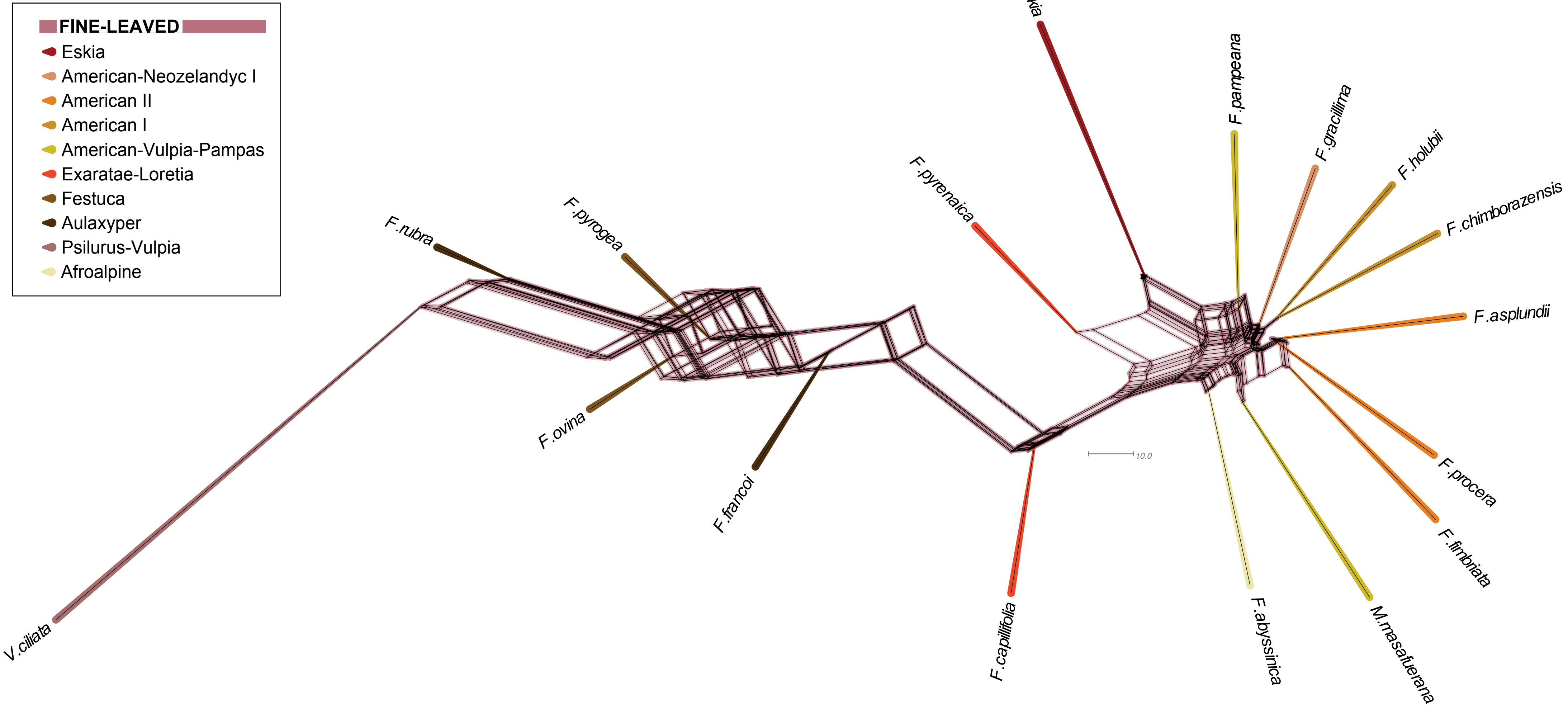

Suppl. Fig, S3D

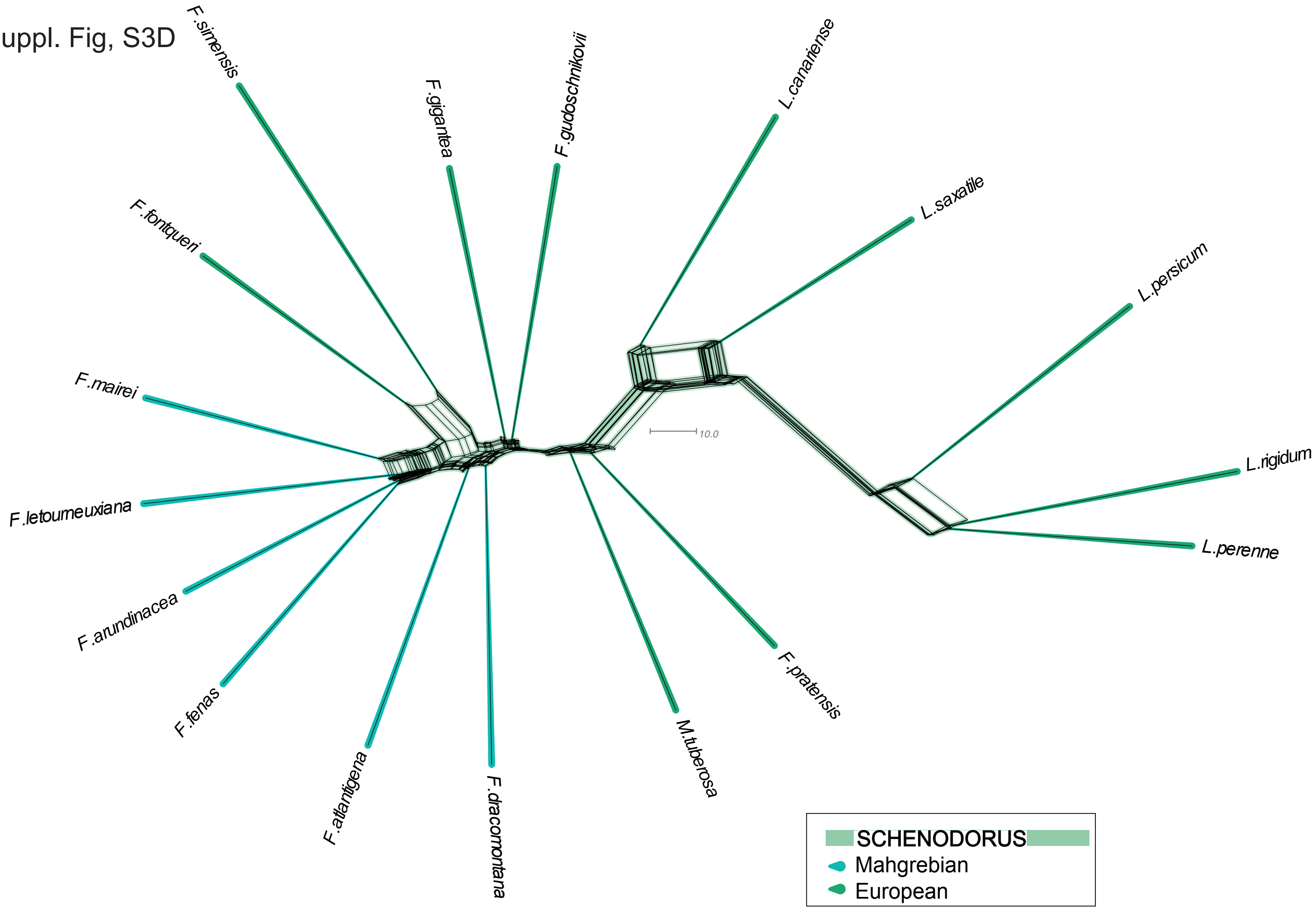

Suppl. Fig. S4

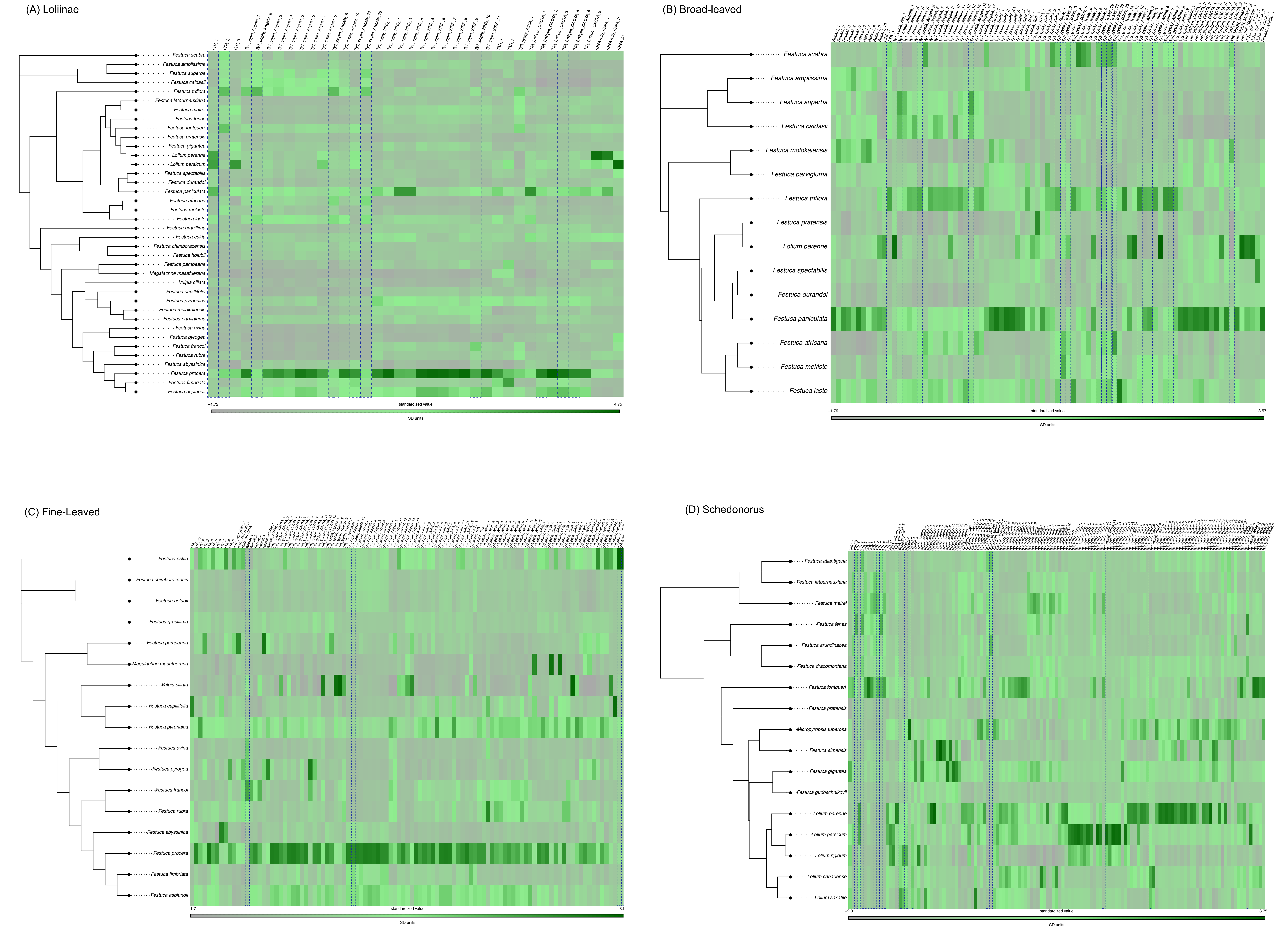
(A) Loliinae
Festuca scabra
Festuca amplissima
Festuca superba
Festuca caldasii
Festuca triflora
Festuca letourneuxiana
Festuca mairei
Festuca fenas
Festuca fontqueri
Festuca pratensis
Festuca gigantea
Lolium perenne
Lolium persicum
Festuca spectabilis
Festuca durandoi
Festuca paniculata
Festuca africana
Festuca mekiste
Festuca lasto
Festuca gracillima
Festuca eskia
Festuca chimborazensis
Festuca holubii
Festuca pampeana
Megalachne masafuerana
Vulpia ciliata
Festuca capillifolia
Festuca pyrenaica
Festuca molokaiensis
Festuca parvigluma
Festuca ovina
Festuca pyrogea
Festuca francoi
Festuca rubra
Festuca abyssinica
Festuca procera
Festuca fimbriata
Festuca asplundii
−1.72
standardized value
4.75
SD units
(B) Broad-leaved
Festuca scabra
Festuca amplissima
Festuca superba
Festuca caldasii
Festuca molokaiensis
Festuca parvigluma
Festuca triflora
Festuca pratensis
Lolium perenne
Festuca spectabilis
Festuca durandoi
Festuca paniculata
Festuca africana
Festuca mekiste
Festuca lasto
−1.79
standardized value
3.57
SD units
(C) Fine-Leaved
Festuca eskia
Festuca chimborazensis
Festuca holubii
Festuca gracillima
Festuca pampeana
Megalachne masafuerana
Vulpia ciliata
Festuca capillifolia
Festuca pyrenaica
Festuca ovina
Festuca pyrogea
Festuca francoi
Festuca rubra
Festuca abyssinica
Festuca procera
Festuca fimbriata
Festuca asplundii
−1.7
standardized value
SD units
(D) Schedonorus
Festuca atlantigena
Festuca letourneuxiana
Festuca mairei
Festuca fenas
Festuca arundinacea
Festuca dracomontana
Festuca fontqueri
Festuca pratensis
Micropyropsis tuberosa
Festuca simensis
Festuca gigantea
Festuca gudoschnikovii
Lolium perenne
Lolium persicum
Lolium rigidum
Lolium canariense
Lolium saxatile
−2.01
standardized value
3.75
SD units

Suppl. Fig. S5

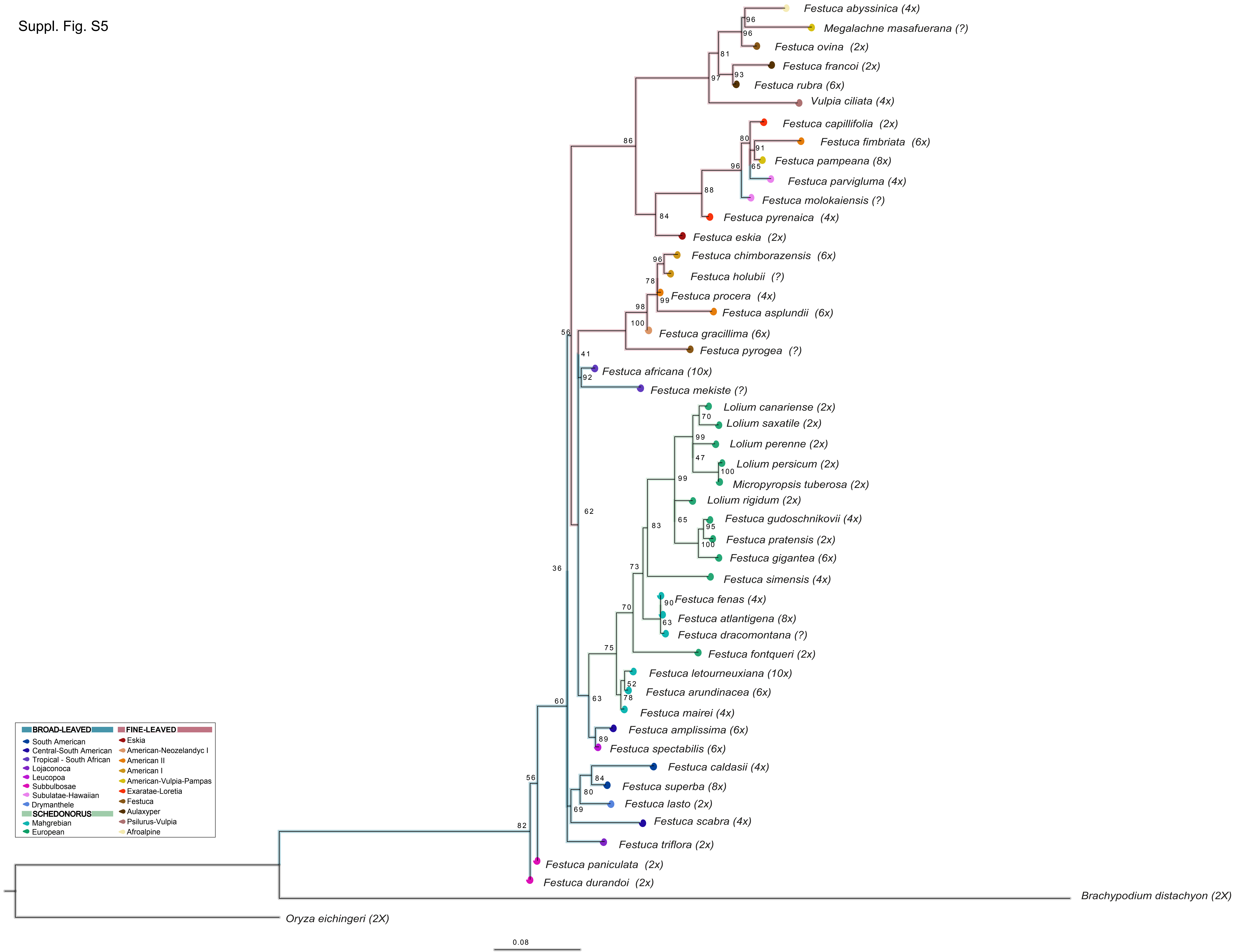

Festuca abyssinica (4x)
Megalachne masafuerana (?)
Festuca ovina (2x)
Festuca francoi (2x)
Festuca rubra (6x)
Vulpia ciliata (4x)
Festuca capillifolia (2x)
Festuca fimbriata (6x)
Festuca pampeana (8x)
Festuca parvigluma (4x)
Festuca molokaiensis (?)
Festuca pyrenaica (4x)
Festuca eskia (2x)
Festuca chimborazensis (6x)
Festuca holubii (?)
Festuca procera (4x)
Festuca asplundii (6x)
Festuca gracillima (6x)
Festuca pyrogea (?)
Festuca africana (10x)
Festuca mekiste (?)
Lolium canariense (2x)
Lolium saxatile (2x)
Lolium perenne (2x)
Lolium persicum (2x)
Micropyropsis tuberosa (2x)
Lolium rigidum (2x)
Festuca gudoschnikovii (4x)
Festuca pratensis (2x)
Festuca gigantea (6x)
Festuca simensis (4x)
Festuca fenas (4x)
Festuca atlantigena (8x)
Festuca dracomontana (?)
Festuca fontqueri (2x)
Festuca letourneuxiana (10x)
Festuca arundinacea (6x)
Festuca mairei (4x)
Festuca amplissima (6x)
Festuca spectabilis (6x)
Festuca caldasii (4x)
Festuca superba (8x)
Festuca lasto (2x)
Festuca scabra (4x)
Festuca triflora (2x)
Festuca paniculata (2x)
Festuca durandoi (2x)
Brachypodium distachyon (2X)
Oryza eichingeri (2X)
0.08
BROAD-LEAVED
South American
Central-South American
Tropical - South African
Lojaconoca
Leucopoa
Subbulbosae
Subulatae-Hawaiian
Drymanthele
SCHEDONORUS
Mahgrebian
European
FINE-LEAVED
Eskia
American-Neozelandyc I
American II
American I
American-Vulpia-Pampas
Exaratae-Loretia
Festuca
Aulaxyper
Psilurus-Vulpia
Afroalpine
